# Anemia and iron metabolism in COVID-19: a systematic review and meta-analysis

**DOI:** 10.1007/s10654-020-00678-5

**Published:** 2020-08-20

**Authors:** Petek Eylul Taneri, Sergio Alejandro Gómez-Ochoa, Erand Llanaj, Peter Francis Raguindin, Lyda Z. Rojas, Zayne Milena Roa-Díaz, Dante Salvador, Dion Groothof, Beatrice Minder, Doris Kopp-Heim, Wolf E. Hautz, Michele F. Eisenga, Oscar H. Franco, Marija Glisic, Taulant Muka

**Affiliations:** 1 Public Health Department, Faculty of Medicine, Bahcesehir University, Istanbul, Turkey; 2 Public Health and Epidemiological Studies Group, Cardiovascular Foundation of Colombia, Floridablanca, Colombia; 3 Doctoral School of Health Sciences, University of Debrecen, Debrecen, Hungary; 4 Institute of Social and Preventive Medicine (ISPM), University of Bern, Mittelstrasse 43, 3012 Bern, Switzerland; 5 Institute of Child Health and Human Development (ICHHD), National Institutes of Health, University of the Philippines Manila, Manila, Philippines; 6 Swiss Paraplegic Research, Nottwil, Switzerland; 7 Research Group and Development of Nursing Knowledge (GIDCEN-FCV), Research Institute, Cardiovascular Foundation of Colombia, Floriadablanca, Santander, Colombia; 8 Department of Internal Medicine, Division of Nephrology, University Medical Center Groningen, University of Groningen, Groningen, The Netherlands; 9 Public Health & Primary Care Library, University Library of Bern, University of Bern, Bern, Switzerland; 10 Department of Emergency Medicine, Inselspital, Bern University Hospital, University of Bern, Bern, Switzerland; 11 Public Health Research Institute, University of Debrecen, Debrecen, Hungary

**Keywords:** Anemia, Hemoglobin, Ferritin, Iron, Covid-19, Prognosis

## Abstract

Iron metabolism and anemia may play an important role in multiple organ dysfunction syndrome in Coronavirus disease 2019 (COVID-19). We conducted a systematic review and meta-analysis to evaluate biomarkers of anemia and iron metabolism (hemoglobin, ferritin, transferrin, soluble transferrin receptor, hepcidin, haptoglobin, unsaturated iron-binding capacity, erythropoietin, free erythrocyte protoporphyrine, and erythrocyte indices) in patients diagnosed with COVID-19, and explored their prognostic value. Six bibliographic databases were searched up to August 3rd 2020. We included 189 unique studies, with data from 57,563 COVID-19 patients. Pooled mean hemoglobin and ferritin levels in COVID-19 patients across all ages were 129.7 g/L (95% Confidence Interval (CI), 128.51; 130.88) and 777.33 ng/mL (95% CI, 701.33; 852.77), respectively. Hemoglobin levels were lower with older age, higher percentage of subjects with diabetes, hypertension and overall comorbidities, and admitted to intensive care. Ferritin level increased with older age, increasing proportion of hypertensive study participants, and increasing proportion of mortality. Compared to moderate cases, severe COVID-19 cases had lower hemoglobin [weighted mean difference (WMD), − 4.08 g/L (95% CI − 5.12; − 3.05)] and red blood cell count [WMD, − 0.16 × 10^12^ /L (95% CI − 0.31; − 0.014)], and higher ferritin [WMD, − 473.25 ng/mL (95% CI 382.52; 563.98)] and red cell distribution width [WMD, 1.82% (95% CI 0.10; 3.55)]. A significant difference in mean ferritin levels of 606.37 ng/mL (95% CI 461.86; 750.88) was found between survivors and non-survivors, but not in hemoglobin levels. Future studies should explore the impact of iron metabolism and anemia in the pathophysiology, prognosis, and treatment of COVID-19.

**Electronic supplementary material:**

The online version of this article (10.1007/s10654-020-00678-5) contains supplementary material, which is available to authorized users.

## Introduction

Infection with severe acute respiratory syndrome coronavirus 2 (SARS-COV-2) often results in Coronavirus disease 2019 (COVID-19), a disease that endangers disproportionately the elderly, those with pre-existing chronic conditions such as cardiovascular disease, diabetes mellitus and hypertension [1, 2]. If deteriorating, COVID-19 can lead to sepsis, septic shock, and multiple organ dysfunction syndrome, with mechanical ventilation or extracorporeal membrane oxygenation having low therapeutic efficacy [3]. The pathophysiological background underlying deterioration and low efficacy of common treatments is unclear.

Most patients with COVID-19 who require intensive care will develop an atypical form of the acute distress respiratory syndrome (ARDS) with preserved lung gas volume [4], suggesting hypoxia due to physiological processes other than alveolar dysfunction may play a role in the prognosis of the disease [5]. Disturbed iron metabolism may be one such affected process. Indeed, recent data show that COVID-19 patients tend to present decreased hemoglobin levels indicating the presence of anemia, and pathologically increased levels of ferritin. A study of 67 COVID-19 patients in Singapore reported that during their course in an intensive care unit (ICU), patients developed more profound and significantly lower hemoglobin levels, compared to patients not admitted to ICU [6]. Another study in elderly patients hospitalized for COVID-19 found that most patients had hemoglobin levels lower than the normal range, but did not find significant differences in hemoglobin levels between survivors and non-survivors. However, follow-up was incomplete for half of the patients [7]. In a report of 5700 patients hospitalized for COVID-19 in the New York City area, ferritin levels were pathologically high, a finding in line with previous studies from China [8, 9]. Both anemia and hyperferritinemia, regardless of the underlying pathology, are strong predictors of mortality [10, 11]. Anemia could be the result of iron-restricted erythropoiesis arising from alterations in iron metabolism. Increased ferritin levels could be indicative of a strong inflammatory reaction in COVID-19 or related to viral entry into the human body and its impact on iron metabolism [12, 13]. Iron is an essential micronutrient for both humans and pathogens [14]. The innate immune response could restrict iron availability during infections to deprive the pathogen of it, a mechanism that would also lead to anemia [15, 16]. Anemia, in turn, reduces oxygen delivery to the tissue and may thus play an important role in the development of multi-organ failure. Therefore, it is crucial to understand the relation between anemia, iron metabolism and progression of COVID-19, and whether these associations differ by age, sex and presence of chronic conditions.

We conducted a systematic review and meta-analysis of available observational evidence to (i) quantify the mean levels of hemoglobin, ferritin and other biomarkers of iron metabolism, and of biomarkers related to erythrocyte indices in COVID-19 patients, (ii) explore whether the levels would differ by age, sex presence of chronic conditions and severity of COVID-19, and (iii) whether these biomarkers could have clinical and/or prognostic utility in COVID-19.

## Methods

We conducted a systematic review and meta-analysis according to a recently published guideline and reported according to PRISMA (Preferred Reporting Items for Systematic Reviews and Meta-Analyses) guidelines [17]. The protocol was registered in PROSPERO (CRD42020180670) and is available at https://www.crd.york.ac.uk/prospero/display_record.php?RecordID=180670.

### Data source and strategy

We searched MEDLINE (National Library of Medicine, US), EMBASE (Elsevier, Netherlands), Web of Science (Clarivate Analytics, US), Cochrane (Cochrane Collaboration, UK), the WHO COVID-19 database and Google Scholar (Google, Inc., US) to identify relevant articles. We used search terms related to COVID-19 infection and SARS-CoV-2 virus, and several markers of anemia and iron storage and metabolism, including hemoglobin, ferritin, transferrin, soluble transferrin receptor, hepcidin, haptoglobin, unsaturated iron-binding capacity, erythropoietin, free erythrocyte protoporphyrin, red blood cell count (RBC), red cell distribution width (RDW), red cell volume (RCV), mean corpuscular volume (MCV), mean corpuscular hemoglobin (MCH), mean corpuscular hemoglobin concentration (MCHC), reticulocyte count, and reticulocyte index. We also included search terms related to clinical progression of COVID-19, such as prognosis, severity, ICU admission, mortality, risk factors, clinical features, clinical characteristics or predictors. We searched the databases from inception until August 3rd 2020. We limited our search to human studies and restricted our analysis to articles published in English. The detailed search strategies are presented in eAppendix 1.

### Study selection and eligibility criteria

All observational studies (e.g., cross-sectional, cohort, and case–control studies), except for case reports and case-series, were included. We included studies that reported on the levels of the biomarkers of iron metabolism, erythropoietin and erythrocyte indices, hemoglobin levels or the prevalence of anemia among COVID-19 patients, or their levels by the clinical outcome of patients with COVID-19. Outcomes of interest include disease severity, admission to intensive care unit, mechanical ventilation, and mortality across all age groups. Studies examining the association between the biomarkers and risk of COVID-19 complications (e.g., admission to intensive care unit, death) were also included. Studies conducted in pregnant women and individuals with cancer were excluded.

### Data extraction

Two independent reviewers screened the titles and abstracts according to the selection criteria. We recorded data on the author's name, digital object identifier, study location, study design, sample size, demographic and clinical characteristics, laboratory results, disease severity, and outcome in a data extraction form. The form was developed, piloted, and discussed within the review group before full data extraction. All laboratory values were extracted as reported and then converted to conventional units based on the US National Institute of Standards and Technology conversion factors. For studies reporting only median and ranges (interquartile range, range, and maximum-minimum values), we converted these values into mean and standard deviation [18].

### Risk of bias assessment

The quality of included studies was assessed by two authors independently using the Newcastle–Ottawa Scale for case–control, cross-sectional and cohort studies, as applicable [19]. A third author adjudicated in case consensus was not reached. The scale was developed for non-randomized and observational studies and assessed quality in three broad categories, namely, selection of study groups/participants, comparability of the study groups/participants, and the assessment of exposure/outcome of interest. Quality was assessed on a 10-point scale and classified as good quality (9–10 points), fair quality (6–8), and poor quality (< 6).

### Data synthesis and analysis

Based on the extracted data of each study, we computed pooled means and standard deviations for each biomarker and anemia prevalence for all COVID-19 patients, and weighted mean difference in levels of biomarkers between severe versus moderate cases, and survivors versus non-survivors. For the adult population, anemia was defined according to World Health Organization (WHO) guidelines (hemoglobin levels for males < 130 g/L and females < 120 g/L). We pooled mean difference by using a random-effects model. Heterogeneity was quantified using the I^2^ statistic [20]. Country of the study, age-group (pediatric vs. adult), the mean age of the study population, gender distribution, percentage of comorbidities, and proportion of patients admitted to ICU were pre-specified as characteristics for assessment of heterogeneity and were evaluated using stratified analyses and univariate random effects meta-regression. To decrease the risk of double-reporting of patients, we did sensitivity analyses by (i) randomly excluding 20% of the included studies in the meta-analysis and (ii) excluding studies from the Asia Pacific region. Publication bias were appraised using funnel plots and Egger's test for assessing asymmetry. We used STATA 15.1 (Statacorp, Texas, US, 2017) for all the analyses. All analyses were done using 2-tailed tests with a *p*-value < 0.05 considered statistically significant.

## Results

### Study identification and selection

**Fig. 1:**
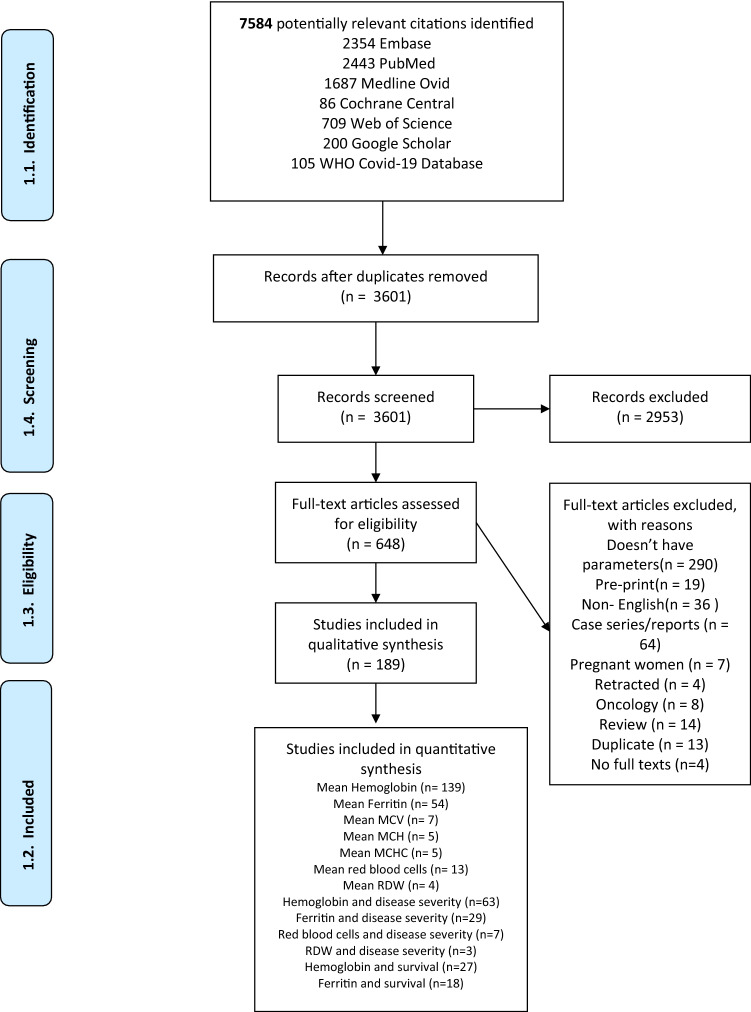
Flowchart of included studies

3,601 unique citations were identified, of which 648 were selected for full-text evaluation. Of those, under full-text evaluation, 189 observational studies comprising 57,563 COVID-19 patients were included for the final analysis. References of the included studies can be found in the Supplemental Material, and the flow chart of study selection results can be found in Fig. 1. In brief, the majority of the studies were conducted in the Asia Pacific region (n = 134, 72%), with 21, 20 and 14 studies coming from Europe, the US, and Middle-East, respectively. No study was found in South America. The majority of the studies were conducted in adults (n = 159, 84.13%), 22 studies (11.64%) included both pediatric and adult patients, and 8 studies (4.23%) included pediatric patients only. The mean age of study patients ranged from 2.5 to 75.25 years. The proportion of males ranged from 0 to 100%. Detailed characteristics of the included studies can be found in Supplemental Table S1.

### Serum hemoglobin levels

Due to heterogeneity among studies, we were not able to provide a pooled estimate on prevalence of anemia among Covid-19 patients. Based on findings from 139 observational studies comprising 40,450 individuals, pooled mean hemoglobin level was 129.7 g/L (95% Confidence Interval (CI) 128.51; 130.88; *I*^*2*^ = 98.2%, *P*-value for heterogeneity < 0.001, Table 1). In the subgroup analyses, mean hemoglobin levels were lower in studies including subjects above median age (55.25 years) and having a higher proportion of male participants, prevalent cardiovascular disease, diabetes and hypertension, and a higher proportion of ICU admission (Supplementary Table S2). Similarly, when fitting regression lines, significant linear trends were observed for mean hemoglobin levels decreasing with advancing mean age of study participants, increasing proportion of subjects with diabetes, hypertension and overall comorbidities in each study (Supplemental Figure S7). Compared to moderate COVID-19 cases (using data from 63 studies with 21,605 individuals), severe cases had lower hemoglobin levels [weighted mean difference (WMD), − 4.08 g/L (95% CI − 5.12; − 3.05); *I*^*2*^ = 59%, *P*-value for heterogeneity < 0.001] (Table 2, Supplemental Figure S1). The mean difference was larger in studies with a higher proportion of individuals with hypertension (Supplemental Table S3). In addition, we observed a lower mean difference in hemoglobin levels between severe and moderate COVID-19 cases with an increasing number of proportion of study participants with diabetes (Supplemental figures S8). Based on pooled estimates from 27 studies, we did not find a significant difference in hemoglobin levels between COVID-19 survivors and non-survivors [WMD, − 0.26 g/L (95% CI − 2.37; 1.85); *I*^*2*^ = 74%, *P*-value for heterogeneity < 0.001] (Table 2, Supplemental Figure S2). In subgroup analyses, none of the patient characteristics were identified as a source of heterogeneity (Supplemental Table S4). However, when fitting a regression line, the WMD of mean hemoglobin levels between survivors and non-survivors decreased with a higher percentage of diabetic patients in each study (Supplemental figures S9).

**Table 1:** Characteristics of studies included in meta-analysis of mean hemoglobin, ferritin, and other biomarkers levels and the main meta-analysis results

Outcome	Eligible studies	Participants			Location					Results of meta-analysis			
	Unique studies, no	Total	Median (IQR), no	Age, median (IQR), years	Europe	North America	South America	Asia–Pacific	Middle East	Overall mean (95% CI)	P value for pooled estimates	I^2^ for heterogeneity	P value for heterogeneity
Hemoglobin, g/L	139	40,450	124 (65–264)	53.75 (45–62)	12	9	–-	109	9	**129.7 ( 128.51; 130.88)**	< 0.001	98.2%	< 0.001
Ferritin, ng/mL	54	24,262	140 (69–263.5)	58.6 (51.94–63.65)	10	15	–-	23	6	**777.33 (701.89; 852.77)**	< 0.001	99.5%	< 0.001
Red blood cells, × 10^12^/L	13	1,382	99 (46–141.5)	57 (45.9–61.6)	2	–-	–-	11	–-	**4.09 ( 3.9; 4.28)**	< 0.001	97.2%	< 0.001
Red cell distribution width (RDW), %	4	1,538	220 (93.5–840)	55.5 (42.25–59.52)	–-	–-	–-	4	–-	**12.99 ( 12.62; 13.36)**	< 0.001	97.8%	< 0.001
Mean corpuscular volume (MCV), fL	7	2,054	208 (100–279)	52 (50–58.9)	––	––	––	6	1	**89.89 ( 88.05; 91.73)**	< 0.001	98.4%	< 0.001
Mean corpuscular hemoglobin (MCH), pg/cell	5	1,746	208 (116–653)	52 (44.5–59.35)	––	––	––	5	––	**30.68 ( 30.17; 31.18)**	< 0.001	96.0%	< 0.001
Mean corpuscular hemoglobin concentration (MCHC), g/dL	5	1,746	208 (116–653)	52 (44.5–59.35)	––	––	––	5	––	**338.05 (332.08; 344.03)**	< 0.001	99.6%	< 0.001

Statistically significant results are bold

**Table 2:** Meta-analysis of differences in mean hemoglobin, ferritin and other biomarkers levels based on disease severity and vital status

Biomarker	Eligible studies	Participants					Results of meta-analysis			
	Unique studies, no	Total	No. of deceased individuals	Mean biomarker level and 95% CI in deceased individuals	No. of survived individuals	Mean biomarker level and 95% CI in survived individuals	WMD (95%CI)	P value for pooled estimates	I2 for heterogeneity	P value for heterogeneity
Hemoglobin, g/L	27	9,125	2,366	125.55 (123.6; 127.51)	6,759	125.29 (122.65; 127.93)	− 0.26 (− 2.37; 1.85)	0.4	74.0%	< 0.001
Ferritin, ng/mL	18	7,190	1,707	1303.08 (1072.26; 1533.90)	5,483	650.67 (541.84; 759.51)	**606.37 ( 461.86; 750.88)**	< 0.001	90.9%	< 0.001
Biomarker	Unique studies, no	Total	No. of severe Covid-19 cases	Mean biomarker level and 95% CI in severe individuals	No. of Moderate Covid-19 cases	Mean biomarker level and SD in moderate cases	WMD (95%CI)	P value	I2 for heterogeneity	P value for heterogeneity
Red blood cells, × 10^12^/L	7	712	227	4.04 ( 3.8; 4.28)	485	4.21 ( 4.00; 4.42)	− **0.16 (**− **0.31;** − **0.01)**	0.001	43.8%	0.1
Red cell distribution width (RDW), %	3	421	97	6.39 (3.65; 9.13)	324	10.99 (10.15; 11.82)	**1.82 ( 0.10; 3.55)**	< 0.001	99.3%	< 0.001
Hemoglobin, g/L	63	21,605	8,241	127.19 (125.32; 129.08)	13,364	131.64 (129.25; 134.03)	− **4.08 (**− **5.12;** − **3.05)**	< 0.001	59%	< 0.001
Ferritin, ng/mL	29	13,620	5,511	1125.19 ( 976.15; 1274.22)	8,109	610.62 ( 520.56; 700.69)	**473.25 (382.52; 563.98)**	< 0.001	91.8%	< 0.001

Statistically significant results are bold

Among studies included in the narrative synthesis, Huang et al. [21] reported reduction in hemoglobin levels in 38.2% of hospitalized COVID-19 patients, but did not specify the definition of decreased hemoglobin. While Wang et al. [1] reported reduced hemoglobin levels (< 110 g/L) in 19.23% of the study population admitted to hospital. In contrast, Xu et al. [22] studied asymptomatic patients and reported none of the cases had decreased hemoglobin levels, albeit, not defining the cut-off of decreased levels. Based on retrospective data from 245 individuals with COVID-19, Liu et al. [23] found that the unadjusted association between baseline hemoglobin levels and all-cause mortality during hospitalization was non-significant, and the odds ratio of death with increasing serum hemoglobin level was 0.98 (95% CI 0.96, 1.00, *p* = 0.05). Cai et al. [24] studied factors associated with ICU admission and found no link between hemoglobin levels and odds of being admitted at ICU. In a study by Cen et al. [25] hemoglobin levels below 110 g/L were linked with disease progression in patients with COVID-19; univariable hazard ratio was 3.91 (95% CI 2.99–5.10). In addition, Giacomelli et al. [26] reported anemia (defined as hemoglobin levels below 125 g/L) was more prevalent in Covid-19 non-survivors (66.7%) compared to survivors (42.7%).

### Serum ferritin levels

Based on findings from 54 observational studies, including 24,262 COVID-19 patients, pooled mean ferritin level in COVID-19 patients was 777.33 ng/mL (95% CI 701.33; 852.77), *I*^*2*^ = 95.5%, *P*-value for heterogeneity < 0.001 (Table 1). In subgroup analyses, mean pooled ferritin levels were higher in studies including older population, higher proportion of hypertensive patients and of patients who did not survive (Supplementary Table S5). In addition, when regressing the most important study participants characteristic against mean serum ferritin levels, we observed significant linear trends with mean serum ferritin levels increasing with advancing age, increasing proportion of male study participants, number of hypertensive patients and proportion of patients admitted to ICU (Supplemental figures S10).

Based on pooled estimates from 29 studies and 13,620 individuals, the mean difference in serum ferritin was higher in severe COVID-19 individuals compared to moderate cases; [WMD, 473.25 ng/mL (95% CI 382.52; 563.98); *I*^*2*^ = 91.8%, *P*-value for heterogeneity < 0.001] (Table 2, Supplemental figure S3). Similarly, when pooling the estimates from 18 observational studies and 7,190 individuals we found higher mean ferritin levels in non-survivors as compared to survivors [ WMD, 606.37 ng/mL (95% CI 461.86; 750.88); *I*^*2*^ = 90.9%, *P*-value for heterogeneity < 0.001] (Table 2, Supplemental figure S4). In subgroup analyses concerning disease severity and survival, high heterogeneity between the studies was not explained by any of studied patient's characteristics. We observed a linear trend with WMD of ferritin levels between severe and moderate COVID-19 cases increasing with advancing age, while no linear trends were observed between patients characteristics and WMD in ferritin levels among survivors and non-survivors (Supplemental Table S6&7, Supplemental figure S11 & 12).

In line with our findings, Zhou et al. found in a univariable analysis that odds of in hospital death were higher among patients with ferritin levels above 300 ng/mL compared to those with serum ferritin ≤ 399 ng/mL( odds ratio was 9.10 (95% CI 2.04; 40.58, *p* = 0.0038). Indeed, levels of serum ferritin were elevated in non-survivors compared with survivors (562 ng/mL ± 492 ng/mL) throughout the clinical course, and were increased with disease deterioration [3]. Similarly, in a study by Li et al. [27] ferritin was significantly higher in severe cases. Shah et al. reported no significant differences in serum ferritin levels and transferrin saturation between patients with non-severe versus severe hypoxemia. Yet, in patients with severe hypoxemia they reported significantly lower levels of serum iron [median 2.3 μmol/L (IQR, 2.2–2.5)] in comparison to non-severe hypoxemia (median 4.3 μmol/L. (IQR, 3.3–5.2)] [28].

### Other biomarkers

We were able to pool the estimates from 13 and 4 studies on mean RBC and RDW comprising 1,382 and 1,538 subjects and the pooled mean RBC level and RDW were 4.09 × 10^12^/L (95% CI 3.9; 4,28) and 12.99% (95% CI 12.62;13.36) respectively. In addition, based on seven, five and three observational studies, pooled MCV, MCH and MCHC were 89.88 fL (95% CI 88.05; 91.75), 38.68 pg (95% CI 30.17;31.18) and 338.05 g/dL (95% CI 332.08; 344.03) respectively (Table 1, Supplemental Figure S5). Due to limited number of studies, we were able to perform meta-regression only for analyses concerning RBC. In subgroup analyses, mean RBC count was lower in studies including higher proportion of patients with comorbidities (73.2%); in line with this, with increasing proportion of subjects with comorbidities, RBC count was decreasing (Supplemental table S8 and Supplemental figure S13). When pooling the estimates from seven observational studies and 717 individuals with COVID-19, the mean difference in RBC count was lower in individuals with severe as compared to moderate disease [WMD, − 0.16 × 10^12^/ (95% CI − 0.31; − 0.014)], while RDW was higher [WMD, 1.82% (95% CI 0.10; 3.55] (Table 2, Supplemental Figure S6). We were not able to meta-analyze the other biomarkers of iron metabolism and anemia due to limited number of studies included in current review and/or high heterogeneity in reporting across the studies.

### Quality of studies and publication bias

The majority of studies included in the current systematic review were evaluated as low risk of bias (n = 114, 60.3%), while the rest of the studies were evaluated as medium risk of bias (n = 75) (Supplemental table S1 and S9). The funnel plot on ferritin difference between survivors and non-survivors was asymmetrical and Egger's P value was 0.002 suggesting the presence of publication bias, while for the rest of the meta-analyses including 5 or more studies we found no evidence of publication bias (Supplemental figures S14-16).

## Discussion

To the best of our knowledge, the current study is the first updated and comprehensive systematic review and meta-analyses acknowledging the potential clinical utility of anemia and iron metabolism in COVID-19. Based on data from 189 studies and 57,563 COVID-19 patients across all ages, we found a pooled mean hemoglobin level of 129.7 g/L, which decreased with older age and a higher proportion of comorbid illness and disease severity. We also found pathological values of ferritin in most patients, a finding more prominent in males, elderly and individuals with hypertension. Major differences in ferritin levels were reported between different levels of severity, and among patients who survived and those who did not. In addition, compared to moderate COVID-19 cases, severe patients had lower RBC count and higher RDW. Due to limited evidence and/or heterogeneity among included studies, we were not able to meta-analyze the evidence on other biomarkers of iron metabolism and anemia.

The mean level of hemoglobin in COVID-19 patients across all ages in this review represents a value for anemia diagnosis in men as defined by WHO (< 130 g/L), with anemic mean levels observed in the elderly, and for severe cases. Indeed, a study in 339 hospitalized elderly COVID-19 patients found that the majority of patients had mean values of hemoglobin lower than normal [7]. However, most of the studies did not report hemoglobin levels by sex, and none of the studies reported the prevalence of anemia based on age and sex-specific WHO cut-offs. Thus, it was challenging to provide an accurate picture of the burden of anemia in COVID-19 patients. The SASR-CoV-2 infection is associated with high mortality, especially among the elderly over the age of 65 + and comorbid patients.

Hemoglobin concentration is one of the most important determinants of the oxygen-carrying capacity of the blood. Low hemoglobin in COVID-19 patients, especially on populations at risk of complications and mortality, could indicate that the patients could suffer from a decreased capability of hemoglobin to support the increased peripheral tissue demands for oxygen due to the hyper-metabolic states during infection. Indeed, significant complications of COVID-19 patients are septic shock and multiple organ dysfunction syndrome, with mechanical ventilation or extracorporeal membrane oxygenation having low efficacy in mitigating its impact and progression [3]. While viral sepsis is often ignored in the clinical diagnosis, studies have shown COVID-19 patients to have developed sepsis, leading to subsequent multiple organ dysfunction and admission to ICU [29, 30]. According to recent evidence, COVID-19 patients do experience an atypical form of the acute distress respiratory syndrome with preserved lung gas volume [4], suggesting that the prognosis could depend on the ability of the human body to meet the oxygen demands of the peripheral tissues, which, if not met, may lead to hypoxia and ischemia. Studies have reported that anemia is associated with 2.6 times increased risk of mortality in chronic obstructive pulmonary diseases; the overall 90-day mortality among these patients with acute respiratory failure treated with invasive mechanical ventilation was 57.1% versus 25% in non-anemic patients [31, 32]. A previous meta-analysis has shown that among a mixed population, independent of sex, age and cardiovascular diseases, anemia is associated with a 41% and 33% increased risk of all-cause mortality and cardiovascular mortality, respectively [11]. A similar risk of mortality was also shown in two other meta-analyses comparing heart failure patients with and without anemia, or stroke patients with and without anemia [33, 34]. Also, another meta-analysis has shown that lower baseline hemoglobin values in heart failure patients are associated with increased crude mortality rates (*r* =  − 0.396, *p* = 0.025) [33]. In this meta-analysis we show that the severity of disease and prognosis of patients with COVID-19 might depend on lower hemoglobin levels as severe cases had significantly lower hemoglobin levels than moderate cases. Future prospective studies with complete follow-up are needed to confirm the impact of anemia in COVID-19 outcomes, and whether the incidence of low hemoglobin levels does predict mortality in these patients.

**Fig. 2:**
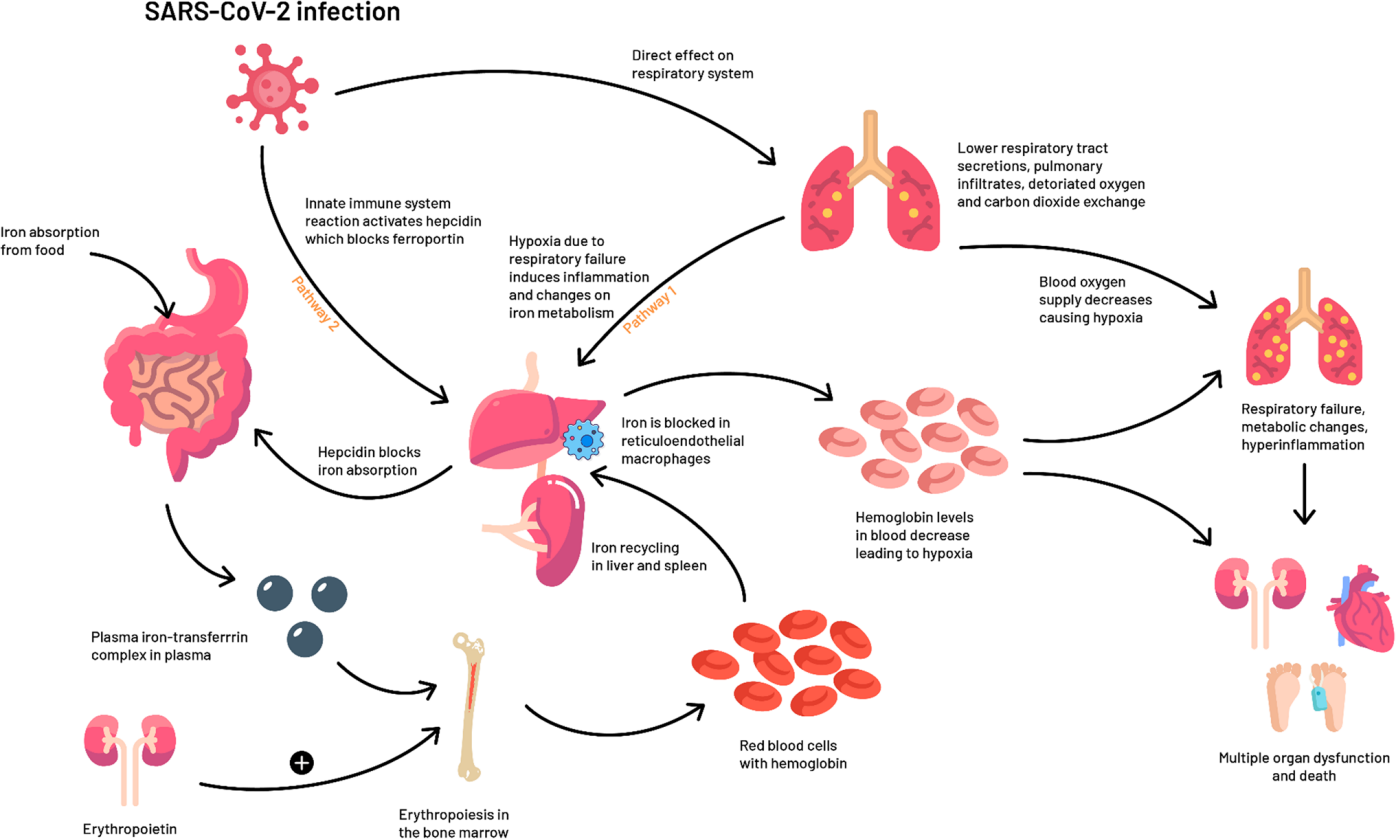
The potential role of red blood cell dynamics and iron homeostasis in the clinical presentation of COVID-19. The figure shows two potential pathways through which iron metabolism may be involved in the pathophysiology of COVID-19. Pathway 1: the virus inflicts hypoxia via direct deleterious effects on the respiratory system, altering the inflammatory response leading to anemia. Pathway 2: the innate immune system may aim to decrease the bioavailability of iron in order to prevent an expanding viral load in the acute-phase of the infection. This leads to the activation of hepcidin, sequestration of iron within cells, increased levels of ferritin and decreased hemoglobin, culminating in hypoxia

A striking finding of this meta-analysis is the pathological value of ferritin among COVID-19 patients, with significant differences between severe and moderate cases, and survivors and non-survivors. Ferritin is known to be elevated in inflammatory conditions, with hyperferritinemia being a key acute-phase reaction used by clinicians as a marker for therapeutic response [12]. However, recent studies suggest that increased levels of circulating ferritin levels may not only reflect an acute-phase response, but also play a critical role in inflammation by contributing to the development of a cytokine storm [12]. According to Shoenfeld et al. [35] the clinical picture of the critical cases of COVID-19 resembles macrophage activating syndrome, which is commonly associated with high levels of ferritin or even a cytokine storm. H-chain of the ferritin could be important in activating macrophages to increase the secretion of inflammatory cytokines observed in COVID-19 patients [35]. Another explanation for the increased levels of ferritin could be the role of iron metabolism in supporting the innate immune system to fight invading microorganisms. The innate immune system orchestrates control over iron metabolism as a response to viral infections. For viral replication, enhanced cellular metabolism and optimal iron levels within host cells are necessary [13, 36]. Therefore, the innate immune system will react by decreasing the bioavailability of iron to limit the replication of the virus during the acute phase of infection. In these conditions, through interleukin-6 and Toll-like-receptor-4 dependent pathways, the levels of the liver-derived iron hormone hepcidin-the master regulator of iron homeostasis- could increase and block, the activity of the transporter ferroportin which carries iron out of the cells, and therefore decrease the amount of iron absorbed from the diet, causing cellular sequestration of iron (i.e., principally in hepatocytes, enterocytes, and macrophages) [36]. Increased intracellular iron sequestration will lead to an upregulation of cytosolic ferritin, which sequesters and stores iron to prevent iron-mediated free radical damage [36]. The increased retention and storage of iron within ferritin in macrophages contribute to the characteristic fall in serum iron concentrations and an increase in serum ferritin concentrations as observed in an acute phase response [37]. The net result will be a diminished iron availability for erythropoiesis and as a result further aggravation of anemia. This is in line with our findings showing the worst erythrocyte phenotypes in severe COVID-19 patients compared to non-severe cases. Also, high ferritin levels and/or anemia could be secondary to baseline haemoglobinopathies such as alpha thalassemia, which is highly prevalent in Chinese population (up to 7.8%), from which most of our studies were derived [38, 39]. Regardless of the etiology (Fig. 2), serum ferritin is highly elevated in patients with COVID-19, and it seems warranted to assess whether serum ferritin could be used as a screening biomarker for the severity of the inflammatory state that patients with COVID-19 have.

Our systematic review highlights important gaps in the role of iron biomarkers other than ferritin in the prognosis of COVID-19. Of the included iron status parameters, only information on ferritin levels was available. Future studies should also measure other iron status parameters to establish whether iron-restricted erythropoiesis is inflicting anemia, and whether anemia is contributing to poor outcomes. Assessing the other stated parameters and interpreting them with respect to iron status in the setting of inflammation will be a challenging task. Therefore, the inclusion of soluble transferrin receptor (sTfR) could be of additive value for iron-restricted erythropoiesis, as elevated levels of sTfR reflect both erythroid activity and functional iron deficiency. The markers are known to be less affected by concomitant chronic disease or inflammation. Future prospective population-based and clinical studies are necessary to investigate the utility of using levels of hemoglobin levels and iron biomarkers for risk stratification and to identify patients who could benefit from early prevention strategies. Provided that anemia and altered iron metabolism play a role in COVID-19, public health strategies may be designed to protect this population that could be at risk of COVID-19 complications.

This study has several limitations. First, considering we restricted our review to articles in English, we cannot rule out publication bias, which could limit our overall findings. Second, the interpretation of the findings should be based on the quality of the included studies. Despite most studies being of high quality, the data provided are mainly of cross-sectional nature. Third, there was heterogeneity in the definition of moderate and severe cases of COVID-19 patients and in the definition of comorbid patients, which could have contributed to the observed heterogeneity in our meta-analysis. Finally, we cannot exclude the possibility that some study participants may overlap across the included studies. However, when randomly excluding 20% of studies from our analyses, or restricting the analyses to studies outside the Asia Pacific region, the results remained in line with the overall findings (Supplementary Tables S3-7). Also, some studies did not have a complete follow-up, and therefore our stratified analyses by the percentage of survivors should be interpreted with caution.

This meta-analysis suggests that hemoglobin and ferritin levels vary according to the severity of COVID-19 as well as age, gender and presence of comorbidity among COVID-19 patients. Whether hemoglobin and ferritin can be used for prognostic purposes, or have further implications for identifying novel treatment targets, needs further investigation.

## Electronic supplementary material

Below is the link to the electronic supplementary material.

Supplementary file1 (DOCX 3444 kb)

## References

[1] Wang L, Duan Y, Zhang W, Liang J, Xu J, Zhang Y (2020). Epidemiologic and clinical characteristics of 26 cases of Covid-19 arising from patient-to-patient transmission in Liaocheng. China Clin Epidemiol.

[2] Yang J, Zheng Y, Gou X, Pu K, Chen Z, Guo Q (2020). Prevalence of comorbidities and its effects in coronavirus disease 2019 patients: a systematic review and meta-analysis. Int J Infect Dis.

[3] Zhou F, Yu T, Du R, Fan G, Liu Y, Liu Z (2020). Clinical course and risk factors for mortality of adult inpatients with COVID-19 in Wuhan, China: a retrospective cohort study. Lancet.

[4] Gattinoni L, Coppola S, Cressoni M, Busana M, Rossi S, Chiumello D (2020). Covid-19 Does Not Lead to a "Typical" Acute Respiratory Distress Syndrome. Am J Respir Crit Care Med..

[5] Lang M, Som A, Mendoza DP, Flores EJ, Reid N, Carey D, et al. Hypoxaemia related to COVID-19: vascular and perfusion abnormalities on dual-energy CT. Lancet Infect Dis. 2020 (in press). 10.1016/S1473-3099(20)30367-4 PMC7252023 32359410

[6] Fan BE, Chong VCL, Chan SSW, Lim GH, Lim KGE, Tan GB (2020). Hematologic parameters in patients with COVID-19 infection. Am J Hematol..

[7] Wang L, He W, Yu X, Hu D, Bao M, Liu H (2020). Coronavirus disease 2019 in elderly patients: Characteristics and prognostic factors based on 4-week follow-up. J Infect..

[8] Richardson S, Hirsch JS, Narasimhan M, Crawford JM, McGinn T, Davidson KW, et al. Presenting characteristics, comorbidities, and outcomes among 5700 patients hospitalized with COVID-19 in the New York City Area. JAMA. 2020 (in press). 10.1001/jama.2020.6775 PMC7177629 32320003

[9] Chen T, Wu D, Chen H, Yan W, Yang D, Chen G (2020). Clinical characteristics of 113 deceased patients with coronavirus disease 2019: retrospective study. BMJ.

[10] Bennett TD, Hayward KN, Farris RW, Ringold S, Wallace CA, Brogan TV (2011). Very high serum ferritin levels are associated with increased mortality and critical care in pediatric patients. Pediatr Crit Care Med.

[11] Liu Z, Sun R, Li J, Cheng W, Li L (2019). Relations of anemia with the all-cause mortality and cardiovascular mortality in general population: a meta-analysis. Am J Med Sci.

[12] Kernan KF, Carcillo JA (2017). Hyperferritinemia and inflammation. Int Immunol.

[13] Wessling-Resnick M (2018). Crossing the iron gate: why and how transferrin receptors mediate viral entry. Annu Rev Nutr.

[14] Cassat James E, Skaar EP (2013). Iron in infection and immunity. Cell Host Microbe.

[15] Wessling-Resnick M (2018). Crossing the iron gate: why and how transferrin receptors mediate viral entry. Annu Rev Nutr.

[16] Ganz T (2019). Anemia of inflammation. N Engl J Med.

[17] Muka T, Glisic M, Milic J, Verhoog S, Bohlius J, Bramer W (2020). A 24-step guide on how to design, conduct, and successfully publish a systematic review and meta-analysis in medical research. Eur J Epidemiol.

[18] Hozo SP, Djulbegovic B, Hozo I (2005). Estimating the mean and variance from the median, range, and the size of a sample. BMC Med Res Methodol.

[19] Stang A (2010). Critical evaluation of the Newcastle-Ottawa scale for the assessment of the quality of nonrandomized studies in meta-analyses. Eur J Epidemiol.

[20] Higgins JP, Thompson SG, Deeks JJ, Altman DG (2003). Measuring inconsistency in meta-analyses. BMJ.

[21] Huang Y, Tu M, Wang S, Chen S, Zhou W, Chen D (2020). Clinical characteristics of laboratory confirmed positive cases of SARS-CoV-2 infection in Wuhan, China: A retrospective single center analysis. Travel Med Infect Dis..

[22] Xu T, Huang R, Zhu L, Wang J, Cheng J, Zhang B, et al. Epidemiological and clinical features of asymptomatic patients with SARS-CoV-2 infection. J Med Virol. 2020. 10.1002/jmv.25944. 10.1002/jmv.25944 PMC7267538 32346873

[23] Liu Y, Du X, Chen J, Jin Y, Peng L, Wang HHX (2020). Neutrophil-to-lymphocyte ratio as an independent risk factor for mortality in hospitalized patients with COVID-19. J Infect..

[24] Cai SH, Liao W, Chen SW, Liu LL, Liu SY, Zheng ZD (2020). Association between obesity and clinical prognosis in patients infected with SARS-CoV-2. Infect Dis Poverty.

[25] Cen Y, Chen X, Shen Y (2020). Risk factors for disease progression in patients with mild to moderate coronavirus disease 2019-a multi-centre observational study. Clin Microbiol Infect.

[26] Giacomelli A, Ridolfo AL, Milazzo L (2020). 30-day mortality in patients hospitalized with COVID-19 during the first wave of the Italian epidemic: a prospective cohort study. Pharmacol Res.

[27] Li X, Xu S, Yu M, Wang K, Tao Y, Zhou Y (2020). Risk factors for severity and mortality in adult COVID-19 inpatients in Wuhan. J Allergy Clin Immunol..

[28] Shah A, Frost JN, Aaron L, Donovan K, Drakesmith H (2020). Collaborators Systemic hypoferremia and severity of hypoxemic respiratory failure in COVID-19. Crit Care.

[29] Liu D, Wang Q, Zhang H, Cui L, Shen F, Chen Y, Sun J, Gan L, Sun J, Wang J, Zhang J, Cai Q, Deng J, Jiang J, Zeng L. Viral sepsis is a complication in patients with Novel Corona Virus Disease (COVID-19). Medicine in Drug Discovery. In Press, 10.1016/j.medidd.2020.100057. 10.1016/j.medidd.2020.100057 PMC7378467 32838292

[30] Lin GL, McGinley JP, Drysdale SB, Pollard AJ (2018). Epidemiology and immune pathogenesis of viral sepsis. Front Immunol.

[31] Lomholt FK, Laulund AS, Bjarnason NH, Jorgensen HL, Godtfredsen NS (2014). Meta-analysis of routine blood tests as predictors of mortality in COPD. Eur Clin Respir J.

[32] Rasmussen L, Christensen S, Lenler-Petersen P, Johnsen SP (2010). Anemia and 90-day mortality in COPD patients requiring invasive mechanical ventilation. Clin Epidemiol.

[33] Groenveld HF, Januzzi JL, Damman K, van Wijngaarden J, Hillege HL, van Veldhuisen DJ (2008). Anemia and mortality in heart failure patients a systematic review and meta-analysis. J Am Coll Cardiol.

[34] Li Z, Zhou T, Li Y, Chen P, Chen L (2016). Anemia increases the mortality risk in patients with stroke: a meta-analysis of cohort studies. Sci Rep.

[35] Shoenfeld Y (2020). Corona (COVID-19) time musings: our involvement in COVID-19 pathogenesis, diagnosis, treatment and vaccine planning. Autoimmun Rev..

[36] Drakesmith H, Prentice A (2008). Viral infection and iron metabolism. Nat Rev Microbiol.

[37] Parrow NL, Fleming RE, Minnick MF (2013). Sequestration and scavenging of iron in infection. Infect Immun.

[38] Lai K, Huang G, Su L, He Y (2017). The prevalence of thalassemia in mainland China: evidence from epidemiological surveys. Sci Rep.

[39] Ghosh K, Colah R, Manglani M (2014). Guidelines for screening, diagnosis and management of hemoglobinopathies. Indian J Hum Genet.

