# Supplementary material for: Anemia and iron metabolism in COVID-19: a systematic review and meta-analysis
Source: Eur J Epidemiol. 2020 Aug 20;35(8):763–73. doi: 10.1007/s10654-020-00678-5 (PMC7438401; doi:10.1007/s10654-020-00678-5)
Supplement: Supplementary file 1 — Supplementary file1 (DOCX 3444 kb) [file 10654_2020_678_MOESM1_ESM.docx]

**Online Supplemental Material**

**Biomarkers levels of anemia and iron metabolism in Coronavirus disease 2019: A systematic review and meta-analysis**

**Supplemental Figure S1.** Meta-analysis of differences in mean hemoglobin levels between severe and moderate COVID-19 patients

**
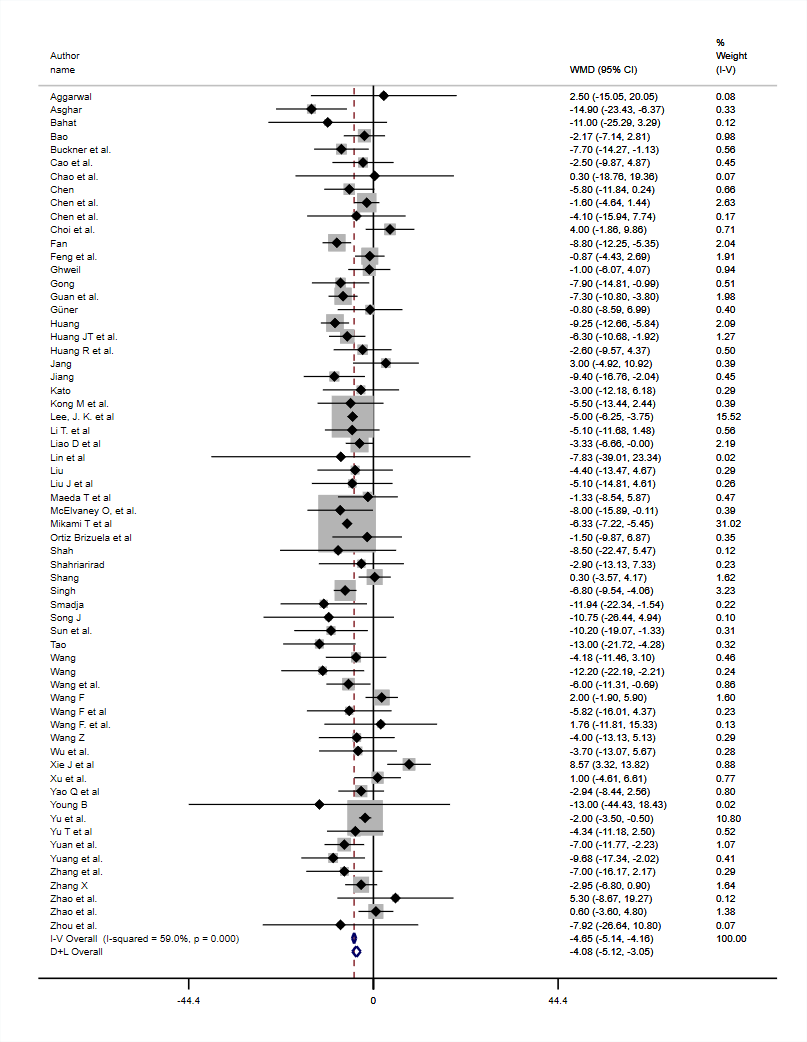
**

**Supplemental Figure S2.** Meta-analysis of differences in mean hemoglobin levels between survivors and non-survivors

**
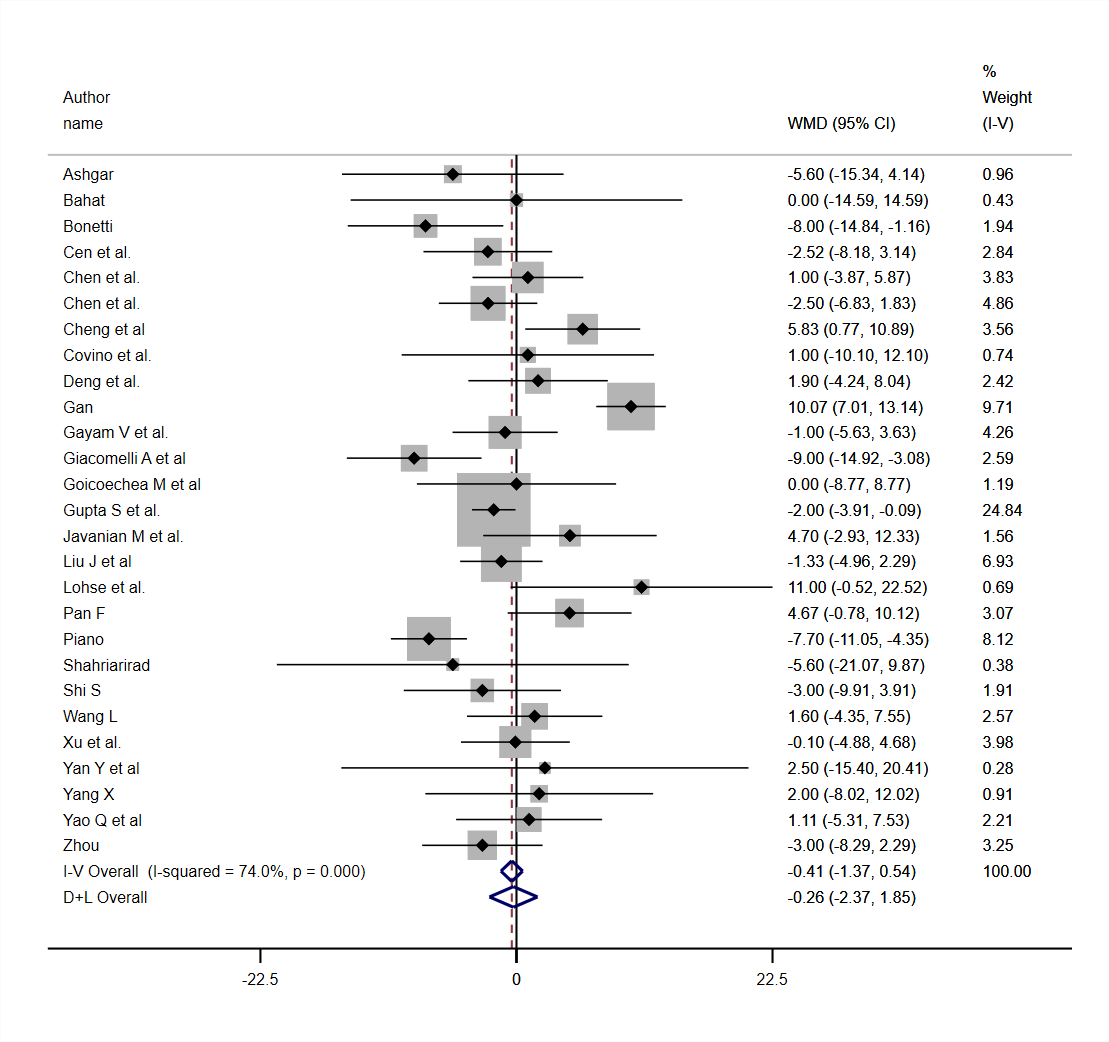
**

**Supplemental Figure S3.** Meta-analysis of differences in mean ferritin levels between severe and moderate COVID-19 patients

**
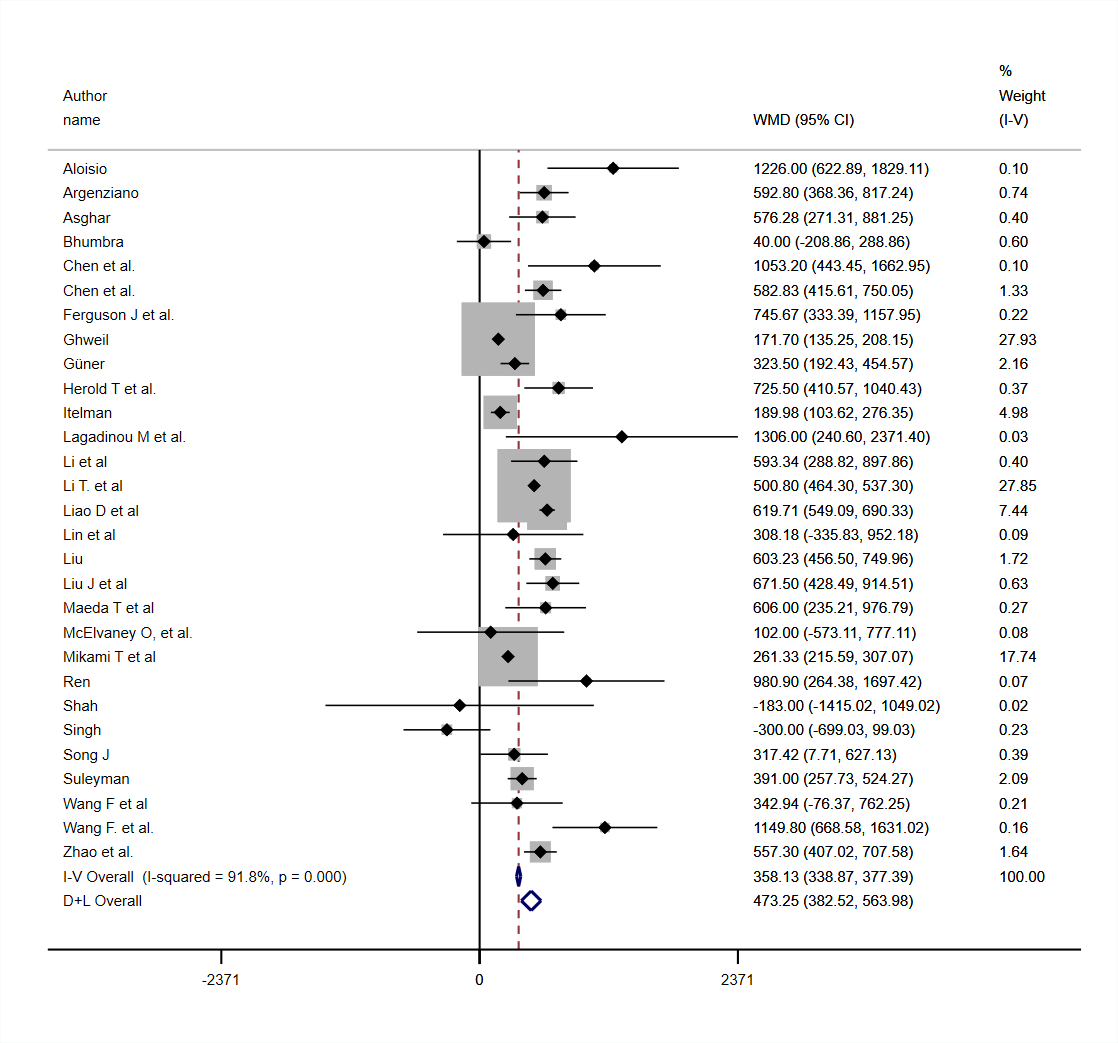
**

**Supplemental Figure S4.** Meta-analysis of differences in mean ferritin levels between survivors and non-survivors

**
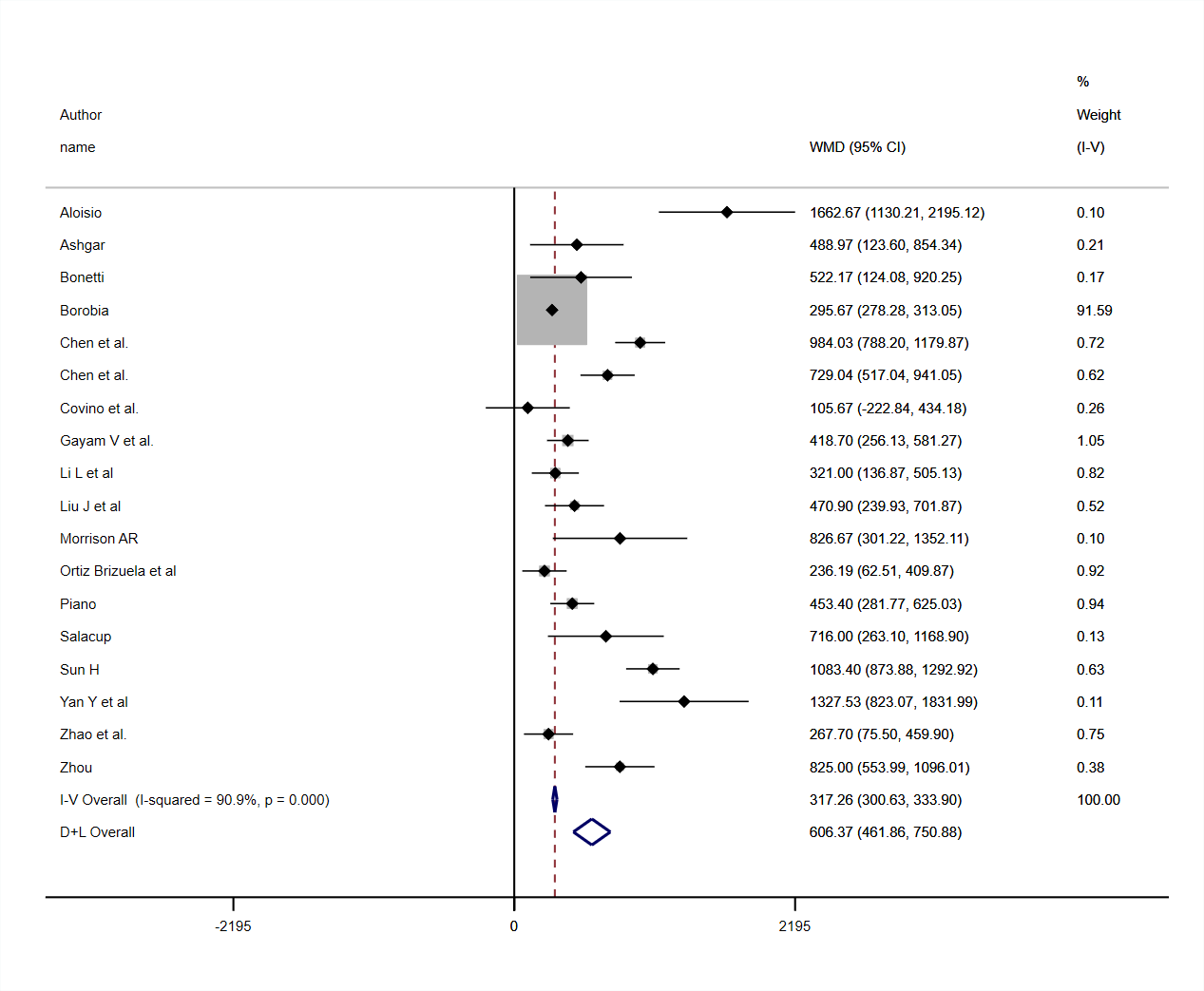
**

**Supplemental Figure S5.** Meta-analysis of mean other biomarker levels in COVID-19 patients

**
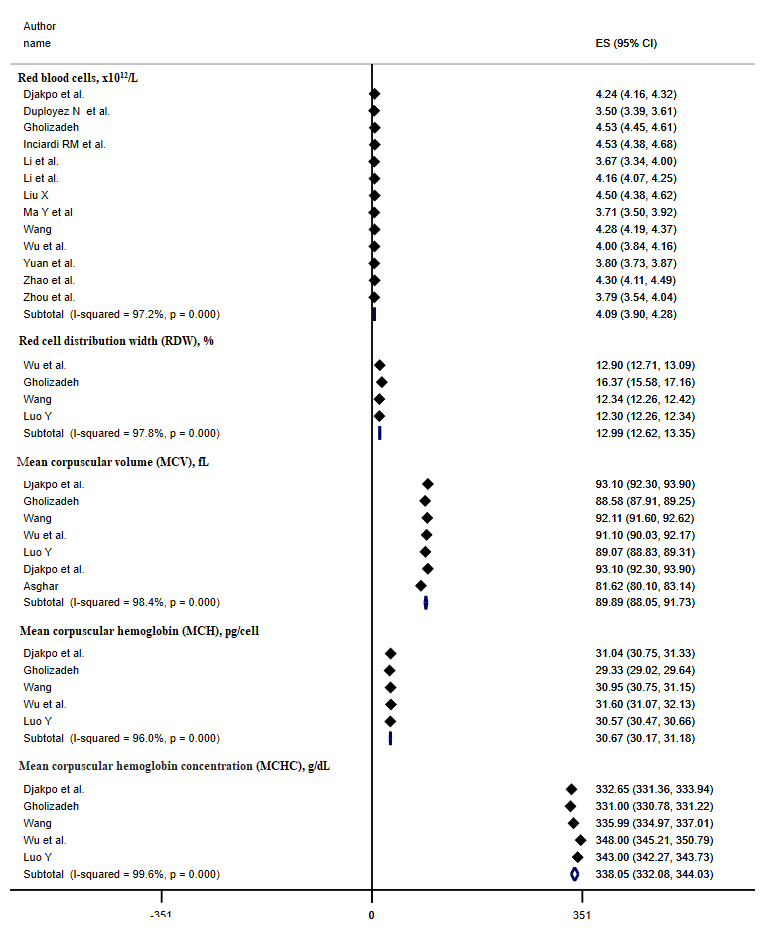
**

**Supplemental Figure S6.** Meta-analysis of differences in mean red blood cell count and red cell distribution width between severe and moderate COVID-19 patients

**
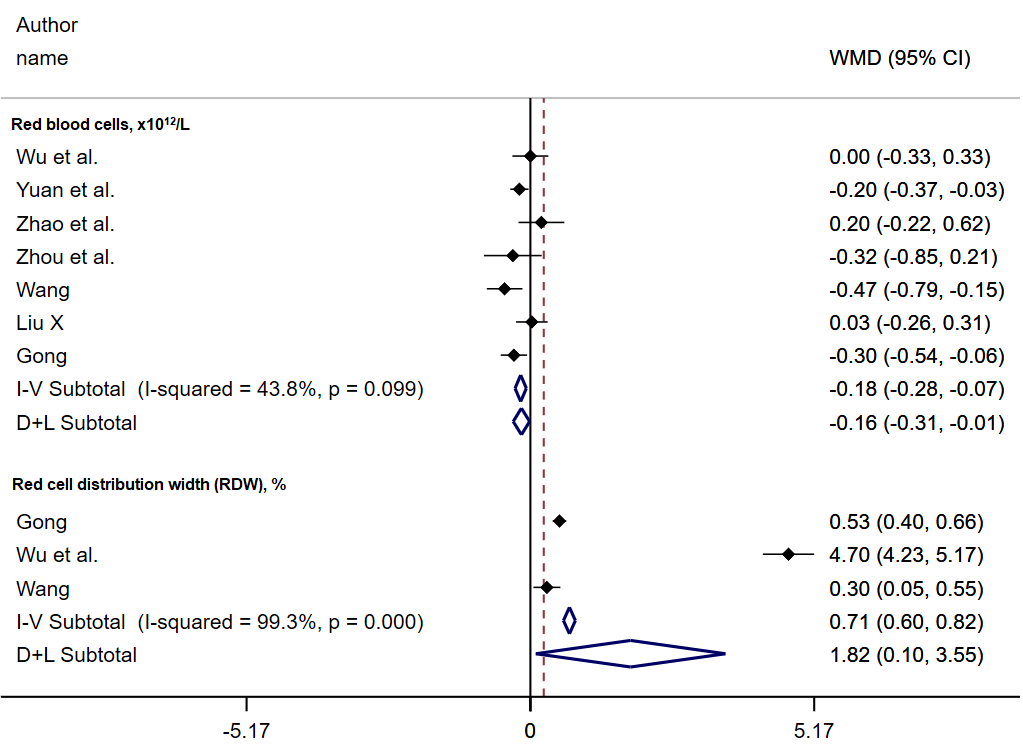
**

**Supplemental figures S7.** Bubble plots on mean hemoglobin levels by age, percentage of patients survived, percentage of male population, percentage of patients at intensive care and percentage of patients with comorbidities, perentage of patients with cardiovascular diseases, diabetes and hypertension

a11

**
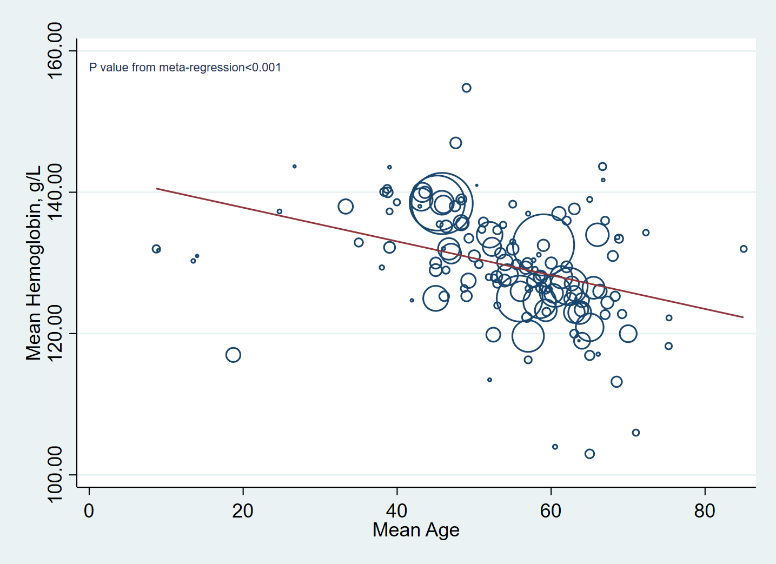

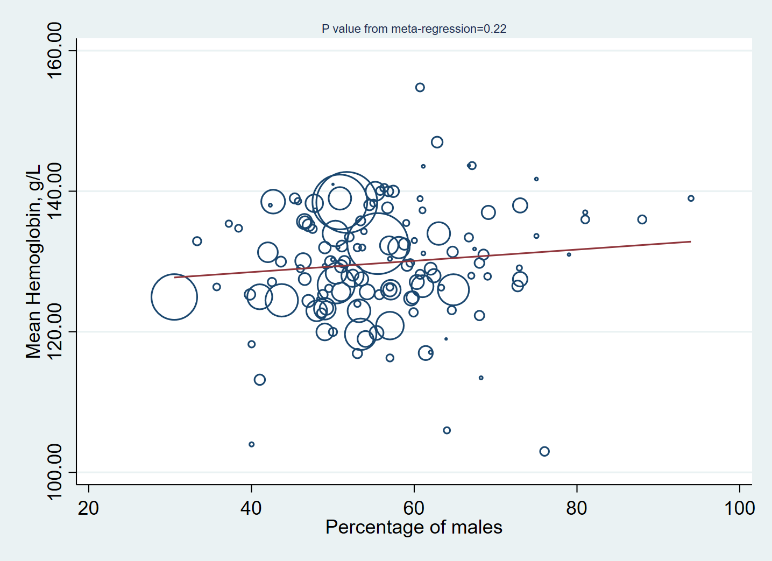
**

**
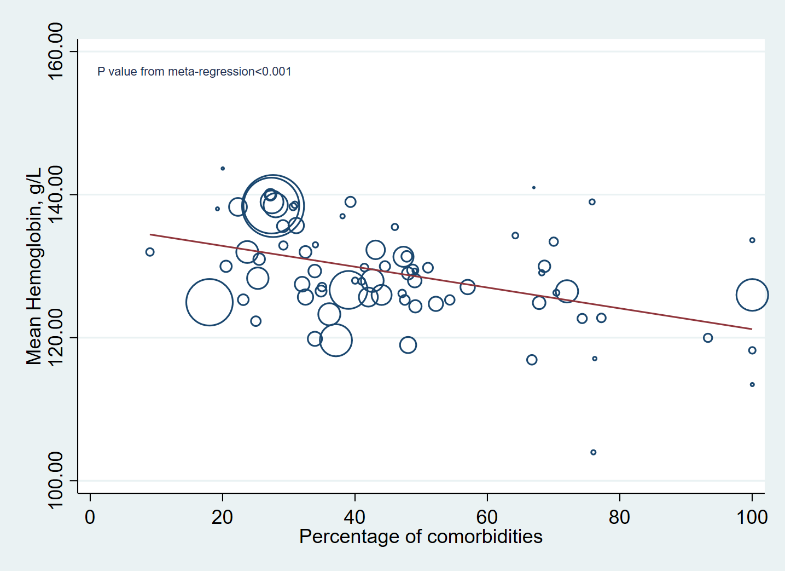
** **
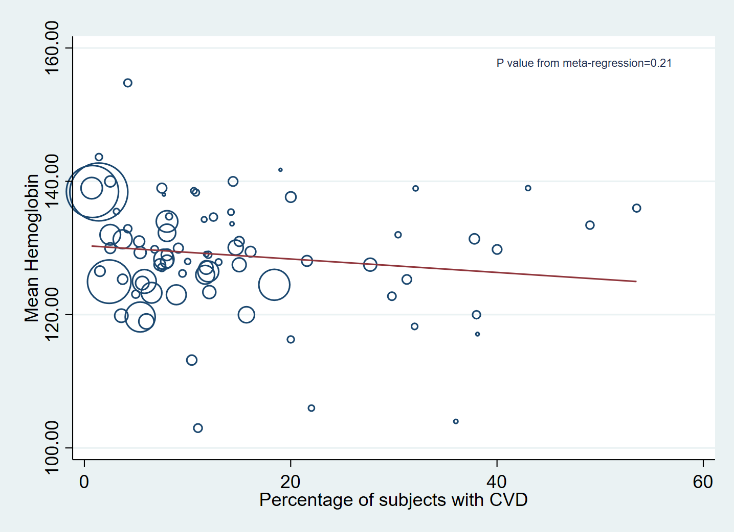

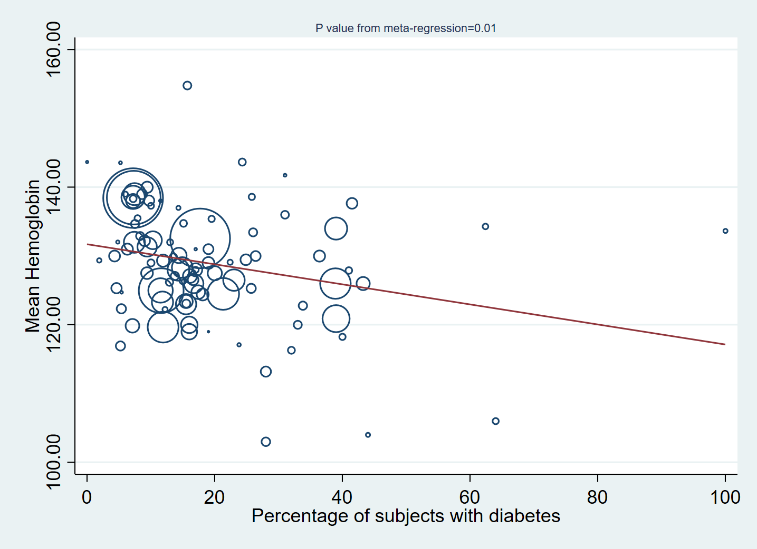

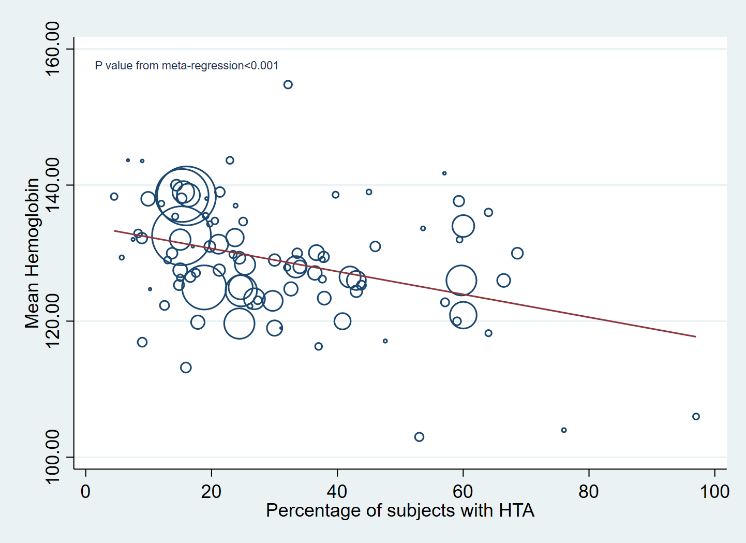
**

**
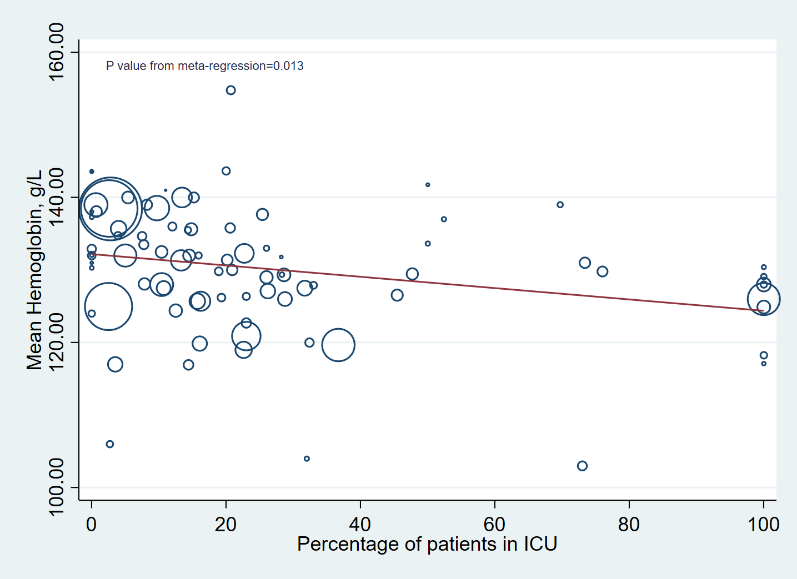

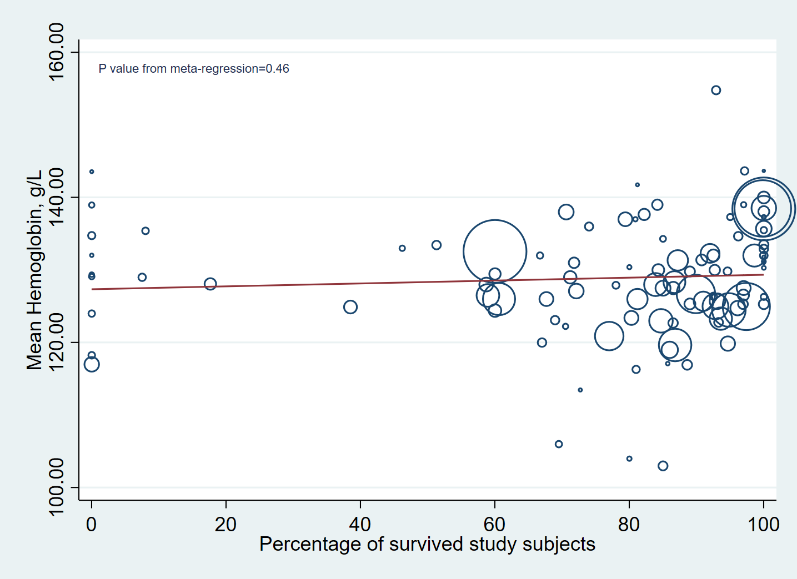
**

Circles are sized according to the precision of each estimate with larger bubbles for more precise estimates.

**Supplemental figures S8.** Bubble plots on weightened mean difference in hemoglobin levels between severe vs moderate Covid-19 cases by age, percentage of patients survived, percentage of male population, percentage of patients at intensive care and percentage of patients with comorbidities, perentage of patients with cardiovascular diseases, diabetes and hypertension

**
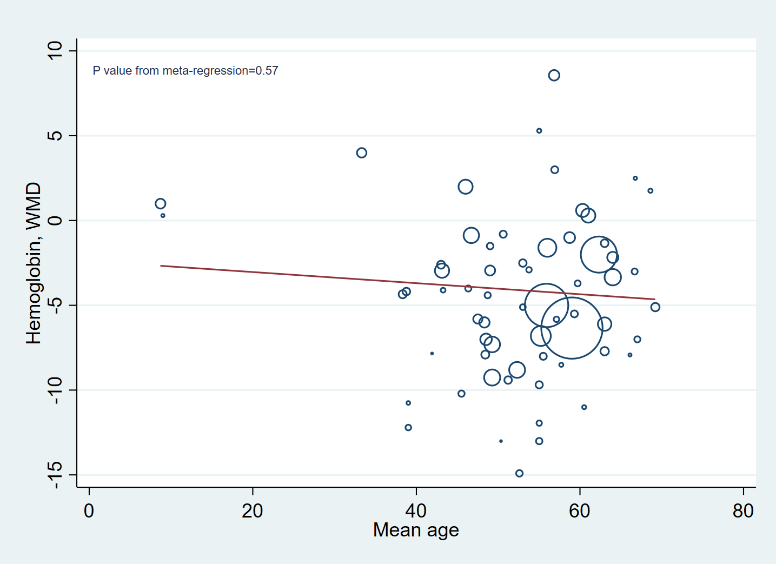

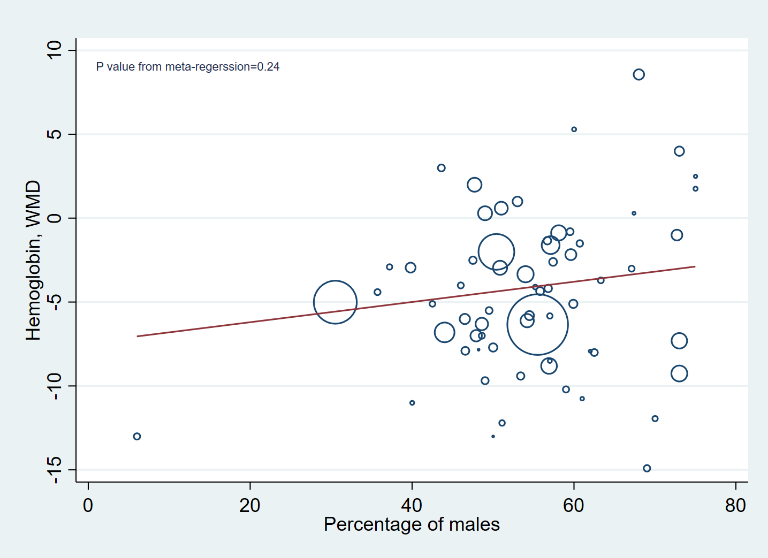

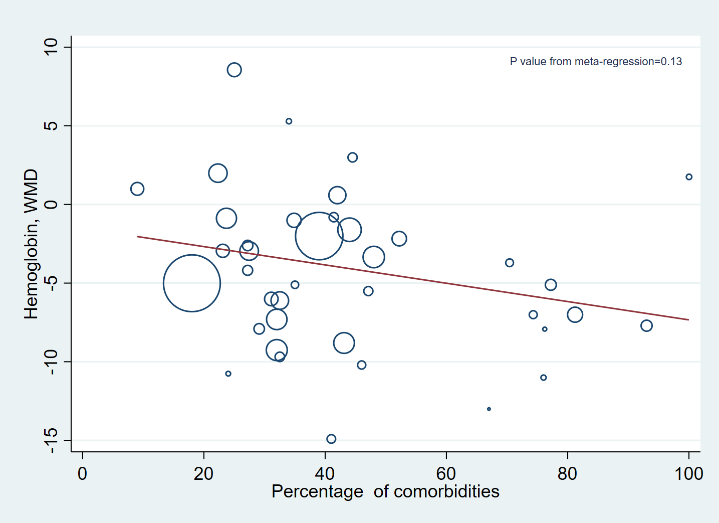

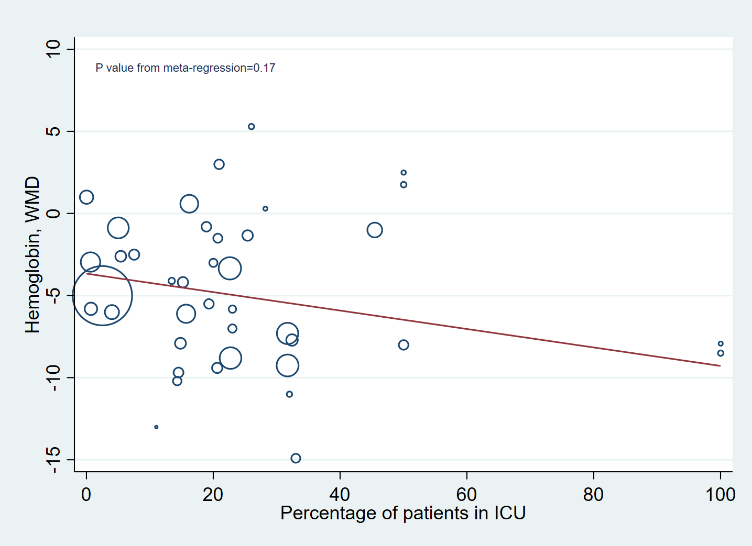
**


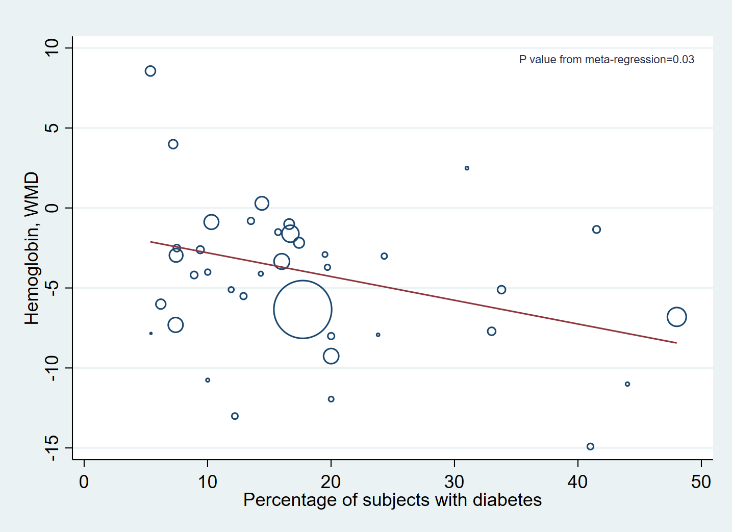

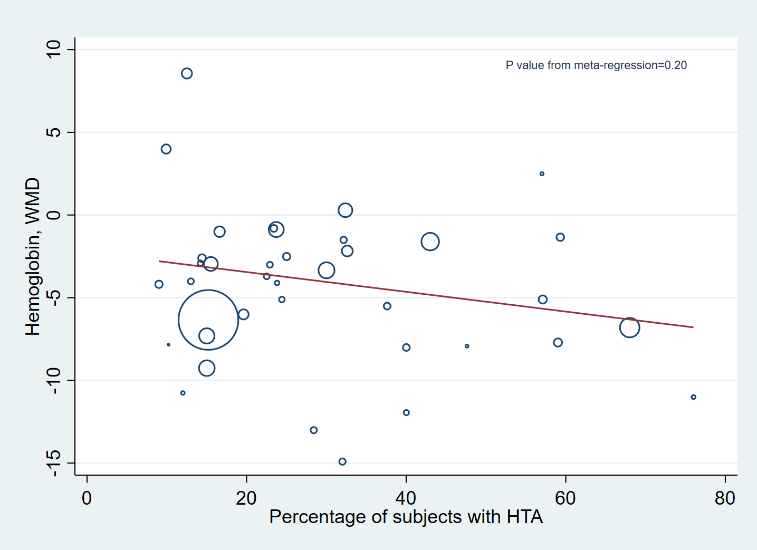


Circles are sized according to the precision of each estimate with larger bubbles for more precise estimates.


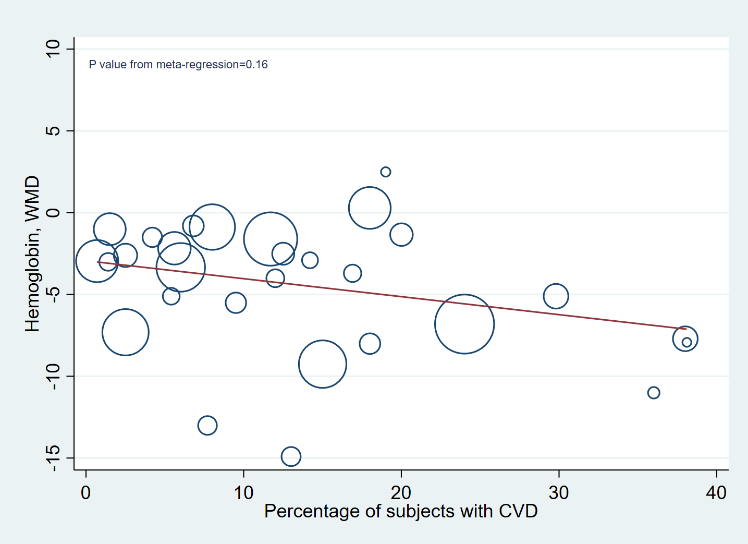
 **
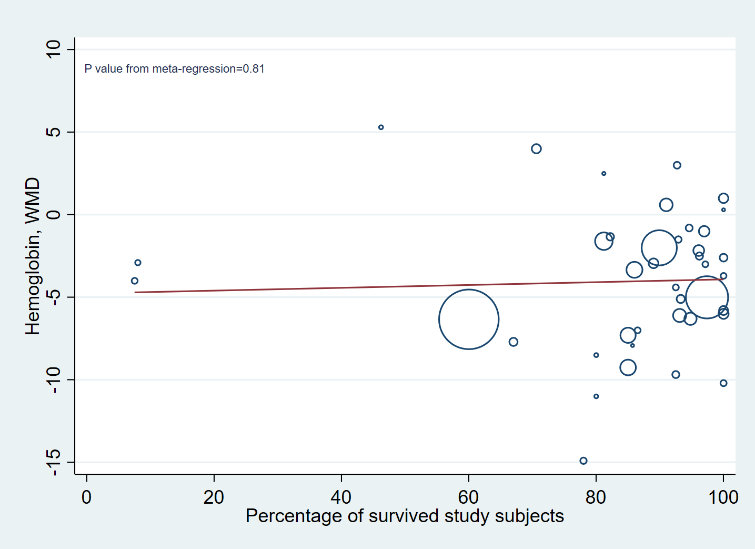
**

**Supplemental figures S9.** Bubble plots on weightened mean difference in hemoglobin levels between deceased vs survived Covid-19 cases by age, percentage of patients survived, percentage of male population, percentage of patients at intensive care and percentage of patients with comorbidities

**
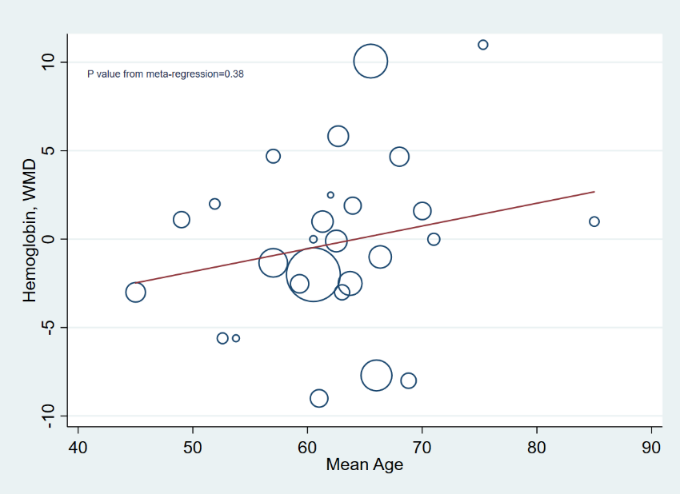

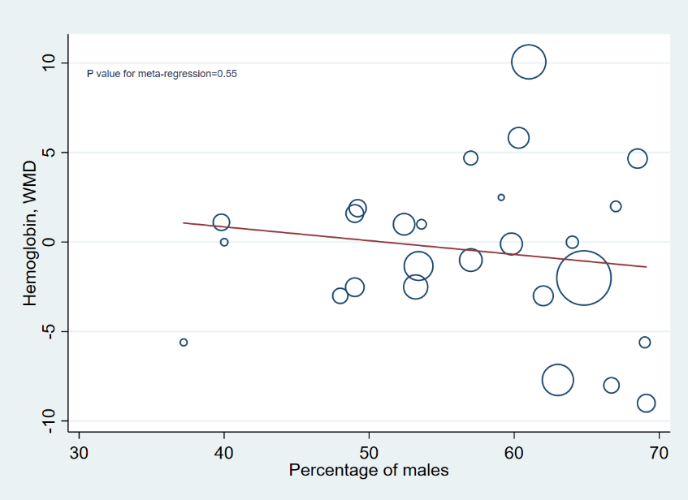

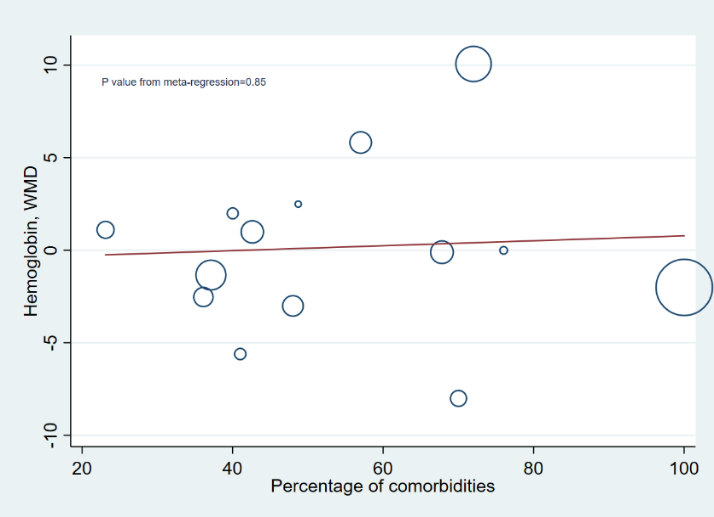

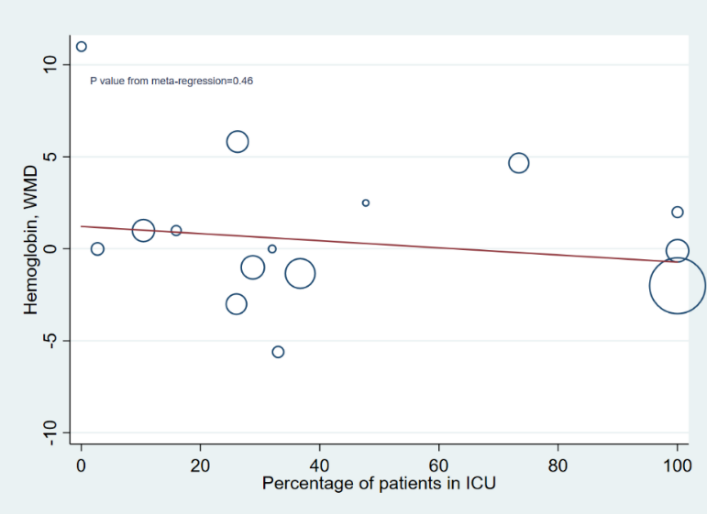

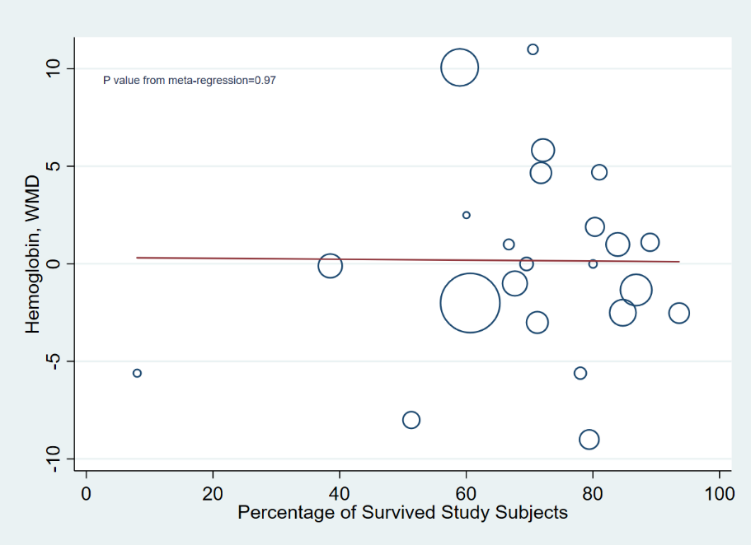

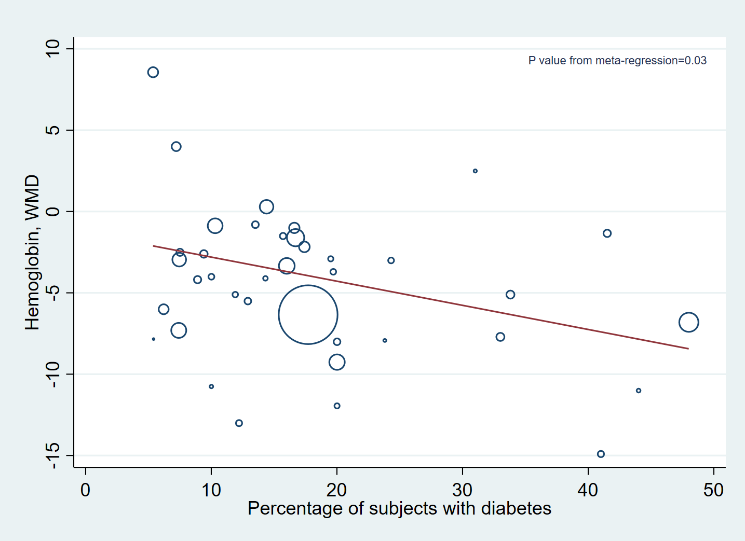

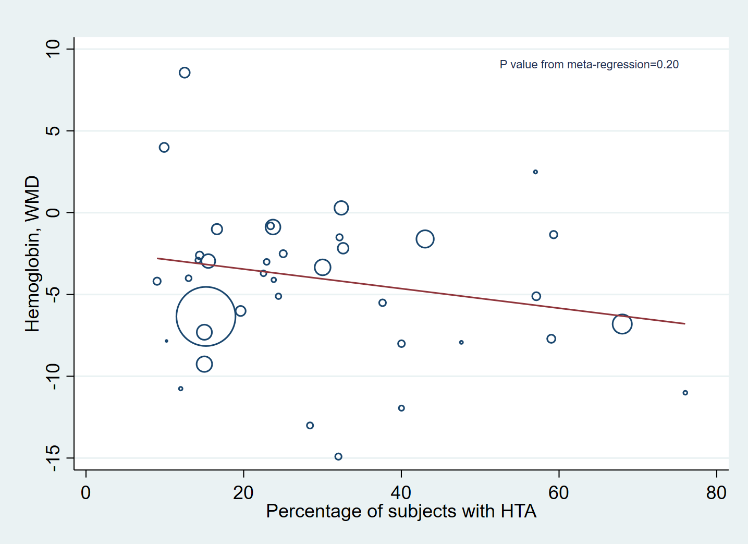

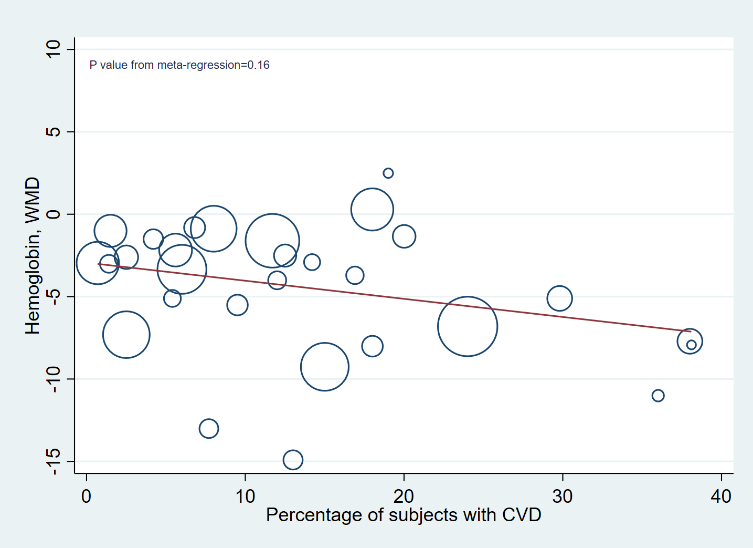
**

**Supplemental figures S10**. Bubble plots on mean ferritin levels by age, percentage of patients survived, percentage of male population, percentage of patients at intensive care and percentage of patients with comorbidities, perentage of patients with cardiovascular diseases, diabetes and hypertension and mean hemoglobin levels


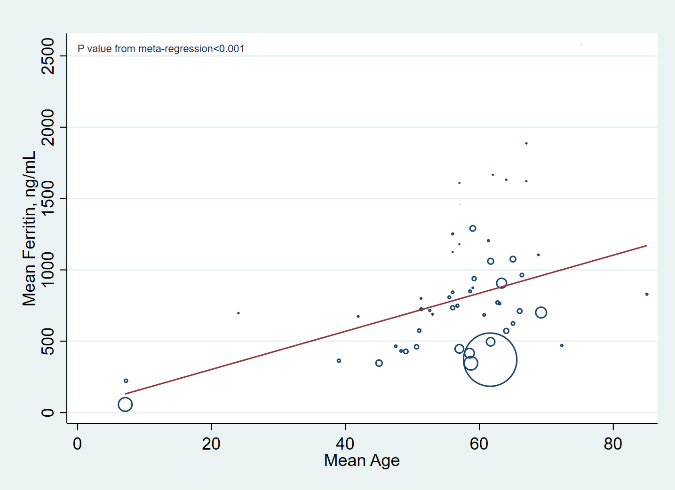

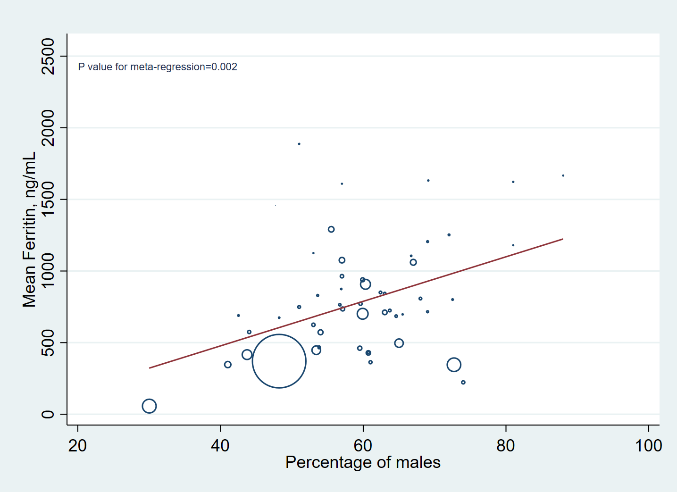


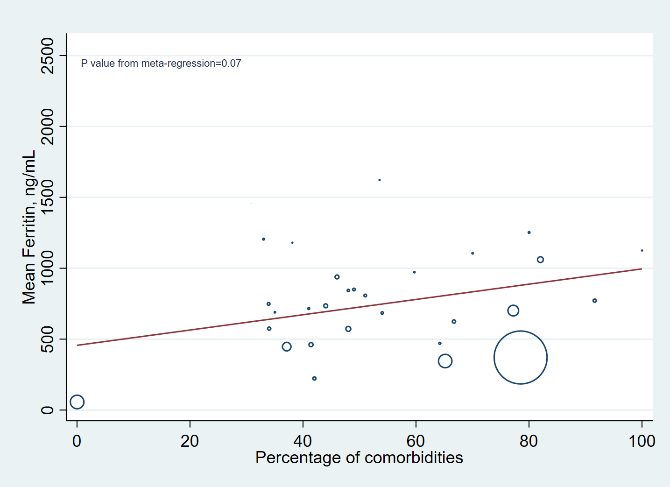

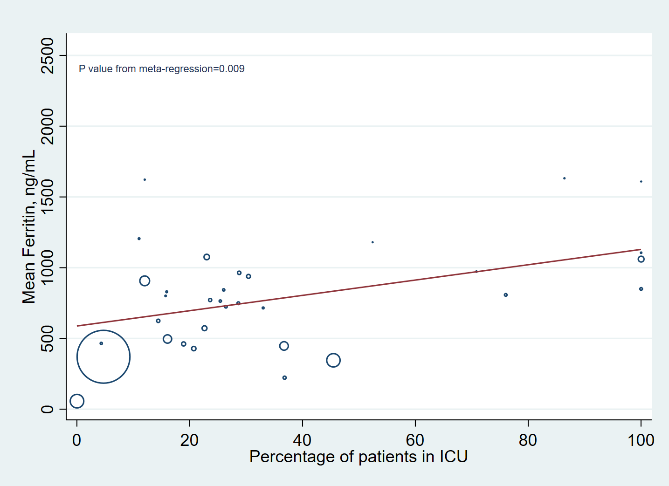


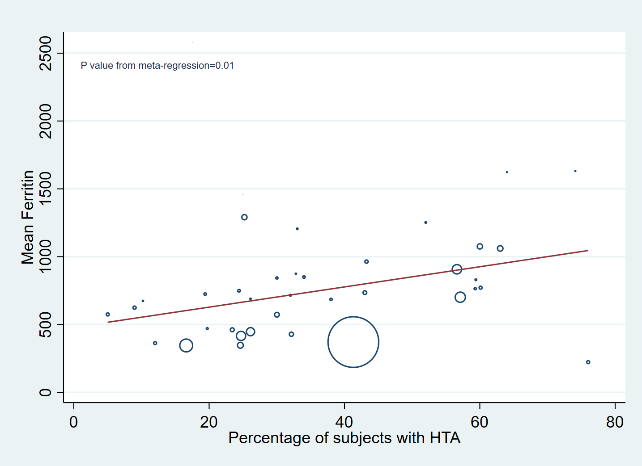

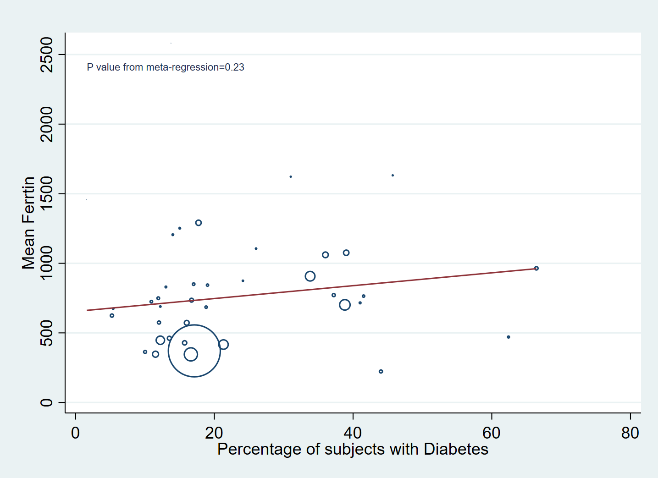

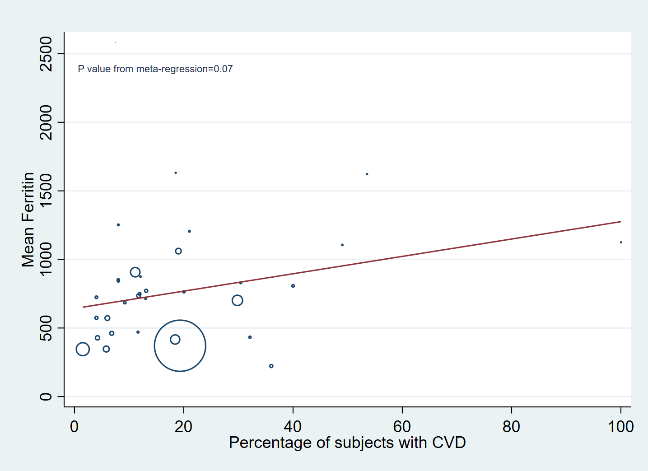

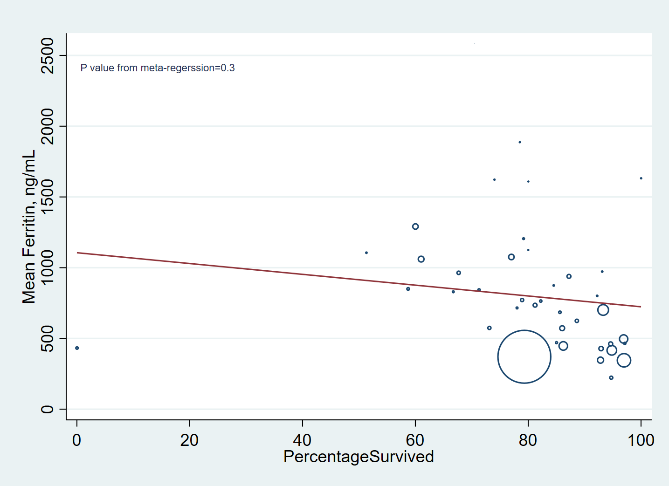

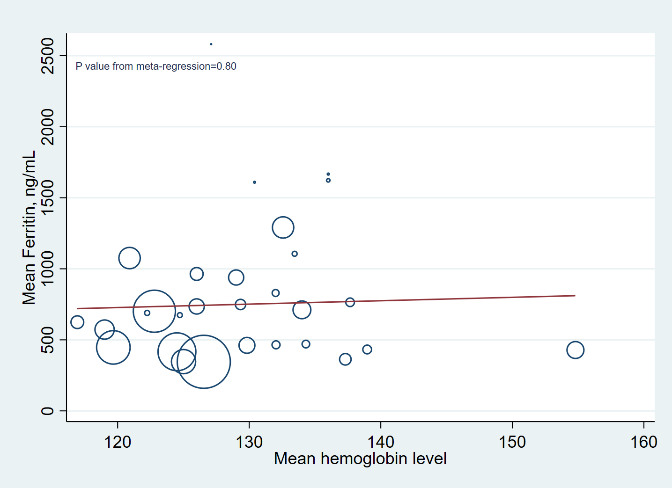


**Supplemental figures S11.** Bubble plots on weightened mean difference in ferritin levels between severe vs moderate Covid-19 cases by age, percentage of patients survived, percentage of male population, percentage of patients at intensive care and percentage of patients with comorbidities, perentage of patients with cardiovascular diseases, diabetes and hypertension

**
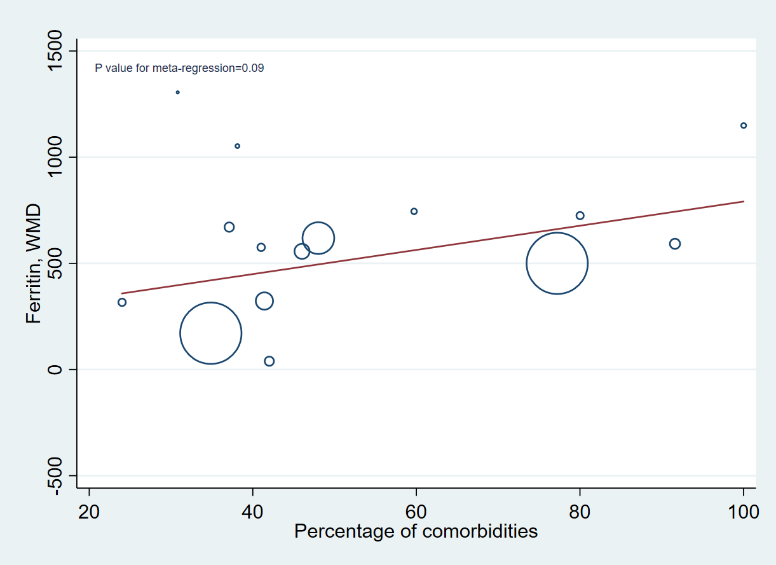

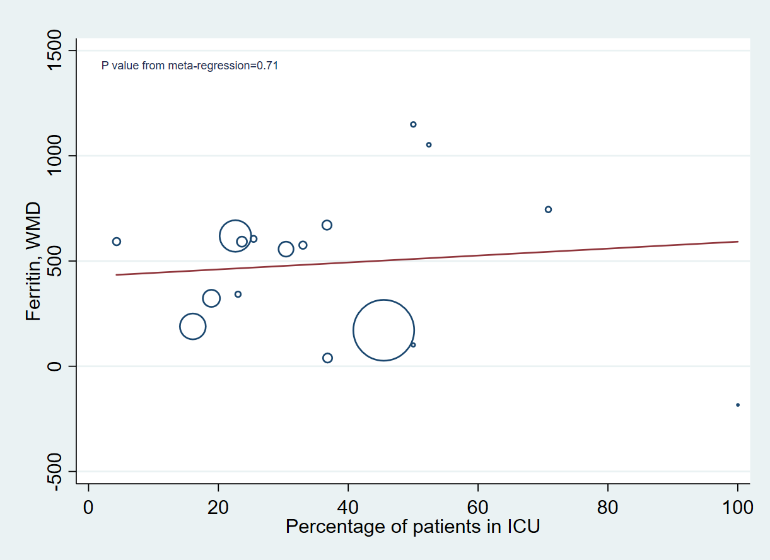

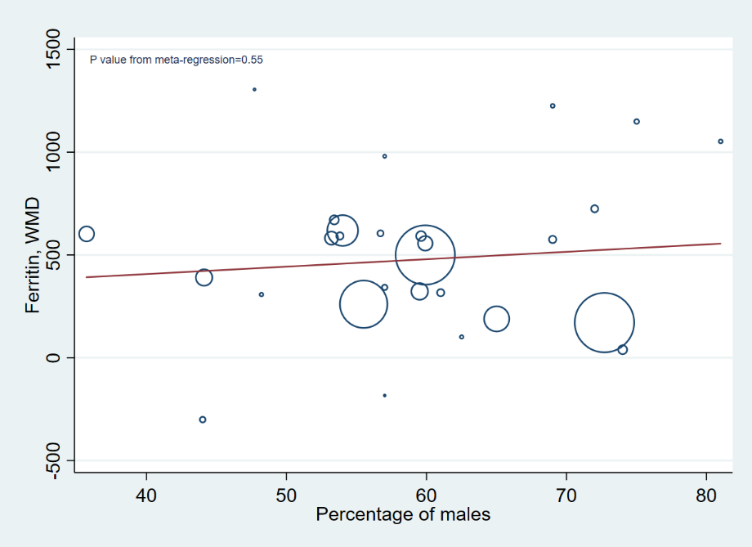

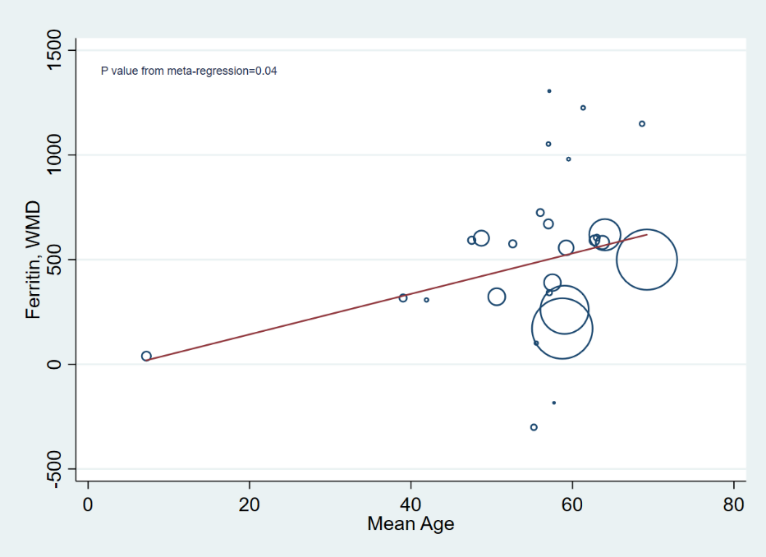

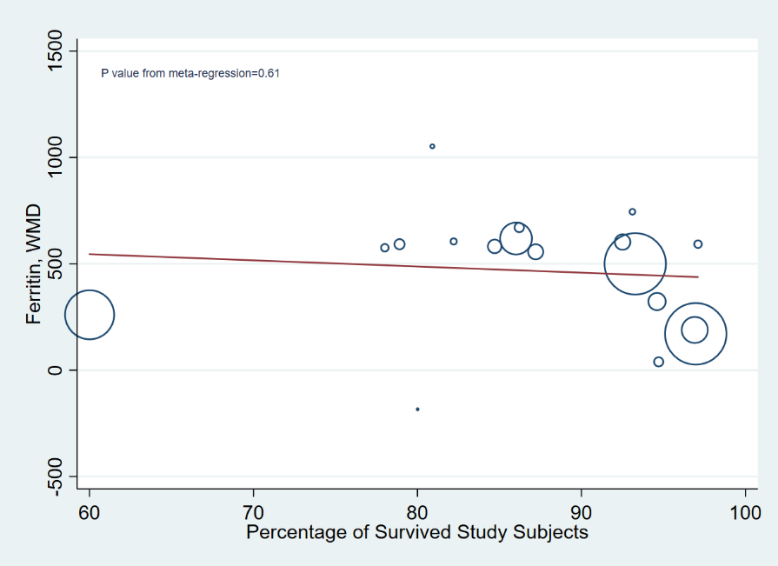
** **
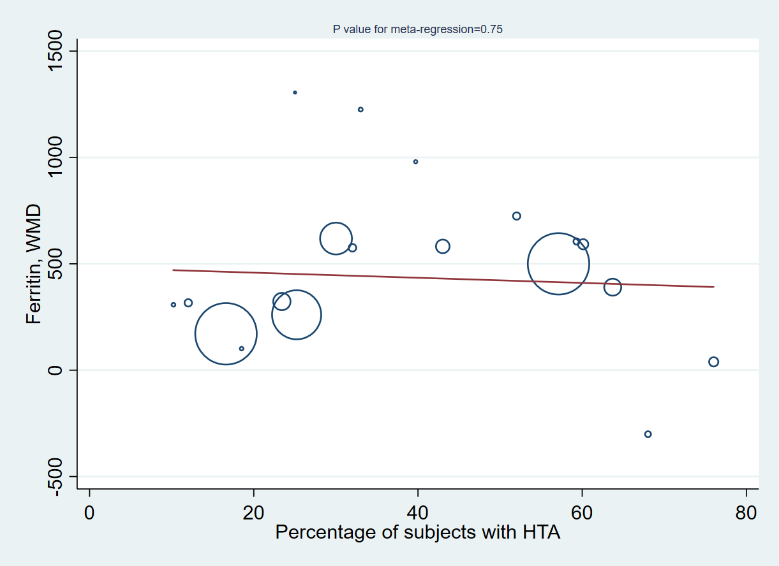

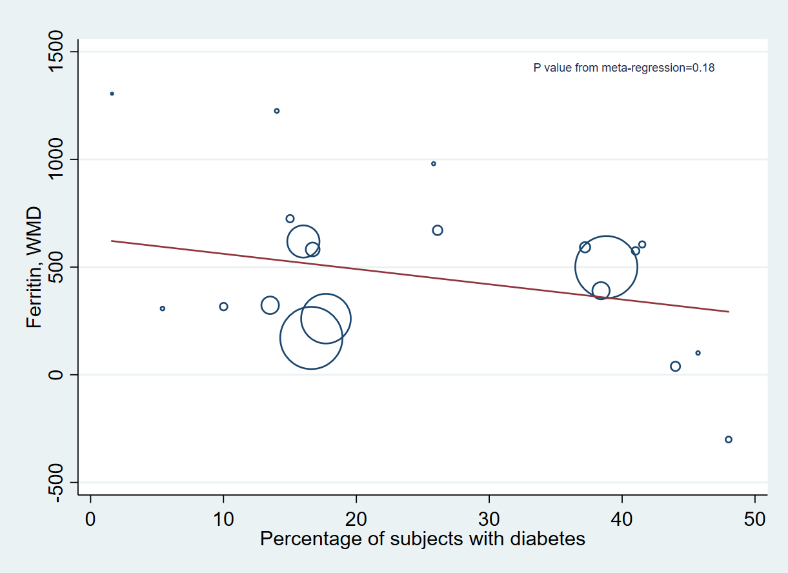

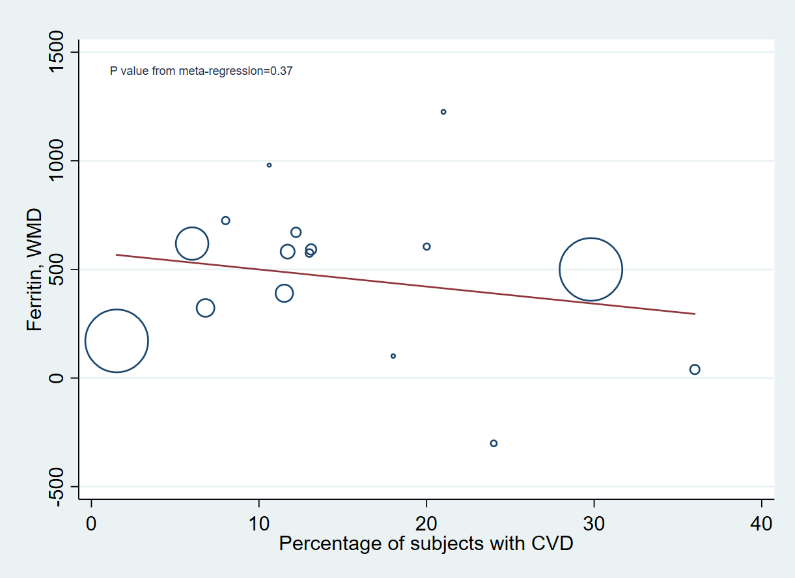
**

**Supplemental figures S12.** Bubble plots on weightened mean difference in ferritin levels between deceased vs survived Covid-19 cases by age, percentage of patients survived, percentage of male population, percentage of patients at intensive care and percentage of patients with comorbidities, perentage of patients with cardiovascular diseases, diabetes and hypertension

**
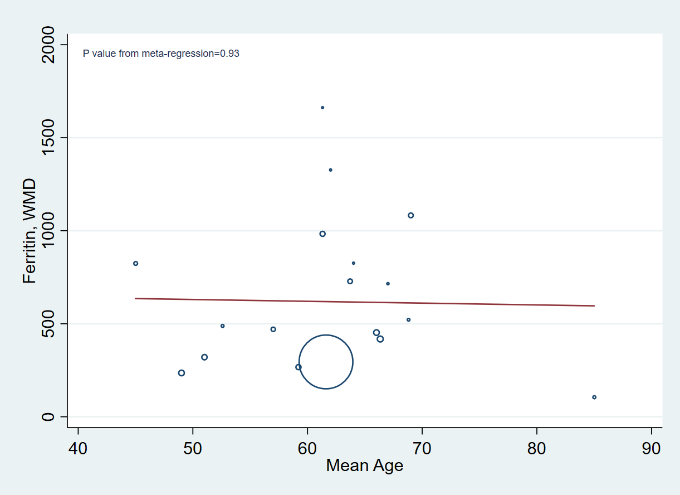

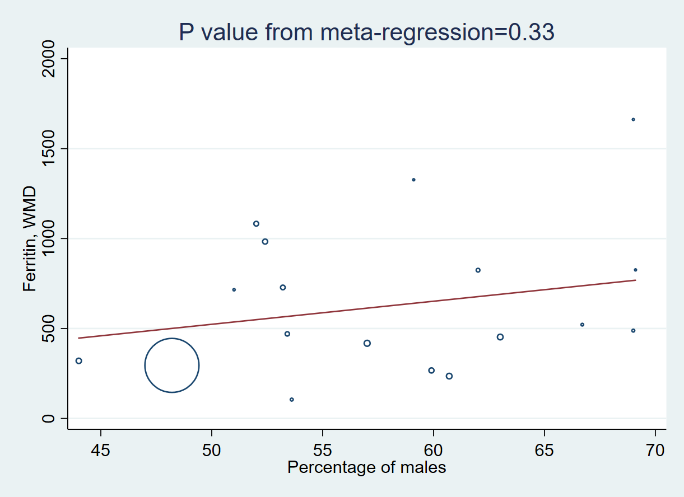

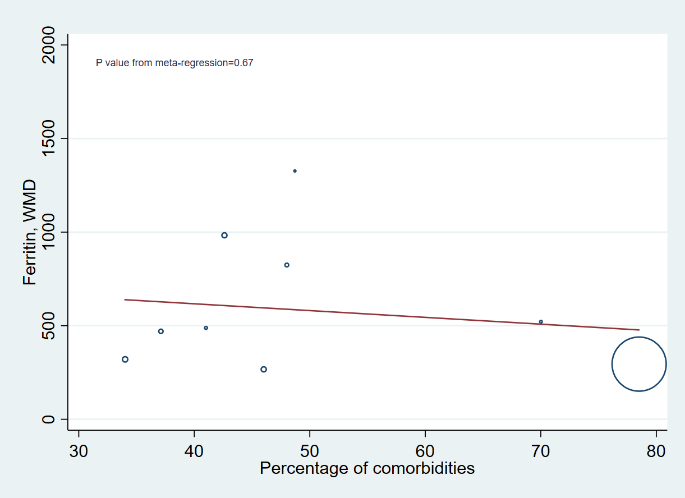

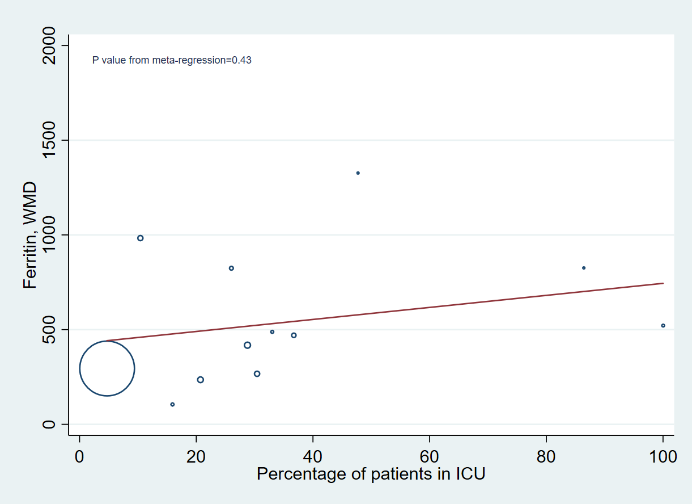

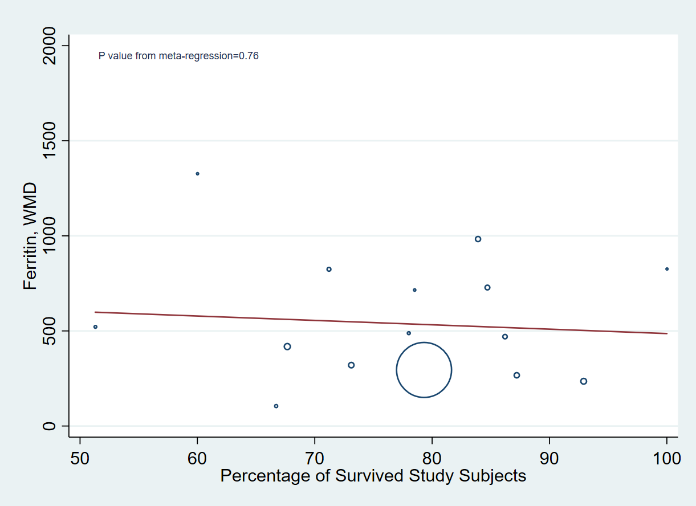

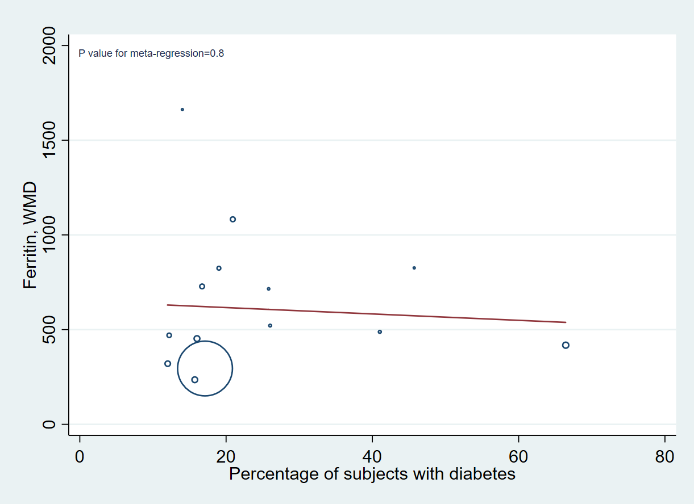

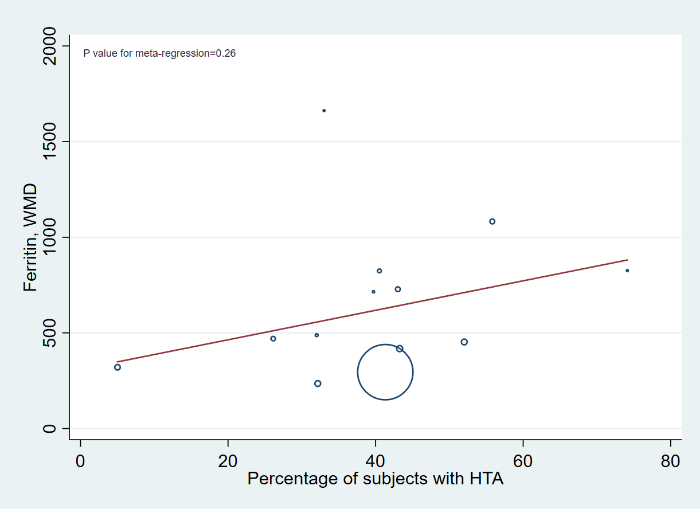

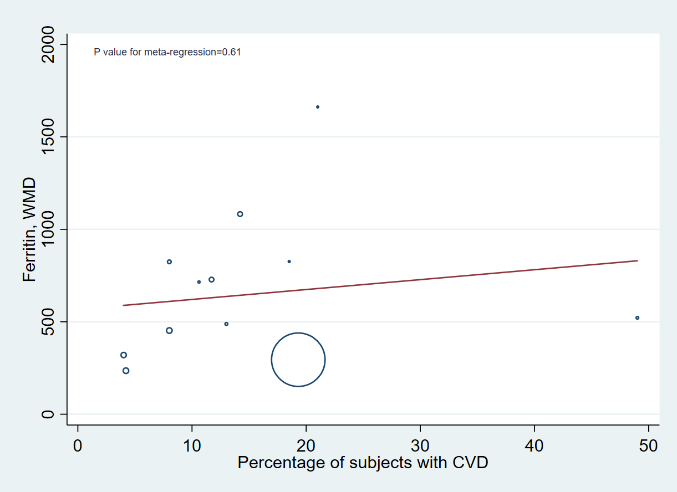
**

**Supplemental figures S13.** Bubble plots on mean red blood cells count by age, percentage of patients survived, percentage of male population, percentage of patients at intensive care and percentage of patients with comorbidities, perentage of patients with cardiovascular diseases, diabetes and hypertension

**
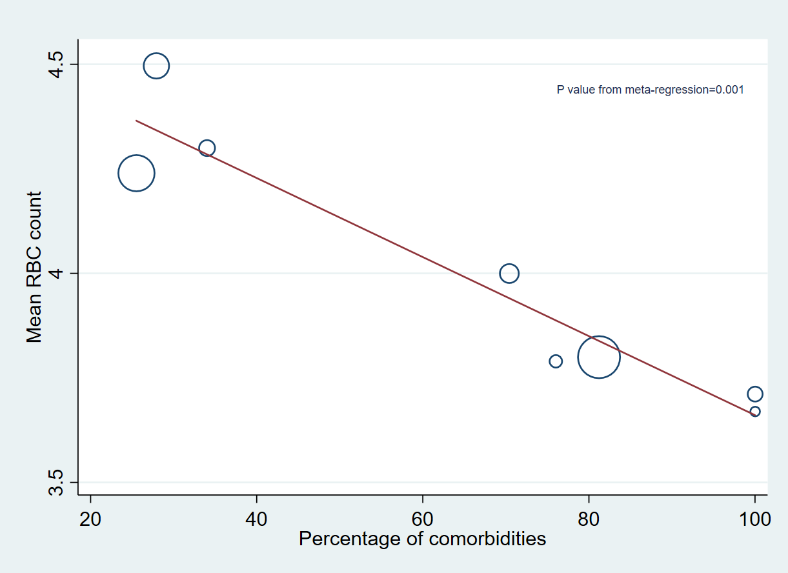

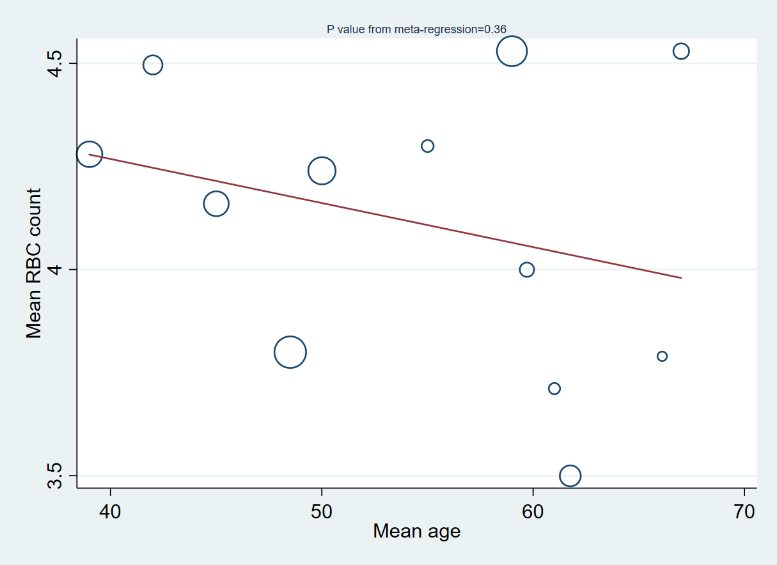

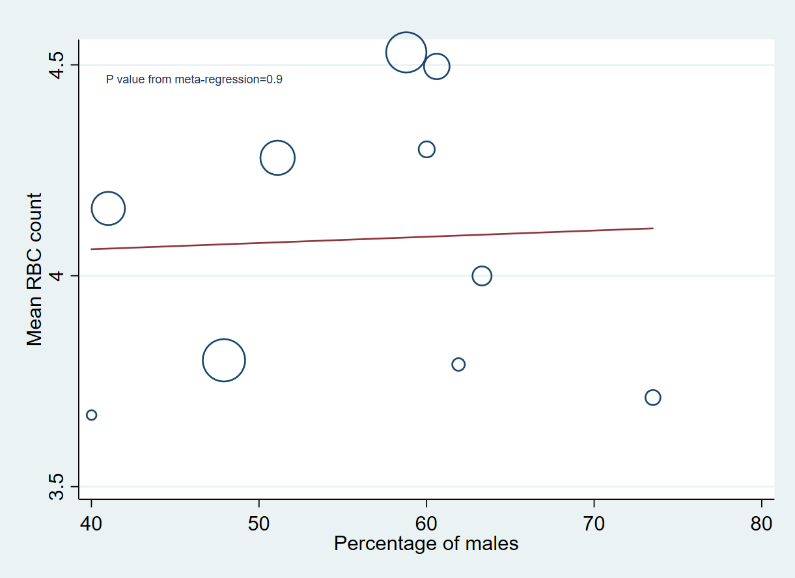
**

**Supplemental figures S14**. Publication bias for the meta-analysis on differences in mean hemoglobin levels between severe and moderate COVID-19 patients (a) and between deceased and survived Covid-19 cases (b)

a)


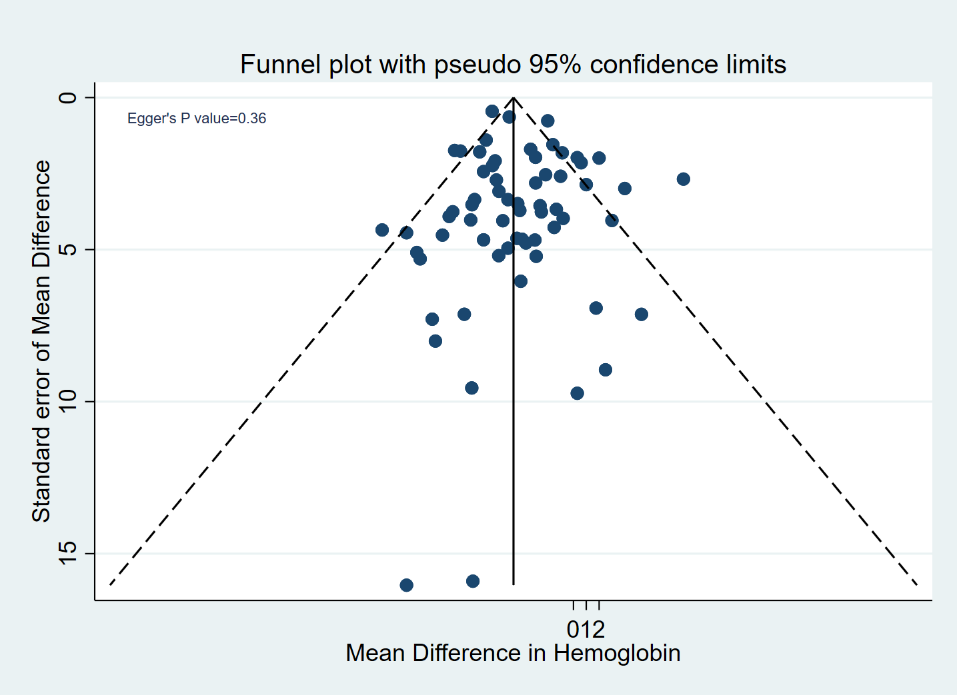


b)
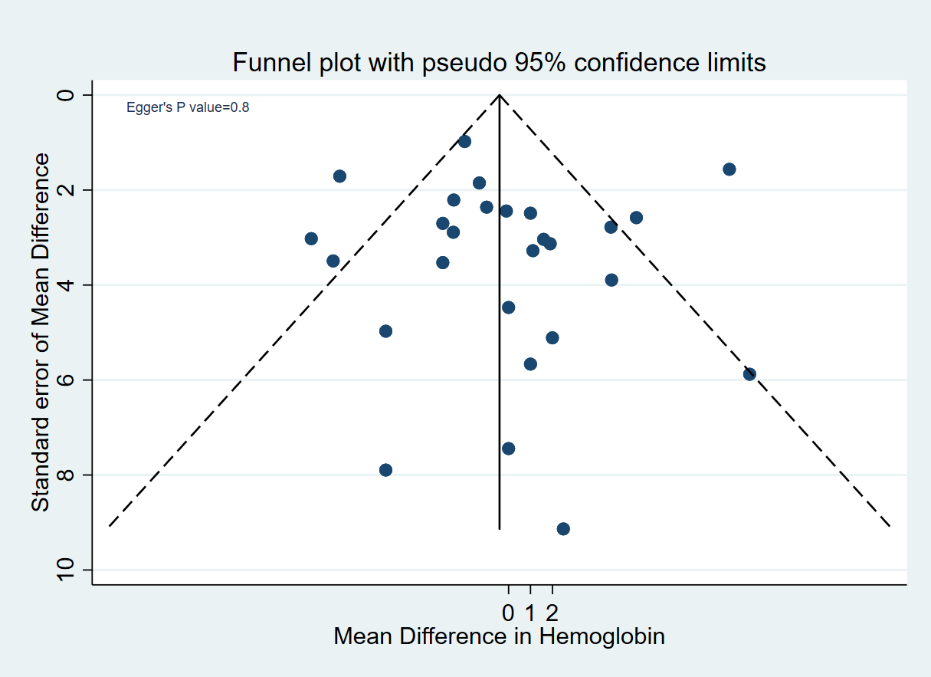


**Supplemental figures S15**. Publication bias for the meta-analysis on differences in mean ferritin levels between severe and moderate COVID-19 patients (a) and between deceased and survived Covid-19 cases (b)

a
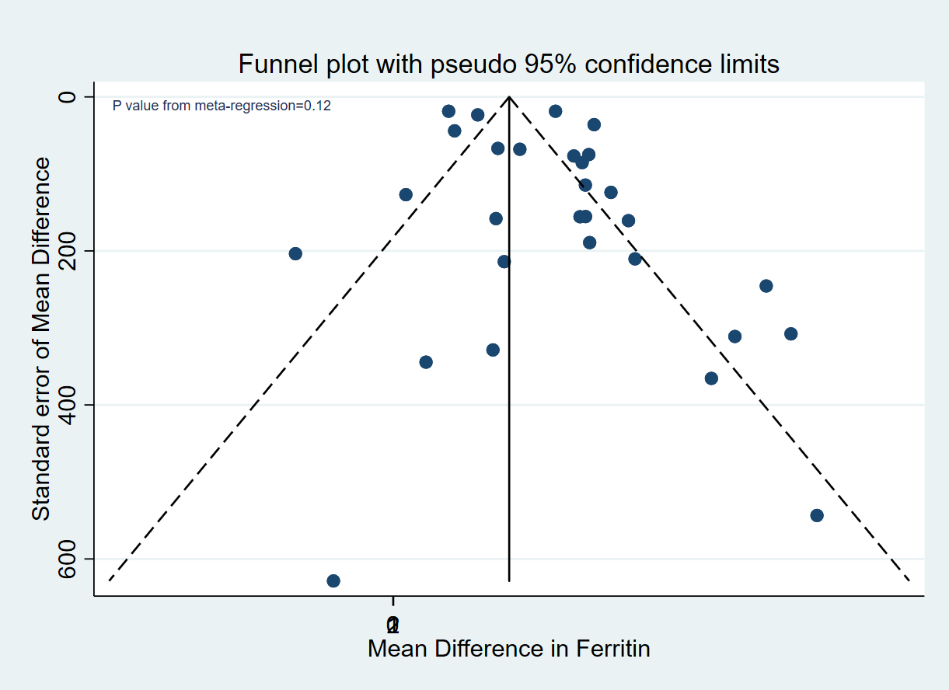


b


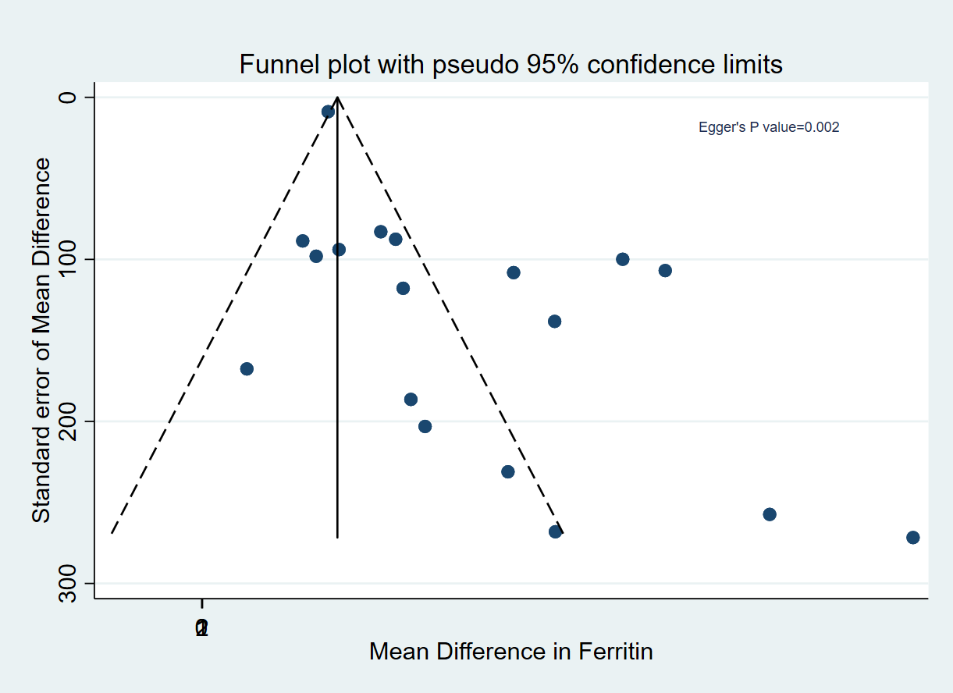


**Supplemental figure S16**. Publication bias for the meta-analysis on differences in mean red blood cells count levels between severe and moderate COVID-19 patients


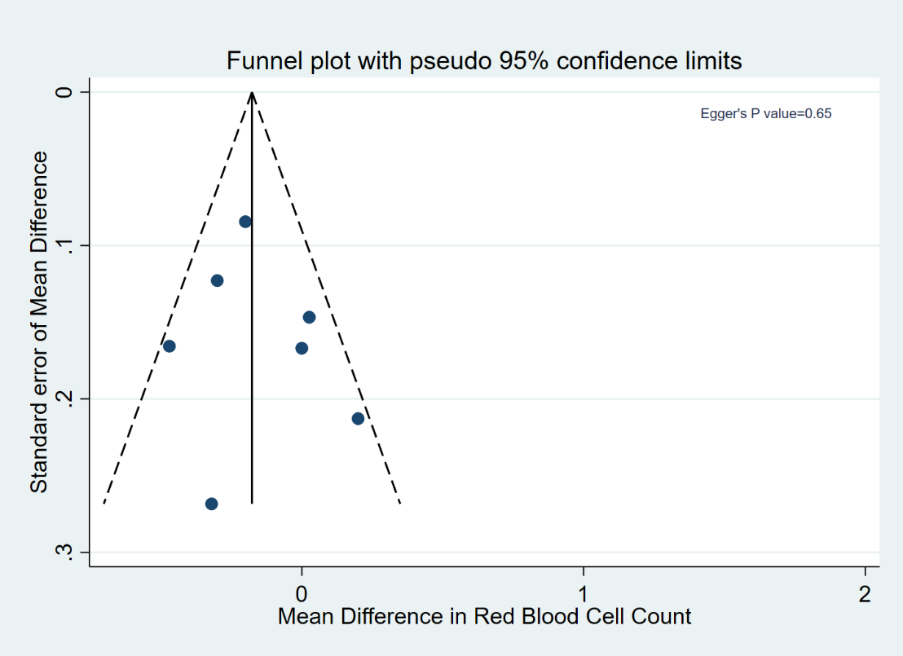


| **Supplemental table S1. Characteristics of studies included in current review** | | | | | | | | | | | | | |
| --- | --- | --- | --- | --- | --- | --- | --- | --- | --- | --- | --- | --- | --- |
| **DOI** | **Author name** | **Study Design** | **No. of individuals** | **Study location** | **Mean Age (SD)** | **Pediatric/**  **Adult/Mix population** | **Percentage of males(n%)** | **Survival** | **Percentages of individuals with comorbidities** | **Ferritin** | **Hemoglobin** | **Other biomarkers** | **Study Quality** |
| 10.1093/cid/ciaa632 | Buckner et al. | Cross-sectional | 105 | USA | 63 ± 55.6 | Adult | 50,00 | 67 | 93,00 |  | X |  | 7 |
| 10.1186/s40249-020-00703-5 | Cai et al. | Retrospective multicenter study | 96 | China | 38.7 ± 18.49 | Mix | 56,3 | N/A | N/A |  | x |  | 7 |
| 10.1371/journal.pone.0234764 | Cao et al. | Retrospective Cohort Study | 80 | China | 53 ± 20 | Adult | 47,5 | 96,2 | N/A |  | x |  | 8 |
| 10.1016/j.cmi.2020.05.041 | Cen et al. | Prospective cohort study | 1007 | China | 59.33 ± 14.1 | Mix | 49 | 93,6 | 36,1 |  | x |  | 6 |
| 10.1016/j.jpeds.2020.05.006 | Chao et al. | Retrospective | 67 | USA | 8.99 ± 12.16 | Children | 67,4 | 100 | N/A |  | x |  | 8 |
| 10.1016/j.jcrc.2020.07.003 | Chen et al. | Retrospective Cohort Study | 681 | China | 63.7 ± 13.4 | Adult | 53,2 | 84,7 | N/A |  | x |  | 9 |
| 10.1016/j.jaci.2020.05.003 | Chen eta al. | Retrospective | 548 | China | 56 ± 14.5 | Adult | 57,1 | 81,2 | 44 | x | x | x | 8 |
| 10.1111/tid.13378 | Chen et al. | Retrospective | 30 | USA | 56 ± 12 | Adult | 53 | 80 | 100 | x |  |  | 7 |
| 10.1111/bjh.16885 | Chen et al. | Retrospective | 271 | China | 57.74 ± 14.30 | Adult | 53,5 | 97 | N/A |  | x |  | 9 |
| 10.1016/j.ijid.2020.06.091 | Chen et al. | Retrospective | 267 | China | 48.33 ± 0.87 | Mix | 45,3 | 84,16 | 39,3 |  | x |  | 9 |
| https://doi.org/10.2337/dc20-0660 | Chen et al. | Retrospective | 341 | China | 54 ± 14.89 | Mix | 53,67 | N/A | N/A |  | x |  | 8 |
| 10.1016/j.ijantimicag.2020.106110 | Cheng et al | Retrospective | 305 | Wuhan, China | 62.67 ± 14.15 | Adult | 60,3 | 72,1 | 57 |  | x |  | 8 |
| 10.3390/jcm9061959 | Choi et al. |  | 293 | Korea | 33.3 ± 17.13 | Mix | 73 | 70,6 | N/A |  | x |  | 8 |
| 10.1111/ggi.13960 | Covino et al. | Retrospective | 79 | Italy | 85 ± 5.3 | Adult | 53,6 | 66,7 | N/A | x | x |  | 8 |
| 10.1159/000509517 | Cui et al. | Retrospective | 116 | Wuhan, China | 59.02 ± 14.64 | Adult | 56,9 | 84,5 | N/A | x |  |  | 7 |
| 10.1016/S0140-6736(20)31189-2 | Cummings, | Prospective cohort study | 257 | USA | 61.67 ± 15.66 | Adult | 67 | 61 | 82 | x |  |  | 7 |
| 10.1016/j.cca.2020.07.018 | Deng et al. | Retropsective | 264 | Wuhan, China | 63.93 ± 15.43 | Adult | 49,2 | 80,3 | N/A |  | x |  | 8 |
| 10.1042/bsr20200817 | Djakpo et al. | Retrospective | 208 | Hubei, China | 50 ± 17.48 | Adult | 51,4 | N/A | 25,5 |  | x | x | 6 |
| 10.14336/AD.2020.0317 | Dong et al. | Retrospective | 18 | China | 58.39 ± 17.21 | Adult | 61,1 | 100 | 93,00 |  | x |  | 8 |
| [10.2147/JIR.S257078](about:blank) | Gholizadeh | Retrospective | 279 | Iran | 58,99 | Adult | 58,78 | 71,77 | N/A |  | x |  | 8 |
| 10.7150/ijms.46614 | Pan F | Case-controls study | 124 | China | 68 | Adult | 68,5 | N/A | N/A |  | x |  | 7 |
| 10.1002/jcla.23475 | Peng | Cross-sectional | 190 | China | 46,35 | Adult | 47 | 77 | N/A |  | x |  | 7 |
| 10.1002/HEP.31404 | Phipps | Retrospective cohort | 2273 | USA | 65 | Adult | 57 | N/A | N/A | x |  |  | 8 |
| 10.1111/liv.14565 | Piano | Retrospective cohort | 565 | Italy | 66 | Adult | 63 | N/A | N/A | x | x |  | 7 |
| 10.1002/ccd.29114 | Popovic | Retrospective cohort | 11 | France | 63,6 | Adult | 63,9 | N/A | N/A |  | x |  | 7 |
| 10.1016/j.amsu.2020.07.020 | Samrah | Cross-sectional study | 81 | Jordania | 39,95 | Adult | 45,7 | 80 | 30,9 | x |  |  | 8 |
| 10.1186/s13054-020-03051-w | Shah | Retrospective cohort | 30 | UK | 57.7 | Adult | 57 | 8 | N/A | x | x |  | 6 |
| 10.1186/s12879-020-05128-x | Shahriarirad | Retrospective cohort | 113 | Iran | 53,75 | Adult | 37,2 | 68,94 | N/A |  | x |  | 8 |
| 10.1002/jcla.23415 | Shi | Retrospective cohort | 161 | China | 59,38 | Adult | 64,6 | 71,77 | N/A |  | x |  | 9 |
| 10.1002/jmv.26031 | Shang | Retrospective cohort | 443 | China | 61 | Adult | 49 | N/A | N/A |  | x |  | 8 |
| 10.1186/s12933-020-01035-2 | Ren | Retrospective cohort | 151 | China | 59,5 | Adult | 51,7 | N/A | N/A | x |  |  | 8 |
| 10.1002/jmv.26252 | Salacup | Retrospective Cohort | 242 | USA | 57 | Adult | 57 | N/A | N/A | x |  |  | 8 |
| 10.1093/eurheartj/ehaa408 | Shi S | Retrospective Cohort | 671 | China | 63 | Adult | 48 | N/A | N/A |  | x |  | 8 |
| 10.1186/s12916-020-01633-7 | Shi J | Prospective cohort | 85 | China | 46,6 | Adult | 57,6 | N/A | N/A |  | x |  | 8 |
| 10.1053/j.gastro.2020.04.064 | Singh | Retrospective cohort | 2780 | USA | 55,2 | Adult | 44 | N/A | N/A | x | x |  | 8 |
| 10.1007/s10456-020-09730-0 | Smadja | Retrospective cohort | 40 | France | 55 | Adult | 70 | N/A | N/A |  | x |  | 7 |
| 10.1093/ofid/ofaa171 | Song | Retrospective cohort | 111 | China | 55 | Adult | 55,86 | N/A | 30,63 |  | x |  | 8 |
| 10.1038/s41467-020-17240-2 | Song J | Cross-sectional | 41 | China | 39 | Mix | 61 | 95 | 24 | x | x |  | 8 |
| 10.1001/jamanetworkopen.2020.12270 | Suleyman | Retrospective cohort | 463 | China | 57,5 | Adult | 44,1 | N/A | N/A | x |  |  | 9 |
| 10.1111/jgs.16533 | Sun H | Retrospective case-controls study | 244 | China | 69 | Adult | 52 | N/A | N/A | x |  |  | 8 |
| 10.22514/sv.2020.16.0019 | Sungurtekin | Retrospective cohort | 24 | Turkey | 24 | Adult | 65,5 | N/A | N/A | x |  |  | 8 |
| 10.21203/rs.3.rs-39184/v1 | Tao | Retrospective cohort | 222 | China | 55 | Adult | 36 | N/A | N/A |  | x |  | 7 |
| 10.1016/j.autrev.2020.102568 | Toniati | Prospective cohort | 100 | Italy | 62 | Adult | 88 | N/A | N/A | x | x |  | 8 |
| 10.2217/bmm-2020-0317 | Usul | Retrospective cohort | 163 | Turkey | 47,6 | Adult | 62,8 | N/A | N/A |  | x |  | 7 |
| 10.1101/2020.04.09.20058594 | Wang | Retrospective cohort | 161 | China | 39 | Adult | 51,1 | N/A | N/A |  | x | x | 8 |
| 10.1186/s12967-020-02423-8 | Wang F | Retrospective cohort | 333 | China | 46 | Adult | 47,7 | N/A | 22,3 | x |  |  | 9 |
| <https://doi.org/10.1093/cid/ciaa443> | Gong | Retrospective | 189 | China | 48,41 | Adult | 46,56 | N/A | 29,1 |  | x | x | 8 |
| PubMed ID: 32378815 | Itelman | Descriptive study | 162 | Israel | 56,35 | Adult | 65 | 96,9 | N/A | x |  |  | 8 |
| 10.22514/sv.2020.16.0023 | Ipekci | Retrospective | 51 | Turkey | 51,27 | Adult | 72,5 | 92,2 | N/A | x |  |  | 7 |
| 10.3906/sag-2006-164 | Guner | Cross-sectional | 222 | Turkey | 50,6 | Adult | 59,5 | 94 94,6996 | 41,4 | x | x |  | 8 |
| 10.3389/fpubh.2020.00299 | Gan | Retrospective | 95 | China | 65,5 | Adult | 61 | 58,9 58,95 | 72 |  | x |  | 8 |
| doi: 10.3389/fmed.2020.00347 | Jiang | Retrospective | 131 | China | 51,2 | Adult | 53,4 | v N/A | N/A |  | x |  | 8 |
| https://doi.org/10.3346/jkms.2020.35.e209 | Jang | Retrospective | 110 | Korea | 56,9 | Adult | 43,6 | 92,7 92,7 | 44,5 |  | x |  | 8 |
| http://doi.org/10.2147/IDR.S263489 | Ghweil | Retrospective | 66 | Egypt | 58,72 | Adult | 72,7 | 96,96 96,96 | 34,85 | x | x |  | 7 |
| https://doi.org/10.1016/j.jiac.2020.05.005 | Kato | Cross-sectional | 70 | Japan | 66,67 | Adult | 67,1 | 97,1 97,144 | N/A |  | x |  | 7 |
| doi: 10.5858/arpa.2020-0389-SA | Aloisio et al | Cross sectional | 243 | Italy | 61.33 ± 17.15 | Adult | 69 | 79,16 | N/A | x |  |  | 8 |
| DOI 10.1182/blood.2020006520. | Al-Samkari et al | retrospective cohort | 400 | USA | 62.03 ± 13.10 | Adult | 57 |  | N/A | x |  |  | 8 |
| doi: 10.1136/bmj.m1996 | Argenziano et al | retrospective cohort | 283 | USA | 62.67 ± 18.56 | Adult | 59,6 | 78,9 | N/A | x |  |  | 8 |
| DOI: 10.7759/cureus.8712 | Asghar et al | Cross sectional | 100 | Pakistan | 52.58 ± 15.68 | Adult | 69 | 80 | 41 |  | x | x | 8 |
|  | Ayanian et al | Cross sectional | 252 | USA | - | Adult | 54 | 76,3 | N/A | x |  |  | 6 |
| DOI:10.1111/hdi.12861 | Bahat et al | Cross sectional | 25 | Turkey | 60.5 ± 15 | Adult | 40 | 80 | N/A |  | x |  | 8 |
| https://doi.org/10.1186/s40164-020-00172-4 | Bao et al | Cross sectional | 178 | China | 62.67 ± 11.96 | Adult | 59,6 | 96,1 | N/A |  | x |  | 8 |
| DOI: 10.1212/WNL.0000000000009848 | Benussi et al | Cross sectional | 56 | Italy | 70.1 ± 17.2 | Adult | 53,8 | 85 | N/A | x | x |  | 7 |
| https://doi.org/10.1515/cclm-2020-0459 | Bhumbra et al | Observational cohort | 14 | USA | 7.27 ± 12.17 | Children | 74 | 94,7 | 42 | x |  |  | 7 |
| doi.org/10.1186/s13017-020-00323-2 | Bolondi et al | Observational cohort | 31 | Italy | 63.35 ± 8.59 | Adult | 81 | 74,2 | N/A | x |  |  | 7 |
| https://doi.org/10.1515/cclm-2020-0459 | Bonetti et al | Cross sectional | 144 | Italy | 68.83 ± 14.98 | Adult | 66,7 | 51,3 | N/A | x | x |  | 8 |
| doi:10.3390/jcm9061733 | Borobia et al | Cross sectional | 2226 | Spain | 61.67 ± 23.74 | Adult | 48,2 | 79,3 | 78,5 | x |  |  | 8 |
| DOI: 10.1111/eci.13314 | Masetti et al | Cross sectional | 229 | Italy | 60.7 ± 14.2 | Adult | 64,6 | 85,6 | 54,1 | x |  |  | 8 |
| 10.1016/j.ijid.2020.07.017 | Lee J Y et al | Cross sectional | 1251 | South Korea | 55,9 | Adult | 30,50 | 97,4 | 18,00 |  | X |  | 8 |
| 10.7150/thno.46569 | Li L et al | Cohort | 93 | China | 51 | Adult | 44 | N/A | 34,00 | X |  |  | 8 |
| 10.1172/jci.insight.138070. | Li S et al | Cohort | 69 | China | 47,5 | Adult | 53,8 | N/A | N/A | X |  |  | 9 |
| 10.1016/j.archger.2020.104185 | Li T. et al | Cross sectional | 312 | China | 69,20 | Adult | 59,9 | 93,27 | 77,2 | X | X |  | 7 |
| 10.1016/j.jrid.2020.05.003 | Li X et al | Cross sectional | 73 | China | 51 | Adult | 38,4 | 0 | N/A |  | X | x | 8 |
| 10.1016/j.diabres.2020.108299 | Li Y et al | Cross sectional | 132 | China | 65 | Adult | 53 | 88,6 | 66,7 | X | X |  | 8 |
| 10.3389/fmicb.2020.01570 | Li Y et al | Cross sectional | 178 | China | 46 | Adult | 50,6 | 0 | N/A |  | X |  | 8 |
| 10.1016/j.ijid.2020.06.026 | Li Y et al | Cross sectional | 116 | China | 18,7 | Pediatric | 61,4 | 0 | N/A |  | X |  | 8 |
| 10.1016/j.ijid.2020.05.008 | Li Y et al | Cross sectional | 18 | China | 39 | Adult | 61,1 | 0 | N/A |  | X |  | 8 |
| 10.18632/aging.103582 | Lian J et al | Cross sectional | 232 | China | 67,29167 | Adult | 46,98 | 60 | 49,1 |  | X |  | 7 |
| 10.1093/cid/ciaa242. | Lian J et al | Cohort | 788 | China | 45,8 | Adult | 42,65 | 100 | 28 |  | X |  | 9 |
| 10.1016/S2352-3026(20)30217-9 | Liao D et al | Cross sectional | 384 | China | 64 | Adult | 54 | 86,00 | 48 | X | X |  | 8 |
| [10.1016/j.jinf.2020.06.053](https://dx.doi.org/10.1016%2Fj.jinf.2020.06.053) | Lin et al | Cross sectional | 147 | China | 41,9 | Adult | 48,2 | N/A | N/A | X |  |  | 5 |
| 10.1186/s13613-020-00706-3 | Liu J et al | Cross sectional | 1190 | China | 57 | Adult | 53,4 | 86,8 | 37,1 | X | X |  | 9 |
| 10.3389/fcimb.2020.00284 | Liu L et al | Cross sectional | 53 | China | 38 | Adult | 49 | 70,5 | 49 |  | X |  | 8 |
| 10.15252/emmm.202012421 | Liu T et al | Cross sectional | 80 | China | 53 | Adult | 42,5 | 0 | 35 | X | X |  | 8 |
| 10.1371/journal.pone.0235459 | Liu X et al | Cross sectional | 104 | China | 42 | Mixed | 60,6 | N/A | 27,9 |  |  | x | 8 |
| 10.1016/j.micinf.2020.06.005 | Lohse et al. | Cross sectional | 34 | France | 75 | Adult | 70,5 | N/A | N/A | X | X |  | 7 |
| 10.3855/jidc.12678 | Lu R et al | Cross sectional | 28 | China | 48,3 | Adult | 60,7 | N/A | N/A | X | X |  | 7 |
| 10.1016/j.ijid.2020.04.078 | Luo Y et al | Cross sectional | 1027 | China | 52 | Adult | 50,3 | N/A | N/A |  | X | x | 8 |
| 10.1016/j.ekir.2020.06.003 | Ma Y et al | Cross sectional | 42 | China |  | Adult | 73,5 | N/A | 100 |  |  | x | 7 |
| 10.1002/jmv.26365 | Maeda T et al | Cross sectional | 224 | USA | 63 | Adult | 56,7 | 82,2 | N/A | X | X |  | 8 |
| 10.1164/rccm.202005-1583OC | McElvaney O, et al. | Cross sectional | 40 | Ireland | 55,5 | Adult | 62,5 | N/A | N/A |  | x |  | 8 |
| 10.1007/s11606-020-05983-z | Mikami T et al | Cross sectional | 6493 | US | 59 | Adult | 55,5 | 60 | N/A | X | X |  | 9 |
| 10.1111/tid.13420 | Monfared A | Cross sectional | 22 | Iran | 52 | Adult | 68,2 | 72,7 | 100 |  | X |  | 7 |
| 10.1016/j.jaut.2020.102512. | Morrison AR | Clinical trial | 71 | US | 64 | Adult | 69,1 | N/A | N/A | X |  |  | 7 |
| 10.1590/1806-9282.66.6.746 | Nalbant A | Cross sectional | 54 | Turkey | 53 | Adult | 53 | 0 | N/A |  | X |  | 8 |
| 10.1111/1742-6723.13573 | O Reilly et al* | Cross sectional | 14 | Australia | 14 | Adult | 79 | 100 | N/A |  | X |  | 8 |
| 10.24875/RIC.20000211 | Ortiz Brizuela et al | Cohort | 140 | China | 49 | Adult | 60,7 | 92,9 | N/A | X | X |  | 7 |
| https://doi.org/10.3390/cancers12071992 | Duployez N et al. | Retrospective cohort | 122 | France | 61.75 ±14.2 | Adult | 76 | 85 | N/A |  | x | x | 7 |
| 10.1002/HEP.31446 | Fu Y et al. | Retrospective | 482 | China | 54.3±21.0 | Adult | 50,4 | 86,7 | N/A |  | x |  | 6 |
| https://doi.org/10.1016/j.ajic.2020.07.005 | Gavin W et al | Retrospective | 140 | USA | 60±17.98 | Adult | 51,4 | 84,3 | N/A |  | x |  | 7 |
| https://doi.org/10.3201/eid2608.201776 | Ferguson J et al. | Retrospective | 72 | USA | N/A | Adult | N/A | 93,1 | N/A | x |  |  | 8 |
| 10.1002/jmv.26306 | Gayam V et al. | Retrospective cohort | 408 | USA | 66.33±14.98 | Adult | 57 | 67,65 | N/A |  | x |  | 8 |
| https://doi.org/10.1016/j.phrs.2020.104931 | Giacomelli A et al | Prospective cohort | 233 | Italy | 61±16.41 | Adult | 69,1 | 79,4 | N/A |  | x | x | 7 |
| https://doi.org/10.1016/j.kint.2020.04.031 | Goicoechea M et al | Retrospective cohort |  | Spain | 71± 12 | Adult | 64 | 69,5 | N/A |  | x |  | 7 |
| 10.1159/000508734 | Guo Et al | Retrospective | 105 | China | 68.3±7.52 | Adult | 55,7 | 96,9 | 54,3 |  | x |  | 7 |
| 10.1001/jamainternmed.2020.3596 | Gupta S et al. | Retrospective | 2215 | USA | 60.5±14.5 | Adult | 64,8 | 60,6 | 100 |  | x |  | 9 |
| https://doi.org/10.1016/j.jaci.2020.05.008 | Herold T et al. | Prospective | 89 | Germany | 56±13 | Adult | 72 | N/A | 80 | x |  |  | 9 |
| - | Huang JT et al. | Retrospective | 308 | China | 62±14 | Adult | 48,6 | 94,8 | N/A |  | x |  | 7 |
| https://doi.org/ 10.1371/journal.pntd.0008280 | Huang R et al. | Retrospective | 202 | China | 43.6±15.6 | Adult | 57,4 | 100 | 27,2 |  | x |  | 7 |
| 10.1093/eurheartj/ehaa415 | Inciardi RM et al. | Retrospective | 99 | Italy | 67 ± 12 | Adult | 81 | 74 | N/A | x | x | x | 7 |
| 10.2478/rjim-2020-0013 | Javanian M et al. | Retrospective cohort | 100 | Iran | 57±15 | Adult | 51 | 81 | N/A |  | x |  | 7 |
| https://doi.org/10.1017/S0950268820001557 | Kong M et al. | Retrospective cohort | 210 | China | 59.3 ± 16.0 | Adult | 49,5 | N/A | 47,1 |  | x |  | 7 |
| - | Lagadinou M et al. | Retrospective | 64 | Greece | 57.11±16.3 | Adult | 47,7 | N/A | 30,8 | x |  |  | 7 |
| [https://doi.org/10.1093/ofid/ofaa187](about:blank) | Wang et al. | Cross-sectional study | 275 | China | 48.3 (20.9) | Adult | 46,50 | 100,0 | 33,10 |  | x |  | 8 |
| https://doi .org/10.1128/mSphere.00362-20. | Wu et al. | Cross-sectional | 71 | China | 59.7 (15.1) | Adult | 63,30 | 100,0 | 70,40 |  | x | x | 8 |
| http://dx.doi.org/10.21037/atm-20-3192 | Xu et al. | Multicenter retrospective study | 32 | China | 8.7 (4.7) | Pediatric | 53,00 | 100,0 | 9 |  | x |  | 7 |
| https://doi.org/10.1186/s13054-020-03098-9 | Xu et al. | Multicenter retrospective study | 239 | China | 62.5 (12.2) | Adult | 59,80 | 38,5 | 67,80 |  | x |  | 9 |
| [https://doi.org/10.1016/j.jcv.2020.104475](about:blank) | Yuang et al. | Descriptive study | 200 | China | 55 (17.1) | Adult | 49 | 92,5 | 32,50 |  | x |  | 7 |
| https://doi.org/10.1186/s40560-020-00466-z | Yao et al. | Case-control | 249 | China | 63 (13.4) | Adult | 54,20 | 93,1 | 32,50 |  | x |  | 9 |
| https://doi.org/10.1016/j.jiph.2020.07.002 | Yu et al. | Retrospective study | 1663 | China | 62.3 (14.1) | Adult | 50,40 | 89,8 | 39 |  | x |  | 10 |
| https ://doi.org/10.1007/s1218 5-020-02930 -w | Yuan et al. | Retrospective study | 117 | China | 48.5 (16.9) | Adult | 47,90 | N/A | 81,20 |  | x | x | 8 |
| https://doi.org/10.1016/j.jdiacomp.2020.107666 | Zhang et al. | Cross-sectional | 74 | China | 67 (17.4) | Adult | 48,60 | 86,5 | 74,30 |  | x |  | 9 |
| https://doi.org/10.1093/ofid/ofaa250 | Zhao et al. | Retrospective study | 50 | China | 55 (16.8) | Adult | 60 | 46,2 | 34 |  | x | x | 9 |
| https://doi.org/10.1002/jpen.1953 | Zhao et al. | Retrospective study | 413 | China | 60.31 (12.68) | Adult | 51 | 91,0 | 42 |  | x |  | 6 |
| [https://doi.org/10.1371/journal.pone.0236618](about:blank) | Zhao et al. | Retrospective study | 641 | China | 59.2 (16.7) | Adult | 59,90 | 87,2 | 46 | x |  |  | 6 |
| doi:10.1111/cts.12805 | Zhou et al. | Retrospective study | 21 | China | 66.1 (13.94) | Adult | 61,90 | 85,7 | 76 |  | x |  | 6 |
| 10.1136/bmj.m1092 | Chen et al. | Retrospective study | 274 | China | 58,6 (19,4) | Adult | 62,40 | 58,7 | 49 | x | x |  | 8 |
| 10.1016/S0140-6736(20)30211-7 | Chen et al. | Descriptive study | 99 | China | 55,5 (13,1) | Adult | 68 | 89,0 | 51 | x | x |  | 7 |
| 10.1172/JCI137244 | Chen et al. | Retrospective study | 21 | China | 57,0 (11,9) | Adult | 81 | 80,9 | 38,1 | x | x |  | 7 |
| 10.1016/j.kint.2020.03.005 | Cheng et al. | Prospective cohort study | 701 | China | 61,3 (15,6) | Adult | 52,4 | 83,9 | 42,60 |  | x |  | 8 |
| 10.1007/s15010-020-01427-2 | Du et al. | Retrospective study | 67 | China | 24,7 (7,2) | Mix | 47,8 | 100 | N/A |  | x |  | 8 |
| 10.1164/rccm.202003-0543OC | Du et al. | Retrospective study | 85 | China | 57,9 (21,1) | Mix | 72,90 | 0 | 68,20 |  | x |  | 6 |
| 10.1164/rccm.202002-0445OC | Feng et al. | Multi-center retrospective study | 476 | China | 52,3 (17,8) | Mix | 56,90 | 92 | 43,10 |  | x |  | 8 |
| 10.1056/NEJMoa2002032 | Guan et al. | Cross-sectional | 1099 | China | 46,7 (17,1) | Mix | 58,10 | 98,6 | 23,70 |  | x |  | 8 |
| 10.1007/s11427-020-1661-4 | Hu et al. | Cross-sectional | 24 | China | 35,00 (10,98) | Mix | 33,30 | 100 | N/A |  | x |  | 6 |
| 10.1016/S0140-6736(20)30183-5 | Huang et al. | Prospective | 41 | China | 49,25 (4,91) | Adult | 73,00 | 85 | 32,00 |  | x |  | 8 |
| 10.1016/j.tmaid.2020.101606 | Huang et al. | Retrospective | 34 | China | 56,24 (1,14) | Adult | 41,20 | 99,85 | 47,10 |  | x |  | 6 |
| 10.1136/gutjnl-2020-320926 | Jin et al. | Retrospective | 651 | China | 45,20 (14,43) | Adult | 50,84 | 90,76 | 27,34 |  | x |  | 8 |
| 10.1016/j.tmaid.2020.101664 | Lei et al. | Cross-sectional | 119 | China | 53,4 (13,3) | Adult | 64,70 | 0 | 47,80 |  | x |  | 7 |
| 10.1016/j.ijid.2020.03.053 | Li et al. | Retrospective | 25 | China | 75,25 (12,99) | Adult | 40,00 | 100 | 100 |  | x |  | 6 |
| 10.1016/j.jaci.2020.04.006 | Li et al. | Prospective | 548 | China | 59,25 (6,06) | Adult | 50,90 | 94,65 | 64,40 | x |  |  | 9 |
| 10.1093/cid/ciaa242 | Lian et al. | Retrospective | 788 | China | 45,83 (10,79) | Mix | 51,70 | 86,53 | 27,60 |  | x |  | 8 |
| 10.1016/j.jinf.2020.03.005 | Liu et al. | Cross-sectional | 56 | China | 52,48 ( 3,68) | Adult | 55,35 | 100 | N/A |  | x |  | 8 |
| 10.1016/j.jinf.2020.04.002 | Liu et al. | Retrospective | 245 | China | 53,95 (16,90) | Adult | 46,53 | 85 | N/A |  | x |  | 8 |
| 10.14309/ajg.0000000000000620 | Pan L et al. | Cross-sectional study | 204 | China | 52,91 (15,98) | Adult | 52,45 | 17,65 | N/A |  | x |  | 8 |
| 10.1002/ctm2.23. | Pan Y et al. | Case-controls study | 84 | China | 58,6 (15,6) | Adult | 60,71 | N/A | N/A |  | x |  | 7 |
| 10.1016/j.jcv.2020.104353 | Tan et al. | Retrospective study | 10 | China | 7,1 (3,6) | Pediatric | 30,0 | 100 | 0 | x |  |  | 6 |
| 10.1016/j.ijid.2020.03.070 | Wang et al. | Retrospective cohort | 125 | China | 38,76 (13,7) | Mix | 56,8 | N/A | 27,20 |  | x |  | 6 |
| 10.1016/j.jinf.2020.03.019 | Wang L et al. | Retrospective cohort | 339 | China | 70 (8,18) | Adult | 49 | 80,82 | N/A |  | x |  | 8 |
| 10.1093/cid/ciaa272 | Wang Z et al. | Retrospective cohort | 69 | China | 46,33 (20,44) | Mix | 46 | 7,5 | N/A |  | x |  | 7 |
| 10.1001/jamainternmed.2020.0994 | Wu C et al. | Retrospective cohort | 201 | China | 51,3 (12,6) | Mix | 63,7 | 78,1 | 32.8 | x |  |  | 6 |
| 10.1093/cid/ciaa199 | Wu J et al. | Cross-sectional study | 80 | China | 46,1 (15,42) | Mix | 48,75 | 100 | 47,50 |  | X |  | 7 |
| 10.1016/S2213-2600(20)30079-5 | Yang X et al. | Retrospective cohort | 52 | China | 51,9 (12,9) | Adult | 67 | 38,4 | 40 |  | x |  | 8 |
| 10.1001/jama.2020.3204 | Young B et al. | Cross-sectional study | 18 | Singapore | 50,33 (33,78) | Mix | 50 | 100 | 28 |  | x |  | 6 |
| 10.1016/j.ijid.2020.03.040 | Zhang X et al. | Retrospective cohort | 645 | China | 43,12 (13,91) | Mix | 50,85 | 100 | 27,44 |  | x |  | 8 |
| 10.26355/eurrev_202003_20711 | Zheng et al. | Retrospective cohort | 161 | China | 45 (17,5) | Adult | 49,7 | N/A | 20,5 |  | x |  | 7 |
| 10.1016/S0140-6736(20)30566-3 | Zhou et al. | Retrospective cohort | 191 | China | 56 (15,68) | Adult | 119, 62 | 71,27 | 48 | x | x |  | 8 |
| 10.1001/jama.2020.6775 | Richardson et al | Descriptive study | 4,344 | USA | 63.3 | Mix | 60.3 | 93.9 | N/A | x |  |  | 9 |
| 10.1515/dx-2020-0046 | Aggarwal et al . | Retrospective | 16 | USA | 66,75 (16,45) | Adult | 75,00 | 81,25 | N/A |  | x |  | 7 |
| 10.1016/j.gendis.2020.03.008 | Chen et al. | Cross-sectional | 12 | China | 13,50 (1,86) | Pediatric | 50,00 | 100 | N/A |  | x |  | 7 |
| 10.1007/s15010-020-01432-5 | Chen et al. | Retrospective | 145 | China | 47,50 (14,60) | Adult | 54,48 | 100 | N/A |  | x |  | 8 |
| 10.1002/ajh.25774 | Fan et al. | Cross-sectional | 67 | Singapore | 43,25 ( 5,49) | Adult | 55,20 | 100 | N/A |  | x |  | 8 |
| 10.1002/dmrr.3319 | Guo et al. | Retrospective | 174 | China | 58,50 (5,18) | Adult | 43,70 | 94,8 | N/A | x | x |  | 8 |
| 10.1016/j.numecd.2020.04.013 | Li et al. | Retrospective | 83 | China | 45 ( 8,62) | Adult | 41,00 | 92,8 | N/A | x | x |  | 9 |
| 10.1038/s41374-020-0431-6 | Li et al. | Retrospective | 54 | China | 61,8 ( 14,5) | Adult | 63,00 | 88,9 | 55,60 | x |  |  | 7 |
| 10.1007/s11596-020-2176-2 | Li et al. | Retrospective | 25 | China | 48,48 (7,70) | Adult | 48,00 | 80 | N/A | x |  |  | 8 |
| 10.1038/s41430-020-0642-3 | Li et al. | Cross-sectional | 182 | China | 68,50 (8,80) | Adult | 35,71 | N/A | N/A |  | x |  | 9 |
| 10.1016/j.ebiom.2020.102763 | Liu et al. | Retrospective | 40 | China | 48,70 (13,90) | Adult | 37,50 | 92,5 | 35,00 | x | x |  | 8 |
| 10.4158/EP-2020-0108 | Wang F et al | Retrospective | 28 | Wuhan, China | 68,6 (9,0) | Adult | 75,00 | 57,10 | 100,00 | x | x |  | 8 |
| 10.1172/jci.insight.137799 | Wang F et al | Prospective | 65 | Wuhan, China | 57,11 (13,03) | Adult | 57,00 | 92 | N/A | x | x |  | 8 |
| 10.2147/CLEP.S249903 | Wang L et al | Cross-sectional | 26 | Wuhan, China | 42,93 (15,52) | Adult | 42,30 | N/A | N/A |  | x |  | 6 |
| 10.1002/jmv.25930 | Xie J et al | Cross-sectional | 56 | Wuhan, China | 56,83 (11,79) | Adult | 68,00 | N/A | 25,00 |  | x |  | 6 |
| 10.1002/jmv.25944 | Xu T et al | Retrospective | 15 | China | 26,66 (15,54) | Mixed | 66,70 | 100 | 20,00 |  | x |  | 7 |
| 10.1136/bmjdrc-2020-001343 | Yan Y et al | Retrospective | 193 | Wuhan, China | 62 ( 17,93) | Adult | 59,10 | 60 | 48,70 | x | x |  | 8 |
| 10.20452/pamw.15312 | Yao Q et al | Retrospective | 108 | China | 49 (15,78) | Adult | 39,80 | 89 | 23,10 | x |  |  | 8 |
| 10.1016/j.clinthera.2020.04.009 | Yu T et al | Cross-sectional | 95 | China | 38,31 (17,19) | Adult | 55,80 | N/A | N/A |  | x |  | 8 |
| 10.1080/09537104.2020.1754383 | Liu et al. | Retrospective cohort study | 383 | China | 47,0 (20,1) | Adult | 42,30 | 87,2 | N/A |  | x |  | 9 |
| 10.1371/journal.ppat.1008520 | Meng et al. | Retrospective study | 168 | China | 56,7 ( 15,1) | Adult | 51,20 | 86,60 | 33,90 | x | x |  | 9 |
| 10.1016/j.jcrc.2020.04.004 | Piva et al. | single-center cohort study | 33 | Italy | 65 (10,1) | Adult | 93,90 | 97,0 | N/A |  | x |  | 7 |
| 10.1016/j.jaut.2020.102473 | Sun et al. | Retrospective study | 63 | China | 45,5 (17,6) | Mix | 58,70 | 100 | 46 |  | x |  | 8 |
| 10.1016/j.cca.2020.04.024 | Sun et al. | Retrospective study | 116 | China | 49,3 (12,01) | Adult | 51,70 | 100 | N/A |  | x |  | 7 |
| 10.1111/cts.12805 | Zhou et al. | Retrospective study | 21 | China | 66,1 (13,94) | Adult | 61,9 | 85,70 | 76,20 |  | x |  | 6 |
| 10.21037/apm-20-1372 | Liu M et al | Cross sectional | 21 | China | 58 | Adult | 52 | N/A | N/A |  | X |  | 8 |
| 10.1186/s12916-020-01596-9 | Ma H et al | Cross sectional | 50 | China | 2,5 | Pediatric | 56 | N/A | N/A |  | X |  | 8 |
| 10.4103/ijmm.IJMM_20_133 | Liu X et al | Cohort | 303 | China | 70 | Adult | N/A | N/A | N/A |  | X |  | 7 |
| 10.1186/s12939-020-01208-1 | Okoh et al | Cross sectional | 251 | US | 62 | Adult | 51 | N/A | N/A | X | X |  | 8 |
| 10.1002/jmv.26070 | Ni M et al | Cross sectional | 27 | China | 60 | Adult | 51,8 | N/A | N/A | X |  |  | 5 |
| 10.3348/kjr.2020.0171 | Liu Z et al | Cross sectional | 72 | China | 46,2 | Adult | 46,2 | N/A | N/A |  | X |  | 8 |
| 10.1097/INF.0000000000002729 | Lu Y et al | Cross sectional | 81 | China | 4 | Pediatric | 56,8 | N/A | N/A | X | X |  | 7 |

| **Supplementary Table S2. Subgroup analyses for mean hemoglobin levels, g/L** | | | | | |
| --- | --- | --- | --- | --- | --- |
| **Subgroups by study characteristics** | | **Number of studies** | **Mean (95 % CI)** | **^2^I^2^ for heterogeneity** | **^3^P value for heterogeneity (meta-regression)** |
| **^a^Median age, y** | ≤55.25 | 70 | 133.301 131.901 134.700 | 96.9% | **<0.001** |
|  | >55.25 | 69 | 126.133 124.851 127.415 | 97.2% |  |
| **Median percentage of male population, %** | ≤55.1 | 59 | 129.098 127.358 130.839 | 98.7% | 0.34 |
|  | >55.1 | 68 | 130.408 128.750 132.066 | 97.3% |  |
| **Median percentage of population with comorbidities, %^1^** | ≤42.3 | 47 | 130.600 128.607 132.593 | 99.0% | **0.002** |
|  | >42.3 | 38 | 126.684 125.309 128.060 | 91.2% |  |
| **Study Location** | Asia-Pacific | 109 | 130.194 128.826 131.562 | 98.3% | 0.21 |
|  | Middle East | 9 | 128.173 121.195 135.150 | 98.2% |  |
|  | Europe | 12 | 127.232 120.877 133.587 | 97.7% |  |
|  | North America | 9 | 127.626 123.635 131.617 | 98.7% |  |
|  | South America | ---- | ------ | ---- |  |
| **Median percentage of population with cardiovascular diseases, %^1^** | ≤10.6 | 45 | 130.121 127.813 132.429 | 99.1% | 0.16 |
|  | >10.6 | 39 | 127.548 125.549 129.547 | 95.6% |  |
| **Median percentage of population with hypertension, %^1^** | ≤24.7 | 52 | 131.204 129.289 133.120 | 98.8% | **0.003** |
|  | >24.7 | 47 | 126.389 124.668 128.110 | 96.6% |  |
| **Median percentage of population with diabetes, %^1^** | ≤15.5 | 52 | 130.831 128.794 132.869 | 98.7% | **0.013** |
|  | >15.5 | 47 | 126.775 124.880 128.669 | 97.7% |  |
| **Population** | Adults | 116 | 129.025 127.786 130.264 | 98.0% | **0.006** |
|  | Mixed | 19 | 134.375 131.603 137.146 | 97.4% |  |
|  | Pediatric | 4 | 127.402 117.313 137.490 | 94.7% |  |
| **Median percentage of population at ICU, %^1^** | ≤17.55 | 43 | 131.879 129.734 134.025 | 98.6% | **0.04** |
|  | >17.55 | 42 | 129.201 127.695 130.708 | 96.7% |  |
| **Median percentage of population survived at the end of the study, %^1^** | ≤85.85 | 53 | 127.441 125.753 129.129 | 96.9% | 0.07 |
|  | >85.85 | 52 | 132.313 129.837 134.789 | 98.9% |  |
| **Control for overlapping study population** | 20% of studies randomly removed^2^ | 112 | 128.924 127.664 130.184 | 98.0% | n.a |
|  | Chinese studies removed from analysis^3^ | 30 | 129.285 127.089 131.480 | 98% | n.a. |
| ^1^ information was not available for every study  ^2^We randomly removed 20% of studies  3 only studies coming outside of China were included in this analysis | | | | | |

| **Supplementary Table S3. Subgroup analyses difference between hemoglobin levels in severe vs. moderate Covid-19 Cases** | | | | | |
| --- | --- | --- | --- | --- | --- |
| **Subgroups by study characteristics** | | **Number of studies** | **Weighted mean difference (95 % CI)** | **^2^I^2^ for heterogeneity** | **^3^P value for heterogeneity (meta-regression)** |
| **^a^Median age, y** | ≤54.38 | 32 | **-4.661 -6.274 -3.048** | 48.4% | 0.36 |
|  | >54.38 | 30 | **-3.576 -4.986 -2.167** | 67.1% |  |
| **Median percentage of male population, %** | ≤55.2 | 32 | **-4.187 -5.393 -2.980** | 45.0% | 0.69 |
|  | >55.2 | 31 | **-3.880 -5.629 -2.130** | 64.9% |  |
| **Median percentage of population with comorbidities, %^1^** | ≤34.85 | 30 | **-3.466 -4.885 -2.048** | 68.1% | 0.19 |
|  | >34.85 | 10 | **-** **-6.338 -9.137 -3.539** | 0% |  |
| **Study Location** | Asia-Pacific | 50 | **3.665 -4.836 -2.494** | 56.6% | 0.22 |
|  | Middle East | 5 | -5.262 -11.026 0.501 | 55.9% |  |
|  | Europe | 3 | **-9.281 -15.012 -3.550** | 0% |  |
|  | North America | 5 | **-6.312 -7.140 -5.483** | 0% |  |
|  | South America | 0 | ------ | n.a. |  |
| **Median percentage of population with cardiovascular diseases, %^1^** | ≤12 | 15 | -2.451 -4.522 -0.380 | 57.2% | 0.07 |
|  | >12 | 15 | -5.567 -7.831 -3.303 | 39.9% |  |
| **Median percentage of population with hypertension, %^1^** | ≤24.1 | 19 | -4.083 -5.116 -3.050 | 70.7% | **0.006** |
|  | >24.1 | 19 | -4.676 -6.528 -2.824 | 38.8% |  |
| **Median percentage of population with type 2 diabetes, %^1^** | ≤15.86 | 19 | -2.565 -4.839 -0.291 | 56.8% | 0.06 |
|  | >15.85 | 19 | -5.293 -6.807 -3.779 | 41.6% |  |
| **Population** | Adults | 52 | **-4.297 -5.382 -3.212** | 58.7% | 0.73 |
|  | Mixed | 9 | **-3.987 -7.385 -0.588** | 61.4% |  |
|  | Pediatric | 2 | 0.944 -4.441 6.330 | 0% |  |
| **Median percentage of population at ICU, %^1^** | ≤20.7 | 20 | **-3.824 -5.175 -2.473** | 16.2% | 0.15 |
|  | >20.7 | 19 | **-5.643 -7.730 -3.557** | 17.8% |  |
| **Median percentage of population survived at the end of the study, %^1^** | ≤91.75 | 22 | **-4.495 -6.257 -2.733** | 68.6% | 0.55 |
|  | >91.75 | 11 | **-4.434 -5.414 -3.455** | 0% |  |
| **Control for overlapping study population** | 20% of studies randomly removed^2^ | 50 | **-4.053 -5.213 -2.893** | 61.0% | n.a |
|  | Chinese studies removed from analysis^3^ | 19 | **-4.804 -6.408 -3.200** | 47.2% | n.a |
| ^1^ information was not available for every study  ^2^We randomly removed 20 % of studies  ^3^ Only studies coming outside of China were included in this analysis | | | | | |

| **Supplementary Table S4. Subgroup analyses difference between hemoglobin levels in deceased vs. survived Covid-19 Cases** | | | | | |
| --- | --- | --- | --- | --- | --- |
| **Subgroups by study characteristics** | | **Number of studies** | **Weighted mean difference (95 % CI)** | **^2^I^2^ for heterogeneity** | **^3^P value for heterogeneity (meta-regression)** |
| **^a^Median age, y** | ≤62.5 | 14 | **-1.653 -2.940 -0.366** | 0% | 0.28 |
|  | >62.5% | 13 | 0.878 -3.004 4.760 | 84.8% |  |
| **Median percentage of male population, %** | ≤58.05 | 13 | -0.503 -2.118 1.112 | 0% | 0.82 |
|  | >58.05 | 13 | -0.813 -4.564 2.937 | 86.7% |  |
| **Median percentage of population with comorbidities, %^1^** | ≤48.35 | 7 | -1.119 -3.205 0.967 | 0% | 0.35 |
|  | >48.35 | 7 | 1.383 -4.077 6.843 | 88.8% |  |
| **Study Location** | Asia-Pacific | 15 | 1.278 -1.258 3.813 | 69.6% | 0.08 |
|  | Middle East | 4 | -0.202 -5.716 5.311 | 7.1% |  |
|  | Europe | 6 | -3.871 -8.684 0.941 | 64.3% |  |
|  | North America | 2 | **-1.854 -3.623 -0.085** | 0% |  |
|  | South America | 0 | ---- |  |  |
| **Median percentage of population with cardiovascular diseases, %^1^** | ≤12.55 | 10 | -0.073 -4.186 4.039 | 87.4% | **0.96** |
|  | >12.55 | 10 | 0.059 -3.086 3.204 | 22.6% |  |
| **Median percentage of population with hypertension, %^1^** | ≤37.85 | 11 | -1.054 -3.620 1.511 | 37.9% | 0.48 |
|  | >37.85 | 11 | 0.511 -3.212 4.233 | 85.9% |  |
| **Median percentage of population with type 2 diabetes, %^1^** | ≤19 | 12 | -0.312 -2.424 1.799 | 39.9% | 0.95 |
|  | >19 | 10 | -0.281 -4.983 4.421 | 87.0% |  |
| **Population** | Adults | 26 | -0.164 -2.349 2.020 | 74.9% | n.a |
|  | Mixed | 1 | **----** | ---- |  |
|  | Pediatric | 0 | **----** | ---- |  |
| **Median percentage of population at ICU, %^1^** | ≤32 | 8 | 1.179 -1.583 3.940 | 25.3% | 0.35 |
|  | >32 | 8 | -0.931 -2.704 0.842 | 10.1% |  |
| **Median percentage of population survived at the end of the study, %^1^** | ≤71.77 | 12 | 0.814 -2.875 4.502 | 81.1% | 0.54 |
|  | >71.77 | 11 | -0.521 -3.001 1.960 | 48.6% |  |
| **Control for overlapping study population** | 20% of studies randomly removed^2^ | 22 | -1.065 -2.787 0.657 | 49.3% | n.a. |
|  | Chinese studies removed from analysis^3^ | 12 | -2.825 -5.663 0.014 | 58.3% | n.a |
| ^1^ information was not available for every study  ^2^We randomly removed 20 % of studies  ^3^ Only studies coming outside of China were included in this analysis | | | | | |

| **Supplementary Table S5. Subgroup analyses in mean Ferritin levels, ng/mL** | | | | | |
| --- | --- | --- | --- | --- | --- |
| **Subgroups by study characteristics** | | **Number of studies** | **Mean serum total ferritin (95 % CI)** | **^2^I^2^ for heterogeneity** | **^3^P value for heterogeneity (meta-regression)** |
| **^a^Median age, y** | ≤58.6 | 26 | 629.692 521.920 737.465 | 98.7% | **0.008** |
|  | >58.6 | 27 | 915.148 796.791 1033.505 | 99.6% |  |
| **Median percentage of male population, %** | ≤59.75 | 27 | 721.099 613.540 828.657 | 99.5% | 0.34 |
|  | >59.75 | 27 | 831.816 722.799 940.833 | 99.0% |  |
| **Median percentage of population with comorbidities, %^1^** | ≤59.75 | 16 | 670.125 489.631 850.618 | 99.3% | 0.18 |
|  | >59.75 | 14 | 817.690 692.742 942.639 | 99.5% |  |
| **Study Location** | Asia-Pacific | 23 | 618.254 497.956 738.552 | 99.3% | 0.79 |
|  | Middle East | 6 | 552.378 446.427 658.330 | 99.5% |  |
|  | Europe | 10 | 1142.762 895.411 1390.113 | 94.8% |  |
|  | North America | 15 | 975.225 745.919 1204.532 | 99.8% |  |
|  | South America | 0 | ---- |  |  |
| **Median percentage of population with cardiovascular diseases, %^1^** | ≤12.06 | 17 | 686.349 549.445 823.254 | 99.4% | 0.25 |
|  | >12.06 | 16 | 827.797 695.550 960.045 | 99.1% |  |
| **Median percentage of population with hypertension, %^1^** | ≤32.07 | 21 | 628.837 515.683 741.991 | 98.9% | **0.01** |
|  | >32.07 | 18 | 906.365 741.432 1071.298 | 99.7% |  |
| **Median percentage of population with type 2 diabetes, %^1^** | ≤17 | 20 | 653.842 569.481 738.204 | 97.4% | 0.10 |
|  | >17 | 19 | 856.423 700.144 1012.701 | 99.7% |  |
| **Population** | Adults | 51 | 799.467 726.430 872.503 | 99.2% | 0.07 |
|  | Mixed | 2 | 823.098 644.484 1001.713 | 92.0% |  |
|  | Pediatric | 2 | 135.831 -26.379 298.041 | 93.5% |  |
| **Median percentage of population at ICU, %^1^** | ≤26 | 16 | 670.125 489.631 850.618 | 99.3% | 0.19 |
|  | >26 | 17 | 817.690 692.742 942.639 | 99.5% |  |
| **Median percentage of population survived at the end of the study, %^1^** | ≤81.7 | 19 | 1025.026 799.987 1250.065 | 99.6% | **0.002** |
|  | >81.7 | 21 | 608.667 533.083 684.251 | 98.3% |  |
| **Control for overlapping study population** | 20% of studies randomly removed^2^ | 44 | 779.432 691.784 867.079 | 99.5% | n.a |
|  | Chinese studies removed from analysis^3^ | 31 | 925.061 805.875 1044.247 | 99.6% | n.a |
| ^1^ information was not available for every study  ^2^We randomly removed 20 % of studies  ^3^ Only studies coming outside of China were included in this analysis | | | | | |

| **Supplementary Table S6. Subgroup analyses difference between ferritin levels in severe vs. moderate Covid-19 Cases** | | | | | |
| --- | --- | --- | --- | --- | --- |
| **Subgroups by study characteristics** | | **Number of studies** | **Weighted mean difference (95 % CI)** | **^2^I^2^ for heterogeneity** | **^3^P value for heterogeneity (meta-regression)** |
| **Mean serum total ferritin** | | | | | |
| **^a^Median age, y** | ≤57.11 | 14 | **434.568 277.206 591.930** | 72.7% | 0.40 |
|  | >57.11 | 12 | **523.956 394.493 653.419** | 95.6% |  |
| **Median percentage of male population, %** | ≤58.25 | 14 | 476.007 331.397 620.616 | 88.2% | 0.98 |
|  | <58.25 | 3 | 464.022 328.808 599.236 | 94.1% |  |
| **Median percentage of population with comorbidities, %^1^** | ≤42 | 8 | 400.887 224.575 577.198 | 82.4% | 0.07 |
|  | >42 | 8 | 599.703 507.681 691.725 | 66.5% |  |
| **Study Location** | Asia-Pacific | 13 | **556.695 486.436 626.955** | 56.0% | 0.10 |
|  | Middle East | 4 | **245.713 148.006 343.419** | 73.0% |  |
|  | Europe | 5 | **690.487 233.082 1147.892** | 56.3% |  |
|  | North America | 6 | **473.252 382.521 563.983** | 81.8% |  |
|  | South America | ---- | ----- | ----- |  |
| **Median percentage of population with cardiovascular diseases, %^1^** | ≤12.6 | 8 | 509.199 312.055 706.344 | 95.6% | 0.54 |
|  | ≤12.6 | 8 | 406.798 195.307 618.289 | 80.8% |  |
| **Median percentage of population with hypertension, %^1^** | ≤33 | 10 | 411.619 259.345 563.894 | 93.6% | 0.95 |
|  | >33 | 9 | 439.926 302.021 577.832 | 78.0% |  |
| **Median percentage of population with type 2 diabetes, %^1^** | ≤21.75 | 10 | 460.515 310.779 610.251 | 94.3% | 0.66 |
|  | >21.75 | 10 | 420.636 279.790 561.482 | 75.5% |  |
| **Population** | Adults | 26 | 606.369 461.861 750.877 | 90.7% | 0.12 |
|  | Mixed | 2 | **162.761 -107.302 432.825** | 46.6% |  |
|  | Pediatric | 0 | **----** | ---- |  |
| **Median percentage of population at ICU, %^1^** | ≤33 | 8 | **480.944 326.125 635.764** | 88.0% | 0.97 |
|  | >33 | 8 | **495.054 210.662 779.446** | 85.4% |  |
| **Median percentage of population survived at the end of the study, %^1^** | ≤86.7 | 9 | **527.200 282.583 771.817** | 91.3% | 0.13 |
|  | >86.7 | 8 | **393.630 245.971 541.289** | 96.0% |  |
| **Control for overlapping study population** | 20% of studies randomly removed^2^ | 24 | **457.800 350.599 565.001** | 91.1% | n.a |
|  | Chinese studies removed from analysis^3^ | 15 | **337.267 241.486 433.048** | 56.0% | n.a |
| ^1^ information was not available for every study  ^2^We randomly removed 20 % of studies  ^3^ Only studies coming outside of China were included in this analysis | | | | | |

| **Supplementary Table S7. Subgroup analyses difference between ferritin levels in deceased vs. survived Covid-19 Cases** | | | | | |
| --- | --- | --- | --- | --- | --- |
| **Subgroups by study characteristics** | | **Number of studies** | **Weighted mean difference (95 % CI)** | **^2^I^2^ for heterogeneity** | **^3^P value for heterogeneity (meta-regression)** |
| **Mean serum total ferritin,** | | | | | |
| **^a^Median age, y** | ≤61.8 | 9 | **555.360 364.783 745.936** | 91.1% | 0.63 |
|  | >61.8 | 9 | **661.890 440.907 882.874** | 83.1% |  |
| **Median percentage of male population, %** | ≤58.02 | 9 | 567.368 355.985 778.751 | 93.5% | 0.57 |
|  | >58.02 | 9 | 667.612 428.711 906.513 | 84.0% |  |
| **Median percentage of population with comorbidities, %^1^** | ≤29.58 | 5 | 702.482 279.450 1125.513 | 87.8% | 0.48 |
|  | >29.58 | 5 | 506.893 224.813 788.973 | 90.6% |  |
| **Study Location** | Asia-Pacific | 9 | **669.039 430.527 907.550** | 90.7% | 0.52 |
|  | Middle East | 1 | **----** | ---- |  |
|  | Europe | 5 | **511.674 247.812 775.537** | 87.1% |  |
|  | North America | 3 | **561.087 303.572 818.602** | 38.7% |  |
|  | South America | 0 | --- | --- |  |
| **Median percentage of population with cardiovascular diseases, %^1^** | ≤12.35 | 6 | 788.105 367.522 1208.688 | 78.2% | 0.31 |
|  | >12.35 | 6 | 606.369 461.861 750.877 | 94.1% |  |
| **Median percentage of population with hypertension, %^1^** | ≤18.05 | 7 | 611.557 358.534 864.580 | 83.5% | 0.98 |
|  | >18.05 | 6 | 609.972 363.809 856.136 | 93.6% |  |
| **Median percentage of population with type 2 diabetes, %^1^** | ≤39.7 | 7 | 489.841 322.949 656.733 | 87.2% | 0.37 |
|  | >39.7 | 7 | 697.939 457.169 938.710 | 78.0% |  |
| **Population** | Adults |  | 606.369 461.861 750.877 | 90.7% | n.a |
|  | Mixed | 0 | **-----** | ---- |  |
|  | Pediatric | 0 | **----** | ---- |  |
| **Median percentage of population at ICU, %^1^** | ≤29.58 | 6 | **587.557 336.029 839.085** | 92.3% | 0.56 |
|  | >29.58 | 6 | **477.160 253.866 700.455** | 70.9% |  |
| **Median percentage of population survived at the end of the study, %^1^** | ≤86.7 | 8 | **547.125 344.677 749.573** | 73.6% | 0.84 |
|  | >86.7 | 7 | **517.651 310.719 724.582** | 91.3% |  |
| **Control for overlapping study population** | 20% of studies randomly removed^2^ | 9 | **669.039 430.527 907.550** | 90.7% | n.a |
|  | Chinese studies removed from analysis^3^ | 15 | **606.871 448.591 765.150** | 91.2% | n.a |
| ^1^ information was not available for every study  ^2^We randomly removed 20 % of studies  ^3^ Only studies coming outside of China were included in this analysis | | | | | |

| **Supplementary Table S8. Subgroup analyses for mean red blood cell count** | | | | | |
| --- | --- | --- | --- | --- | --- |
| **Subgroups by study characteristics** | | **Number of studies** | **Mean (95 % CI)** | **^2^I^2^ for heterogeneity** | **^3^P value for heterogeneity (meta-regression)** |
| **^a^Median age, y** | ≤57 | 7 | 4.150 3.951 4.349 | 95.8% | **0.64** |
|  | >57 | 6 | 4.013 3.593 4.433 | 98.2% |  |
| **Median percentage of male population, %** | ≤60 | 6 | 4.136 3.869 4.404 | 97.5% | 0.58 |
|  | >60 | 4 | 4.007 3.614 4.400 | 95.0% |  |
| **Median percentage of population with comorbidities, %^1^** | ≤73.2 | 5 | 4.181 3.979 4.383 | 89.6% | **0.04** |
|  | >73.2 | 3 | 3.785 3.718 3.852 | 0.0% |  |
| **Study Location** | Asia-Pacific | 11 | 4.104 3.926 4.282 | 96.2% | 0.76 |
|  | Middle East | 0 | ----- | ---- |  |
|  | Europe | 2 | 4.014 3.004 5.023 | 99.2% |  |
|  | North America | 0 | ---- | ---- |  |
|  | South America | 0 | ---- | ---- |  |
| **Population** | Adults | 12 | 4.051 3.852 4.249 | 97.2% | n.a |
|  | Mixed | 1 | --- | --- |  |
|  | Pediatric | 0 | --- | --- |  |
| **Control for overlapping study population** | 20% of studies randomly removed^2^ | 10 | 4.053 3.835 4.270 | 97.0% | n.a |
|  | Chinese studies removed from analysis^3^ | 11 | 4.104 3.926 4.282 | 96.2% | n.a. |
| ^1^ information was not available for every study  ^2^We randomly removed 20% of studies  3 only studies coming outside of China were included in this analysis | | | | | |

**Supplemental Table S9. Study Quality Assessment**

| **Author-Journal** | **Selection** | **Comparability** | **Outcome** | **Total** | **Comments** |
| --- | --- | --- | --- | --- | --- |
| Chen-JCI (1) | 3 | 1 | 3 | 7 | Retrospective assessment in a single center study, SARS COV2 positive only |
| Chen-Lancet (2) | 3 | 1 | 3 | 7 | Retrospective single center |
| Chen-BMJ (3) | 4 | 1 | 3 | 8 | Single center cohort |
| Cheng-KI (4) | 3 | 2 | 3 | 8 | 3 centers |
| Du-Infection (5) | 4 | 1 | 3 | 8 | 2 centers, restrospective |
| Du-AJRCCM (6) | 3 | 1 | 2 | 6 | 2 centers retrospective, only included mortalities |
| Feng-AJRCCM (7) | 4 | 1 | 3 | 8 | 3 center study restrospective, all spectrum of cases |
| Guan-NEJM (8) | 4 | 2 | 2 | 8 | 552 centers (Largest study?) |
| Hu-SCLF (9) | 3 | 1 | 2 | 6 | Only asymptomatic, restrospective study from screening centers |
| Huang-Lancet (10) | 4 | 1 | 3 | 8 | Outbreak investigation, retrospective study |
| Huang-TMID (11) | 3 | 1 | 2 | 6 | CS study on Editorial Letter format. No details on methods. Single center |
| Jin-Gut (12) | 3 | 2 | 3 | 8 | Few details on the recruitment of participants, restrospective data |
| Lei-TMID (13) | 3 | 1 | 3 | 7 | Cross sectional study, single center |
| Li-IJID (14) | 3 | 1 | 2 | 6 | Single center, only mortalities are included |
| Li-JACI (15) | 4 | 2 | 3 | 9 | Ambispective cohort analysis |
| Lian-CID (16) | 3 | 2 | 3 | 8 | Analysis on elderly vs non-elderly outcomes |
| Liu-JI (17) | 3 | 2 | 3 | 8 | Restrospective analysis, single center, with age stratified analysis |
| Liu-JI (18) | 3 | 2 | 3 | 8 | Single center, restrospective analysis, with analysis on biomarkers and risk factors |
| Pan-AJG (19) | 3 | 2 | 3 | 8 | CS, 3 centers, random selection of 310 patients from participating centers |
| Pan-CTM (20) | 3 | 1 | 3 | 7 | 3 centers, random selection of participants, predictive acuity of routine tests |
| Tan-JCV (21) | 3 | 1 | 2 | 6 | COVID in children, single center, small sample size |
| Wang-JI (22) | 3 | 2 | 3 | 8 | Only included elderly, single center, only in severe |
| Wang-IJID (23) | 3 | 1 | 2 | 6 | Purely descriptive study, |
| Wang-CID (24) | 3 | 1 | 3 | 7 | Single center, only admitted patient |
| Wu-JAMA (25) | 3 | 1 | 3 | 7 | Single center, confirmed cases, Comparison on ARDS/death study |
| Wu-CID (26) | 3 | 1 | 2 | 6 | Multicenter, retrospective, study outside Wuhan |
| Yang-NEJM (27) | 3 | 2 | 3 | 8 | Single center, only critically ill patients |
| Young-JAMA (28) | 3 | 1 | 2 | 6 | Outbreak response in Singapore , purely descriptive study |
| Zhang-IJID (29) | 3 | 2 | 3 | 8 | Focus on radiologic analysis |
| Zheng-ERMPS (30) | 3 | 1 | 3 | 7 | Single center retrospective data collection on confirmed cases |
| Zhou-NEJM (31) | 3 | 2 | 3 | 8 | Retrospective cohort, only adults with confirmed case, 2 centers |
| Wang-EP (32) | 3 | 2 | 3 | 8 | Study limited only to diabetes |
| Wang-CE (33) | 3 | 2 | 3 | 8 | Limited to patient transmission |
| Wang-JCI (34) | 3 | 1 | 2 | 6 | Laboratory assay of 65 COVID patients focusing on immunity |
| Xie-JMV (35) | 3 | 1 | 2 | 6 | Validation of an IgG-IgM test using 56 patients |
| Xu-JMV (36) | 3 | 2 | 2 | 7 | Focus on asymptomatic cases and clinical trajectory |
| Yan-BMJO (37) | 3 | 2 | 3 | 8 | Study on patients with diabetes, single center observational study |
| Yu-CT (38) | 3 | 2 | 3 | 8 | Cross sectional multicenter, to determine risk factors for severe disease |
| Yao-PAIM (39) | 3 | 2 | 3 | 8 | Retrospective review on severe cases |
| Aggarwal (40) | 3 | 1 | 3 | 7 | Single center retrospective data collection on confirmed cases |
| Chen (41) | 3 | 1 | 3 | 7 | Cross-sectional study, limited data |
| Chen (42) | 4 | 1 | 3 | 8 | Retrospective data of 145 patients |
| Fan (43) | 4 | 1 | 3 | 8 | Data of National Centre for Infectious Diseases (NCID) |
| Guo (44) | 3 | 2 | 3 | 8 | Study limited only to diabetes |
| Li (45) | 4 | 2 | 3 | 9 | Study limited only to CVD |
| Li (46) | 3 | 1 | 3 | 7 | Single center retrospective data collection on confirmed cases |
| Li (47) | 3 | 2 | 3 | 8 | Data from a single thoracic department |
| Li (48) | 4 | 2 | 3 | 9 | 182 elderly patients |
| Liu (49) | 3 | 2 | 3 | 8 | Retrospective single center study |
| Sun (50) | 3 | 2 | 2 | 7 | Retrospective study |
| Sun (51) | 3 | 2 | 3 | 8 | Retrospective study |
| Piva (52) | 3 | 2 | 2 | 7 | Single-center observational cohort study |
| Meng (53) | 3 | 3 | 3 | 9 | Retrospective study of severe and critically ill patients |
| Liu (54) | 3 | 3 | 3 | 9 | Retrospective cohort study |
| Zhou (55) | 2 | 2 | 2 | 6 | Retrospective study of severe and critically ill patients |
| Richardson (56) | 4 | 3 | 3 | 10 | Retrospective analysis of sequentially hospitalized patients |
| Aloisio et al (57) | 3 | 2 | 3 | 8 | Retrospective cohort |
| Al-Samkari et al (58) | 3 | 2 | 3 | 8 |  |
| Argenziano et al (59) | 3 | 2 | 3 | 8 | First 1000 patients in a quaternary hospital |
| Asghar et al (60) | 3 | 2 | 3 | 8 | First 100 admitted patients in a tertiary hospital |
| Ayanian et al (61) | 2 | 2 | 2 | 6 |  |
| Bahat et al (62) | 3 | 2 | 3 | 8 | Hemodialysis patients |
| Bao et al (63) | 3 | 2 | 3 | 8 |  |
| Benussi et al (64) | 2 | 2 | 3 | 7 | Patients with concurrent neurologic diseases |
| Bhumbra et al (65) | 2 | 2 | 3 | 7 | Children |
| Bolondi et al (66) | 2 | 2 | 3 | 7 | ICU patients |
| Bonetti et al (67) | 3 | 2 | 3 | 8 |  |
| Borobia et al (68) | 3 | 2 | 3 | 8 |  |
| Masetti et al (69) | 3 | 2 | 3 | 8 |  |
| Wang et al., (70) | 3 | 2 | 3 | 8 | Cross-sectional study |
| Wu et al., (71) | 3 | 2 | 3 | 8 | Cross-sectional |
| Xu H. et al., (72) | 2 | 3 | 2 | 7 | Multicenter retrospective study |
| Xu J. et al., (73) | 3 | 3 | 3 | 9 | Multicenter retrospective study |
| Yang et al. (74) | 3 | 2 | 2 | 7 | Descriptive study |
| Yao et al. (75) | 4 | 3 | 2 | 9 | Case-control |
| Yu et al.(76) | 4 | 3 | 3 | 10 | Rretrospective study |
| Yuan et al., (77) | 3 | 3 | 2 | 8 | Retrospective study |
| Zhang, Wei, Chen, Wan, & Chen, (78) | 4 | 3 | 2 | 9 | Cross-sectional |
| Zhao K. et al. (79) | 4 | 3 | 2 | 9 | Rretrospective study |
| Zhao X. et al., (80) | 3 | 2 | 1 | 6 | Rretrospective study |
| Zhao Z et al. (81) | 2 | 2 | 2 | 6 | Rretrospective study |
| Zhou et al. (82) | 3 | 1 | 2 | 6 | Rretrospective study |
| Duployez N (83) | 2 | 2 | 3 | 7 | Emphasis in Clonal Hematopoiesis |
| Ferguson J (84) | 3 | 1 | 2 | 6 | Multicenter United States positive patients |
| Fu Y. (85) | 3 | 1 | 3 | 7 | Multicenter, retrospective study, Wuhan |
| Gavin et al(86) | 3 | 2 | 3 | 8 | Single center |
| Gayam V et al.(87) | 3 | 2 | 3 | 8 | Single center, African-Americans |
| Giacomelli A (88) | 2 | 2 | 3 | 7 | Multicenter |
| Goicoechea M(89) | 2 | 2 | 3 | 7 | Single-center |
| Guo T (90) | 2 | 2 | 3 | 7 | multicenter study |
| Gupta S(91) | 4 | 2 | 3 | 9 | multicenter cohort |
| Herold T(92) | 4 | 2 | 3 | 9 | Single center |
| Huang JT(93) | 2 | 2 | 3 | 7 |  |
| Huang R(94) | 2 | 2 | 3 | 7 |  |
| Inciardi RM(95) | 2 | 2 | 3 | 7 |  |
| Javanian M (96) | 2 | 2 | 3 | 7 |  |
| Kong M(97) | 2 | 2 | 3 | 7 |  |
| Lagadinou M(98) | 2 | 2 | 3 | 7 |  |
| Gong (99) | 4 | 1 | 3 | 8 | Retrospective three centers study |
| Itelman (100) | 4 | 1 | 3 | 8 | Retrospective tertiary health care unit |
| Ipekci (101) | 3 | 1 | 3 | 7 | One center, small study population |
| Guner (102) | 4 | 1 | 3 | 8 | Retrospective study, 222 patients |
| Gan (103) | 4 | 1 | 3 | 8 | Retrospective Case-Control Study |
| Jiang (104) | 4 | 1 | 3 | 8 | Retrospective three centers study |
| Jang (105) | 4 | 1 | 3 | 8 | Retrospective tertiary health care unit |
| Ghweil (106) | 3 | 1 | 3 | 7 | One center, small study population |
| Kato (107) | 3 | 1 | 3 | 7 | Cross-sectional study, based on Diamond Princess |
| Buckner (108) | 4 | 1 | 2 | 7 | Retrospective chart review |
| Cai (109) | 4 | 1 | 2 | 7 | Retrospective multicenter clinical study |
| Cao (110) | 4 | 1 | 3 | 8 | Retrospective single-center cohort study |
| Cen (111) | 4 | 0 | 2 | 6 | Prospective study |
| Chao (112) | 4 | 1 | 3 | 8 | Retrospective study |
| Chen (113) | 5 | 1 | 3 | 9 | Retrospective study |
| Chen (114) | 4 | 1 | 3 | 8 | Retrospective study |
| Chen (115) | 4 | 1 | 2 | 7 | Single-center retrospective chart review |
| Chen (116) | 5 | 1 | 3 | 9 | Retrospective study |
| Chen (117) | 5 | 1 | 3 | 9 | Retrospective study |
| Chen (118) | 4 | 1 | 3 | 8 | Retrospective study |
| Cheng (119) | 4 | 1 | 3 | 8 | Single-center retrospective chart review |
| Choi (120) | 4 | 1 | 3 | 8 | Single-center retrospective chart review |
| Covino (121) | 4 | 1 | 3 | 8 | Single-center retrospective study |
| Cui (122) | 5 | 1 | 3 | 8 | Multicenter retrospective study |
| Cummings (123) | 5 | 0 | 3 | 8 | Prospective observational study |
| Deng (124) | 4 | 1 | 3 | 8 | Retrospective study |
| Djakpo (125) | 4 | 1 | 3 | 8 | Retrospective study |
| Dong (126) | 3 | 1 | 3 | 7 | Retrospective study |
| Gholizadeh (127) | 3 | 2 | 3 | 8 | Retrospective study of COVID-19 positive patients |
| Pan F (128) | 2 | 2 | 3 | 7 | Retrospective study of COVID-19 positive hospitalized patients |
| Peng J (129) | 3 | 1 | 3 | 7 | Cross-sectional multicenter study focusing on hematologic markers |
| Phipps (130) | 3 | 2 | 3 | 8 | Retrospective cohort focusing on liver markers |
| Piano (131) | 3 | 2 | 2 | 7 | Retrospective cohort focusing on outcomes |
| Popovic (132) | 3 | 1 | 3 | 7 | Retrospective cohort of COVID-19 patients with STEMI |
| Ren (133) | 3 | 2 | 3 | 8 | Retrospective cohort of COVID-19 inpatients |
| Salacup (134) | 3 | 2 | 3 | 8 | Retrospective cohort study of COVID-19 patients |
| Samrah (135) | 3 | 2 | 3 | 8 | Cross sectional study of COVID-19 patients in Jordania |
| Shah (136) | 2 | 1 | 3 | 6 | Retrospective cohort study. DATA ON SERUM IRON AND TRANSFERRIN. |
| Shahriarirad (137) | 3 | 2 | 3 | 8 | Retrospective cohort study of COVID-19 patients in Iran |
| Shang (138) | 3 | 2 | 3 | 8 | Retrospective cohort study of COVID-19 predictive factors for severity |
| Shi (139) | 4 | 2 | 3 | 9 | Retrospective cohort study of COVID-19 patients in the ICU |
| Shi S (140) | 3 | 2 | 3 | 8 | Retrospective cohort study of COVID-19 patients analyzing myocardial injury |
| Shi J (141) | 3 | 2 | 3 | 8 | Prospective cohort study evaluating laboratory markers |
| Singh (142) | 3 | 2 | 3 | 8 | Multicenter retrospective cohort study of COVID-19 patients with preexisting liver disease |
| Smadja (143) | 3 | 2 | 2 | 7 | Retrospective cohort study evaluating the impact of angiopoietin in severity prediction |
| Song (144) | 3 | 2 | 3 | 8 | Retrospective cohort of patients with COVID-19 correlating CT findings and clinical features |
| Song J (145) | 3 | 2 | 3 | 8 | Cross-sectional study evaluating the inflammatory profiles of COVID-19 patients |
| Suleyman (146) | 3 | 2 | 4 | 9 | Retrospective cohort study of risk factors for COVID-19 morbidity |
| Sun H (147) | 3 | 2 | 3 | 8 | Retrospective case-controls study in elderly patients with COVID-19 |
| Sungurtekin (148) | 3 | 2 | 3 | 8 | Retrospective cohort of patients in the ICU |
| Tao (149) | 3 | 2 | 2 | 7 | Retrospective cohort evaluating hematological characteristics of COVID-19 patients |
| Toniati (150) | 3 | 2 | 3 | 8 | Retrospective cohort of COVID-19 patients treated with tocilizumab |
| Usul (151) | 2 | 3 | 3 | 7 | Retrospective cohort comparing COVID-19 positive vs negative individuals |
| Wang (152) | 3 | 2 | 3 | 8 | Retrospective cohort of COVID.19 patients from China |
| Wang F (153) | 3 | 2 | 4 | 9 | Retrospective cohort of COVID-19 patients focused on risk factors |
| Lee J. IJID (154) | 3 | 2 | 3 | 8 |  |
| Li L Thera (155) | 3 | 2 | 3 | 8 |  |
| Li S JCI (156) | 4 | 2 | 3 | 9 | Cohort – follow-up complete |
| Li T AGG (157) | 3 | 2 | 2 | 7 | Cohort incomplete follow-up |
| Li X RID (158) | 3 | 2 | 3 | 8 | Study on radiologic progression of COVID. No comparison between severity or mortality |
| Li Y DIC (159) | 3 | 2 | 3 | 8 | Study on effect of fasting blood glucose. No comparison between severity of mortality |
| Li Y FM (160) | 3 | 2 | 3 | 8 |  |
| Li Y IJID (161) | 3 | 2 | 3 | 8 |  |
| Li Y IJID (162) | 3 | 2 | 3 | 8 |  |
| Lian J Aging (163) | 3 | 2 | 2 | 7 |  |
| Lian J CID (164) | 4 | 2 | 3 | 9 |  |
| Liao D LH (165) | 3 | 2 | 3 | 8 |  |
| Lin Z JI (166) | 2 | 1 | 2 | 5 | Letters to the editor discussing ferritin as an independent risk factor |
| Liu J AIC(167) | 4 | 2 | 3 | 9 |  |
| Liu L FCIM (168) | 3 | 2 | 3 | 8 |  |
| Liu T EMBOMM (169) | 3 | 2 | 3 | 8 |  |
| Liu X PONE (170) | 3 | 2 | 3 | 8 |  |
| Lohse A MI (171) | 2 | 2 | 3 | 7 | Study on the effectiveness of Tocilizumab through restrospective data collection |
| Luo Y IJID (172) | 3 | 2 | 3 | 8 | Development of a risk score, dependent on laboratory values |
| Lu R JIDC (173) | 3 | 2 | 2 | 7 |  |
| Ma Y KIR (174) | 2 | 2 | 3 | 7 | Patients on hemodialysis |
| Maeda T JMV (175) | 3 | 2 | 3 | 8 |  |
| McElvaney O AJRCCM (176) | 3 | 2 | 3 | 8 |  |
| Mikami T JGIM (177) | 4 | 2 | 3 | 9 |  |
| Monfared A TID (178) | 2 | 2 | 3 | 7 | Study focused on renal transplant patients |
| Morrison A JA (179) | 2 | 2 | 3 | 7 |  |
| Nalbant A RAMB (180) | 3 | 2 | 3 | 8 |  |
| Ortiz Brizuela E RIC (181) | 3 | 2 | 2 | 7 | Cohort incomplete follow-up |
| O Reilly E EMA (182) | 3 | 2 | 3 | 8 | Emergency department screening of suspected COVID cases. No comparison between severity and mortality |
| Liu M APM (183) | 3 | 2 | 3 | 8 |  |
| Ma H BMCM (184) | 3 | 2 | 3 | 8 | Focus on pediatric cases |
| Liu X IJMM (185) | 3 | 2 | 2 | 7 |  |
| Okoh A (186) | 3 | 2 | 3 | 8 | Focus on the minority population |
| Ni M JMV (187) | 2 | 1 | 2 | 5 | Mostly cytokine analysis on selected number of patients |
| Liu Z KJR (188) | 3 | 2 | 3 | 8 | Mainly on radiologic characteristics |
| Lu PIDJ (189) | 3 | 1 | 3 | 7 | Focus on pediatric patients |

**References of the included 189 studies**

1. Chen G, Wu D, Guo W, Cao Y, Huang D, Wang H, et al. Clinical and immunologic features in severe and moderate Coronavirus Disease 2019. The Journal of clinical investigation. 2020.

2. Chen N, Zhou M, Dong X, Qu J, Gong F, Han Y, et al. Epidemiological and clinical characteristics of 99 cases of 2019 novel coronavirus pneumonia in Wuhan, China: a descriptive study. The Lancet. 2020;395(10223):507-13.

3. Chen T, Wu D, Chen H, Yan W, Yang D, Chen G, et al. Clinical characteristics of 113 deceased patients with coronavirus disease 2019: retrospective study. BMJ (Clinical research ed). 2020;368:m1091.

4. Cheng Y, Luo R, Wang K, Zhang M, Wang Z, Dong L, et al. Kidney disease is associated with in-hospital death of patients with COVID-19. Kidney International. 2020;20:20.

5. Du W, Yu J, Wang H, Zhang X, Zhang S, Li Q, et al. Clinical characteristics of COVID-19 in children compared with adults in Shandong Province, China. Infection. 2020.

6. Du Y, Tu L, Zhu P, Mu M, Wang R, Yang P, et al. Clinical Features of 85 Fatal Cases of COVID-19 from Wuhan: A Retrospective Observational Study. American journal of respiratory and critical care medicine. 2020.

7. Feng Y, Ling Y, Bai T, Xie Y, Huang J, Li J, et al. COVID-19 with Different Severity: A Multi-center Study of Clinical Features. American journal of respiratory and critical care medicine. 2020.

8. Guan WJ, Ni ZY, Hu Y, Liang WH, Ou CQ, He JX, et al. Clinical Characteristics of Coronavirus Disease 2019 in China. The New England journal of medicine. 2020.

9. Hu Z, Song C, Xu C, Jin G, Chen Y, Xu X, et al. Clinical characteristics of 24 asymptomatic infections with COVID-19 screened among close contacts in Nanjing, China. Science China Life sciences. 2020.

10. Huang C, Wang Y, Li X, Ren L, Zhao J, Hu Y, et al. Clinical features of patients infected with 2019 novel coronavirus in Wuhan, China. The Lancet. 2020;395(10223):497-506.

11. Huang Y, Tu M, Wang S, Chen S, Zhou W, Chen D, et al. Clinical characteristics of laboratory confirmed positive cases of SARS-CoV-2 infection in Wuhan, China: A retrospective single center analysis. Travel Medicine and Infectious Disease. 2020.

12. Jin X, Lian JS, Hu JH, Gao J, Zheng L, Zhang YM, et al. Epidemiological, clinical and virological characteristics of 74 cases of coronavirus-infected disease 2019 (COVID-19) with gastrointestinal symptoms. Gut. 2020.

13. Lei Z, Cao H, Jie Y, Huang Z, Guo X, Chen J, et al. A cross-sectional comparison of epidemiological and clinical features of patients with coronavirus disease (COVID-19) in Wuhan and outside Wuhan, China. Travel Medicine and Infectious Disease. 2020:101664.

14. Li X, Wang L, Yan S, Yang F, Xiang L, Zhu J, et al. Clinical characteristics of 25 death cases with COVID-19: a retrospective review of medical records in a single medical center, Wuhan, China. International journal of infectious diseases : IJID : official publication of the International Society for Infectious Diseases. 2020.

15. Li X, Xu S, Yu M, Wang K, Tao Y, Zhou Y, et al. Risk factors for severity and mortality in adult COVID-19 inpatients in Wuhan. J Allergy Clin Immunol. 2020.

16. Lian J, Jin X, Hao S, Cai H, Zhang S, Zheng L, et al. Analysis of Epidemiological and Clinical features in older patients with Corona Virus Disease 2019 (COVID-19) out of Wuhan. Clinical infectious diseases : an official publication of the Infectious Diseases Society of America. 2020.

17. Liu K, Chen Y, Lin R, Han K. Clinical features of COVID-19 in elderly patients: A comparison with young and middle-aged patients. Journal of Infection. 2020.

18. Liu Y, Du X, Chen J, Jin Y, Peng L, Wang HHX, et al. Neutrophil-to-lymphocyte ratio as an independent risk factor for mortality in hospitalized patients with COVID-19. J Infect. 2020.

19. Pan L, Mu M, Yang P, Sun Y, Wang R, Yan J, et al. Clinical Characteristics of COVID-19 Patients With Digestive Symptoms in Hubei, China: A Descriptive, Cross-Sectional, Multicenter Study. Am J Gastroenterol. 2020.

20. Pan Y, Ye G, Zeng X, Liu G, Zeng X, Jiang X, et al. Can routine laboratory tests discriminate SARS-CoV-2 infected pneumonia from other causes of community acquired pneumonia? Clinical and Translational Medicine. 2020.

21. Tan YP, Tan BY, Pan J, Wu J, Zeng SZ, Wei HY. Epidemiologic and clinical characteristics of 10 children with coronavirus disease 2019 in Changsha, China. Journal of Clinical Virology. 2020;127.

22. Wang L, He W, Yu X, Hu D, Bao M, Liu H, et al. Coronavirus disease 2019 in elderly patients: Characteristics and prognostic factors based on 4-week follow-up. Journal of Infection. 2020.

23. Wang R, Pan M, Zhang X, Fan X, Han M, Zhao F, et al. Epidemiological and clinical features of 125 Hospitalized Patients with COVID-19 in Fuyang, Anhui, China. International journal of infectious diseases : IJID : official publication of the International Society for Infectious Diseases. 2020.

24. Wang Z, Yang B, Li Q, Wen L, Zhang R. Clinical Features of 69 Cases with Coronavirus Disease 2019 in Wuhan, China. Clinical infectious diseases : an official publication of the Infectious Diseases Society of America. 2020.

25. Wu C CC, Cai Y, Xia J, Zhou X, Xu S, et al. Risk Factors Associated With Acute Respiratory Distress Syndrome and Death in Patients With Coronavirus Disease 2019 Pneumonia inWuhan, China. JAMA Internal Medicine. 2020.

26. Wu J, Liu J, Zhao X, Liu C, Wang W, Wang D, et al. Clinical Characteristics of Imported Cases of COVID-19 in Jiangsu Province: A Multicenter Descriptive Study. Clinical infectious diseases : an official publication of the Infectious Diseases Society of America. 2020.

27. Yang X, Yu Y, Xu J, Shu H, Liu H, Wu Y, et al. Clinical course and outcomes of critically ill patients with SARS-CoV-2 pneumonia in Wuhan, China: a single-centered, retrospective, observational study. 2020.

28. Young BE, Ong SWX, Kalimuddin S, Low JG, Tan SY, Loh J, et al. Epidemiologic Features and Clinical Course of Patients Infected with SARS-CoV-2 in Singapore. JAMA - Journal of the American Medical Association. 2020.

29. Zhang X, Cai H, Hu J, Lian J, Gu J, Zhang S, et al. Epidemiological, clinical characteristics of cases of SARS-CoV-2 infection with abnormal imaging findings. International journal of infectious diseases : IJID : official publication of the International Society for Infectious Diseases. 2020.

30. Zheng F, Tang W, Li H, Huang YX, Xie YL, Zhou ZG. Clinical characteristics of 161 cases of corona virus disease 2019 (COVID-19) in Changsha. European Review for Medical and Pharmacological Sciences. 2020;24(6):3404-10.

31. Zhou F, Yu T, Du R, Fan G, Liu Y, Liu Z, et al. Clinical course and risk factors for mortality of adult inpatients with COVID-19 in Wuhan, China: a retrospective cohort study. The Lancet. 2020.

32. Wang F, Yang Y, Dong K, Yan Y, Zhang S, Ren H, et al. Clinical Characteristics of 28 Patients with Diabetes and Covid-19 in Wuhan, China. Endocr Pract. 2020.

33. Wang L, Duan Y, Zhang W, Liang J, Xu J, Zhang Y, et al. Epidemiologic and Clinical Characteristics of 26 Cases of COVID-19 Arising from Patient-to-Patient Transmission in Liaocheng, China. Clin Epidemiol. 2020;12:387-91.

34. Wang F, Hou H, Luo Y, Tang G, Wu S, Huang M, et al. The laboratory tests and host immunity of COVID-19 patients with different severity of illness. JCI Insight. 2020.

35. Xie J, Ding C, Li J, Wang Y, Guo H, Lu Z, et al. Characteristics of patients with coronavirus disease (COVID‐19) confirmed using an IgM‐IgG antibody test. Journal of Medical Virology. 2020.

36. Xu T, Huang R, Zhu L, Wang J, Cheng J, Zhang B, et al. Epidemiological and clinical features of asymptomatic patients with SARS‐CoV‐2 infection. Journal of Medical Virology. 2020.

37. Yan Y, Yang Y, Wang F, Ren H, Zhang S, Shi X, et al. Clinical characteristics and outcomes of patients with severe covid-19 with diabetes. BMJ Open Diabetes Research & Care. 2020;8(1).

38. Yu T, Cai S, Zheng Z, Cai X, Liu Y, Yin S, et al. Association Between Clinical Manifestations and Prognosis in Patients with COVID-19. Clin Ther. 2020.

39. Yao Q, Wang P, Wang X, Qie G, Meng M, Tong X, et al. Retrospective study of risk factors for severe SARS-Cov-2 infections in hospitalized adult patients. Pol Arch Intern Med. 2020.

40. Aggarwal, S., Garcia-Telles, N., Aggarwal, G., Lavie, C., Lippi, G., & Henry, B. (2020). Clinical features, laboratory characteristics, and outcomes of patients hospitalized with coronavirus disease 2019 (COVID-19): Early report from the United States, Diagnosis, 7(2), 91-96.

41. Chen, J., Zhang, Z. Z., Chen, Y. K., Long, Q. X., Tian, W. G., Deng, H. J., ... & Hu, P. (2020). The clinical and immunological features of pediatric COVID-19 patients in China. Genes & Diseases.

42. Chen, Q., Zheng, Z., Zhang, C. et al. Clinical characteristics of 145 patients with corona virus disease 2019 (COVID-19) in Taizhou, Zhejiang, China. Infection (2020).

43. Fan, B. E., Chong, V. C. L., Chan, S. S. W., Lim, G. H., Lim, K. G. E., Tan, G. B., ... & Ong, K. H. (2020). Hematologic parameters in patients with COVID‐19 infection. American journal of hematology.

44. Guo, W., Li, M., Dong, Y., Zhou, H., Zhang, Z., Tian, C., ... & Zhao, L. (2020). Diabetes is a risk factor for the progression and prognosis of COVID‐19. Diabetes/metabolism research and reviews.

45. Li, M., Dong, Y., Wang, H., Guo, W., Zhou, H., Zhang, Z., ... & Zhao, L. (2020). Cardiovascular disease potentially contributes to the progression and poor prognosis of COVID-19. Nutrition, Metabolism and Cardiovascular Diseases.

46. Li, Y., Hu, Y., Yu, J. et al. Retrospective analysis of laboratory testing in 54 patients with severe- or critical-type 2019 novel coronavirus pneumonia. Lab Invest (2020).

47. Li, Y., Peng, S., Li, L. et al. Clinical and Transmission Characteristics of Covid-19 — A Retrospective Study of 25 Cases from a Single Thoracic Surgery Department. CURR MED SCI 40, 295–300 (2020). https://doi.org/10.1007/s11596-020-2176-2

48. Li, T., Zhang, Y., Gong, C. et al. Prevalence of malnutrition and analysis of related factors in elderly patients with COVID-19 in Wuhan, China. Eur J Clin Nutr (2020).

49. Liu, J., Li, S., Liu, J., Liang, B., Wang, X., Wang, H., ... & Xiong, L. (2020). Longitudinal characteristics of lymphocyte responses and cytokine profiles in the peripheral blood of SARS-CoV-2 infected patients. EBioMedicine, 102763.

50. Sun, S., Cai, X., Wang, H., He, G., Lin, Y., Lu, B., ... & Hu, X. (2020). Abnormalities of peripheral blood system in patients with COVID-19 in Wenzhou, China. Clinica Chimica Acta.

51. Sun, Y., Dong, Y., Wang, L., Xie, H., Li, B., Chang, C., & Wang, F. S. (2020). Characteristics and prognostic factors of disease severity in patients with COVID-19: The Beijing experience. Journal of Autoimmunity, 102473.

52. Piva, S., Filippini, M., Turla, F., Catteneo, S., Margola, A., De Fulviis, S., ... & Erbici, G. (2020). Clinical presentation and initial management critically ill patients with severe acute respiratory syndrome coronavirus 2 (SARS-CoV-2) infection in Brescia, Italy. Journal of Critical Care.

53. Meng, Y., Wu, P., Lu, W., Liu, K., Ma, K., Huang, L., ... & Ding, W. (2020). Sex-specific clinical characteristics and prognosis of coronavirus disease-19 infection in Wuhan, China: A retrospective study of 168 severe patients. PLoS pathogens, 16(4), e1008520.

54. Yanli Liu, Wenwu Sun, Yanan Guo, Liangkai Chen, Lijuan Zhang, Su Zhao, Ding Long & Li Yu (2020) Association between platelet parameters and mortality in coronavirus disease 2019: Retrospective cohort study, Platelets, DOI: 10.1080/09537104.2020.1754383

55. Zhou, Y., Han, T., Chen, J., Hou, C., Hua, L., He, S., ... & Zhao, C. (2020). Clinical and Autoimmune Characteristics of Severe and Critical Cases with COVID‐19. Clinical and Translational Science.

56. Richardson, S., Hirsch, J. S., Narasimhan, M., Crawford, J. M., McGinn, T., Davidson, K. W., ... & Cookingham, J. (2020). Presenting characteristics, comorbidities, and outcomes among 5700 patients hospitalized with COVID-19 in the New York City area. Jama.

57. Aloisio E, Chibireva M, Serafini L, Pasqualetti S, Falvella FS, Dolci A, et al. A comprehensive appraisal of laboratory biochemistry tests as major predictors of COVID-19 severity. Arch Pathol Lab Med. 2020.

58. Al-Samkari H, Karp Leaf RS, Dzik WH, Carlson JCT, Fogerty AE, Waheed A, et al. COVID-19 and coagulation: bleeding and thrombotic manifestations of SARS-CoV-2 infection. Blood. 2020;136(4):489-500.

59. Argenziano MG, Bruce SL, Slater CL, Tiao JR, Baldwin MR, Barr RG, et al. Characterization and clinical course of 1000 Patients with COVID-19 in New York: retrospective case series. medRxiv : the preprint server for health sciences. 2020.

60. Asghar MS, Haider Kazmi SJ, Ahmed Khan N, Akram M, Ahmed Khan S, Rasheed U, et al. Clinical Profiles, Characteristics, and Outcomes of the First 100 Admitted COVID-19 Patients in Pakistan: A Single-Center Retrospective Study in a Tertiary Care Hospital of Karachi. Cureus. 2020;12(6):e8712.

61. Ayanian S, Reyes J, Lynn L, Teufel K. The association between biomarkers and clinical outcomes in novel coronavirus pneumonia in a US cohort. Biomark Med. 2020.

62. Aydin Bahat K, Parmaksiz E, Sert S. The clinical characteristics and course of COVID-19 in hemodialysis patients. Hemodialysis international International Symposium on Home Hemodialysis. 2020.

63. Bao C, Tao X, Cui W, Yi B, Pan T, Young KH, et al. SARS-CoV-2 induced thrombocytopenia as an important biomarker significantly correlated with abnormal coagulation function, increased intravascular blood clot risk and mortality in COVID-19 patients. Exp Hematol Oncol. 2020;9(1):16.

64. Benussi A, Pilotto A, Premi E, Libri I, Giunta M, Agosti C, et al. Clinical characteristics and outcomes of inpatients with neurologic disease and COVID-19 in Brescia, Lombardy, Italy. Neurology. 2020.

65. Bhumbra S, Malin S, Kirkpatrick L, Khaitan A, John CC, Rowan CM, et al. Clinical Features of Critical Coronavirus Disease 2019 in Children. Pediatr Crit Care Med. 2020.

66. Bolondi G, Russo E, Gamberini E, Circelli A, Meca MCC, Brogi E, et al. Iron metabolism and lymphocyte characterisation during Covid-19 infection in ICU patients: an observational cohort study. World J Emerg Surg. 2020;15(1):41.

67. Bonetti G, Manelli F, Patroni A, Bettinardi A, Borrelli G, Fiordalisi G, et al. Laboratory predictors of death from coronavirus disease 2019 (COVID-19) in the area of Valcamonica, Italy. Clin Chem Lab Med. 2020;58(7):1100-5.

68. Borobia AM, Carcas AJ, Arnalich F, Alvarez-Sala R, Monserrat-Villatoro J, Quintana M, et al. A Cohort of Patients with COVID-19 in a Major Teaching Hospital in Europe. J Clin Med. 2020;9(6):1-10.

69. Masetti C, Generali E, Colapietro F, Voza A, Cecconi M, Messina A, et al. High mortality in COVID-19 patients with mild respiratory disease. Eur J Clin Invest. 2020:e13314.

70. Wang Y, Liao B, Guo Y, Li F, Lei C, Zhang F, et al. Clinical Characteristics of Patients Infected With the Novel 2019 Coronavirus (SARS-Cov-2) in Guangzhou, China. Open Forum Infect Dis. 2020;7(6):ofaa187.

71. Wu Y, Huang X, Sun J, Xie T, Lei Y, Muhammad J, et al. Clinical Characteristics and Immune Injury Mechanisms in 71 Patients with COVID-19. mSphere. 2020;5(4).

72. Xu H, Liu E, Xie J, Smyth RL, Zhou Q, Zhao R, et al. A follow-up study of children infected with SARS-CoV-2 from western China. Annals of translational medicine. 2020;8(10):623.

73. Xu J, Yang X, Yang L, Zou X, Wang Y, Wu Y, et al. Clinical course and predictors of 60-day mortality in 239 critically ill patients with COVID-19: a multicenter retrospective study from Wuhan, China. Critical care (London, England). 2020;24(1):394.

74. Yang L, Liu J, Zhang R, Li M, Li Z, Zhou X, et al. Epidemiological and clinical features of 200 hospitalized patients with corona virus disease 2019 outside Wuhan, China: A descriptive study. J Clin Virol. 2020;129:104475.

75. Yao Y, Cao J, Wang Q, Shi Q, Liu K, Luo Z, et al. D-dimer as a biomarker for disease severity and mortality in COVID-19 patients: a case control study. J Intensive Care. 2020;8(1):49.

76. Yu C, Lei Q, Li W, Wang X, Li W, Liu W. Epidemiological and clinical characteristics of 1663 hospitalized patients infected with COVID-19 in Wuhan, China: a single-center experience. J Infect Public Health. 2020.

77. Yuan X, Huang W, Ye B, Chen C, Huang R, Wu F, et al. Changes of hematological and immunological parameters in COVID-19 patients. Int J Hematol. 2020.

78. Zhang Q, Wei Y, Chen M, Wan Q, Chen X. Clinical analysis of risk factors for severe COVID-19 patients with type 2 diabetes. Journal of diabetes and its complications. 2020:107666.

79. Zhao K, Huang J, Dai D, Feng Y, Liu L, Nie S. Serum Iron Level as a Potential Predictor of Coronavirus Disease 2019 Severity and Mortality: A Retrospective Study. Open Forum Infect Dis. 2020;7(7):ofaa250.

80. Zhao X, Li Y, Ge Y, Shi Y, Lv P, Zhang J, et al. Evaluation of Nutrition Risk and Its Association With Mortality Risk in Severely and Critically Ill COVID-19 Patients. JPEN J Parenter Enteral Nutr. 2020.

81. Zhao Z, Chen A, Hou W, Graham JM, Li H, Richman PS, et al. Prediction model and risk scores of ICU admission and mortality in COVID-19. PLoS One. 2020;15(7):e0236618.

82. Zhou Y, Han T, Chen J, Hou C, Hua L, He S, et al. Clinical and Autoimmune Characteristics of Severe and Critical Cases of COVID-19. Clinical and translational science. 2020.

83. Duployez N, Demonchy J, Berthon C, Goutay J, Caplan M, Moreau AS, et al. Clinico-Biological Features and Clonal Hematopoiesis in Patients with Severe COVID-19. Cancers (Basel). 2020;12(7):1-11.

84. Ferguson J, Rosser JI, Quintero O, Scott J, Subramanian A, Gumma M, et al. Characteristics and Outcomes of Coronavirus Disease Patients under Nonsurge Conditions, Northern California, USA, March-April 2020. Emerging infectious diseases. 2020;26(8):1679-85.

85. Fu Y, Zhu R, Bai T, Han P, He Q, Jing M, et al. Clinical Features of COVID-19-Infected Patients With Elevated Liver Biochemistries: A Multicenter, Retrospective Study. Hepatology. 2020.

86. Gavin W, Campbell E, Zaidi SA, Gavin N, Dbeibo L, Beeler C, et al. Clinical characteristics, outcomes and prognosticators in adult patients hospitalized with COVID-19. Am J Infect Control. 2020.

87. Gayam V, Chobufo MD, Merghani MA, Lamichanne S, Garlapati PR, Adler MK. Clinical characteristics and predictors of mortality in African-Americans with COVID-19 from an inner-city community teaching hospital in New York. J Med Virol. 2020.

88. Giacomelli A, Ridolfo AL, Milazzo L, Oreni L, Bernacchia D, Siano M, et al. 30-day mortality in patients hospitalized with COVID-19 during the first wave of the Italian epidemic: A prospective cohort study. Pharmacol Res. 2020;158:104931.

89. Goicoechea M, Sanchez Camara LA, Macias N, Munoz de Morales A, Rojas AG, Bascunana A, et al. COVID-19: clinical course and outcomes of 36 hemodialysis patients in Spain. Kidney Int. 2020;98(1):27-34.

90. Guo T, Shen Q, Guo W, He W, Li J, Zhang Y, et al. Clinical Characteristics of Elderly Patients with COVID-19 in Hunan Province, China: A Multicenter, Retrospective Study. Gerontology. 2020:1-9.

91. Gupta S, Hayek SS, Wang W, Chan L, Mathews KS, Melamed ML, et al. Factors Associated With Death in Critically Ill Patients With Coronavirus Disease 2019 in the US. JAMA internal medicine. 2020.

92. Herold T, Jurinovic V, Arnreich C, Lipworth BJ, Hellmuth JC, von Bergwelt-Baildon M, et al. Elevated levels of IL-6 and CRP predict the need for mechanical ventilation in COVID-19. J Allergy Clin Immunol. 2020;146(1):128-36 e4.

93. Huang JT, Ran RX, Lv ZH, Feng LN, Ran CY, Tong YQ, et al. Chronological Changes of Viral Shedding in Adult Inpatients with COVID-19 in Wuhan, China. Clin Infect Dis. 2020.

94. Huang R, Zhu L, Xue L, Liu L, Yan X, Wang J, et al. Clinical findings of patients with coronavirus disease 2019 in Jiangsu province, China: A retrospective, multi-center study. PLoS Negl Trop Dis. 2020;14(5):e0008280.

95. Inciardi RM, Adamo M, Lupi L, Cani DS, Di Pasquale M, Tomasoni D, et al. Characteristics and outcomes of patients hospitalized for COVID-19 and cardiac disease in Northern Italy. European heart journal. 2020;41(19):1821-9.

96. Javanian M, Bayani M, Shokri M, Sadeghi-Haddad-Zavareh M, Babazadeh A, Yeganeh B, et al. Clinical and laboratory findings from patients with COVID-19 pneumonia in Babol North of Iran: a retrospective cohort study. Rom J Intern Med. 2020.

97. Kong M, Zhang H, Cao X, Mao X, Lu Z. Higher level of neutrophil-to-lymphocyte is associated with severe COVID-19. Epidemiol Infect. 2020;148:e139.

98. Lagadinou M, Salomou EE, Zareifopoulos N, Marangos M, Gogos C, Velissaris D. Prognosis of COVID-19: Changes in laboratory parameters. Infez Med. 2020;28(suppl 1):89-95.

99. Gan J, Li J, Li S, Yang C. Leucocyte Subsets Effectively Predict the Clinical Outcome of Patients With COVID-19 Pneumonia: A Retrospective Case-Control Study. Frontiers in public health. 2020;8:299.

100. Ghweil AA, Hassan MH, Mohamed AK, Mohamed AO, Mohammed HM, Abdelazez AA, et al. <p>Characteristics, Outcomes and Indicators of Severity for COVID-19 Among Sample of ESNA Quarantine Hospital’s Patients, Egypt: A Retrospective Study</p>. Infection and Drug Resistance. 2020;Volume 13:2375-83.

101. Gong J, Ou J, Qiu X, Jie Y, Chen Y, Yuan L, et al. A Tool for Early Prediction of Severe Coronavirus Disease 2019 (COVID-19): A Multicenter Study Using the Risk Nomogram in Wuhan and Guangdong, China. Clinical infectious diseases : an official publication of the Infectious Diseases Society of America. 2020;71(15):833-40.

102. Guner R, Hasanoglu I, Kayaaslan B, Aypak A, Kaya Kalem A, Eser F, et al. COVID-19 experience of the major pandemic response center in the capital: Results of the pandemic's first month in Turkey. Turk J Med Sci. 2020.

103. Ipekci A, Akdeniz YS, Tutar O, Sirolu S, Simsek O, Ozkan S. The Clinical and Computed Tomography Findings of Patients with COVID-19. Signa Vitae. 2020;16(1):173-8.

104. Itelman E, Wasserstrum Y, Segev A, Avaky C, Negru L, Cohen D, et al. Clinical Characterization of 162 COVID-19 patients in Israel: Preliminary Report from a Large Tertiary Center. Isr Med Assoc J. 2020;22(5):271-4.

105. Jang JG, Hur J, Choi EY, Hong KS, Lee W, Ahn JH. Prognostic Factors for Severe Coronavirus Disease 2019 in Daegu, Korea. Journal of Korean medical science. 2020;35(23):e209.

106. Jiang S, Wang R, Li L, Hong D, Ru R, Rao Y, et al. Liver Injury in Critically Ill and Non-critically Ill COVID-19 Patients: A Multicenter, Retrospective, Observational Study. Front Med (Lausanne). 2020;7:347.

107. Kato H, Shimizu H, Shibue Y, Hosoda T, Iwabuchi K, Nagamine K, et al. Clinical course of 2019 novel coronavirus disease (COVID-19) in individuals present during the outbreak on the Diamond Princess cruise ship. Journal of infection and chemotherapy : official journal of the Japan Society of Chemotherapy. 2020;26(8):865-9.

108. Buckner FS, McCulloch DJ, Atluri V, Blain M, McGuffin SA, Nalla AK, et al. Clinical Features and Outcomes of 105 Hospitalized patients with COVID-19 in Seattle, Washington. Clinical infectious diseases : an official publication of the Infectious Diseases Society of America. 2020.

109. Cai SH, Liao W, Chen SW, Liu LL, Liu SY, Zheng ZD. Association between obesity and clinical prognosis in patients infected with SARS-CoV-2. Infectious Diseases of Poverty. 2020;9(1).

110. Cao Z, Li T, Liang L, Wang H, Wei F, Meng S, et al. Clinical characteristics of Coronavirus Disease 2019 patients in Beijing, China. PLoS ONE. 2020;15(6).

111. Cen Y, Chen X, Shen Y, Zhang XH, Lei Y, Xu C, et al. Risk factors for disease progression in patients with mild to moderate coronavirus disease 2019—a multi-centre observational study. Clinical Microbiology and Infection. 2020.

112. Chao JY, Derespina KR, Herold BC, Goldman DL, Aldrich M, Weingarten J, et al. Clinical Characteristics and Outcomes of Hospitalized and Critically Ill Children and Adolescents with Coronavirus Disease 2019 at a Tertiary Care Medical Center in New York City. Journal of Pediatrics. 2020;223:14-9.e2.

113. Chen FF, Zhong M, Liu Y, Zhang Y, Zhang K, Su DZ, et al. The characteristics and outcomes of 681 severe cases with COVID-19 in China. Journal of Critical Care. 2020;60:32-7.

114. Chen R, Sang L, Jiang M, Yang Z, Jia N, Fu W, et al. Longitudinal hematologic and immunologic variations associated with the progression of COVID-19 patients in China. Journal of Allergy and Clinical Immunology. 2020.

115. Chen TY, Farghaly S, Cham S, Tatem LL, Sin JH, Rauda R, et al. COVID-19 pneumonia in kidney transplant recipients: Focus on immunosuppression management. Transplant infectious disease : an official journal of the Transplantation Society. 2020:e13378.

116. Chen W, Li Z, Yang B, Wang P, Zhou Q, Zhang Z, et al. Delayed-phase thrombocytopenia in patients with coronavirus disease 2019 (COVID-19). British Journal of Haematology. 2020;190(2):179-84.

117. Chen X, Zhu B, Hong W, Zeng J, He X, Chen J, et al. Associations of Clinical Characteristics and Treatment Regimens with Viral RNA Shedding Duration in Patients with COVID-19. International journal of infectious diseases : IJID : official publication of the International Society for Infectious Diseases. 2020.

118. Chen Y, Yang D, Cheng B, Chen J, Peng A, Yang C, et al. Clinical Characteristics and Outcomes of Patients With Diabetes and COVID-19 in Association With Glucose-Lowering Medication. Diabetes care. 2020;43(7):1399-407.

119. Cheng A, Hu L, Wang Y, Huang L, Zhao L, Zhang C, et al. Diagnostic performance of initial blood urea nitrogen combined with D-Dimer levels for predicting in-hospital mortality in COVID-19 patients. International journal of antimicrobial agents. 2020:106110.

120. Choi MH, Ahn H, Ryu HS, Kim BJ, Jang J, Jung M, et al. Clinical characteristics and disease progression in early-stage covid-19 patients in south korea. Journal of Clinical Medicine. 2020;9(6):1-19.

121. Covino M, De Matteis G, Santoro M, Sabia L, Simeoni B, Candelli M, et al. Clinical characteristics and prognostic factors in COVID-19 patients aged ≥80 years. Geriatrics and Gerontology International. 2020;20(7):704-8.

122. Cui X, Yu X, Wu X, Huang L, Tian Y, Huang X, et al. Acute Kidney Injury in Patients with the Coronavirus Disease 2019: A Multicenter Study. Kidney & blood pressure research. 2020;45(4):612-22.

123. Cummings MJ, Baldwin MR, Abrams D, Jacobson SD, Meyer BJ, Balough EM, et al. Epidemiology, clinical course, and outcomes of critically ill adults with COVID-19 in New York City: a prospective cohort study. The Lancet. 2020;395(10239):1763-70.

124. Deng P, Ke Z, Ying B, Qiao B, Yuan L. The diagnostic and prognostic role of myocardial injury biomarkers in hospitalized patients with COVID-19. Clinica Chimica Acta. 2020;510:186-90.

125. Djakpo DK, Wang Z, Zhang R, Chen X, Chen P, Antoine M. Blood routine test in mild and common 2019 coronavirus (COVID-19) patients. Bioscience reports. 2020.

126. Dong X, Wang M, Liu S, Zhu J, Xu Y, Cao H, et al. Immune characteristics of patients with coronavirus disease 2019 (COVID-19). Aging and Disease. 2020;11(3):642-8.

127. Pan F, Yang L, Li Y, Liang B, Li L, Ye T, et al. Factors associated with death outcome in patients with severe coronavirus disease-19 (Covid-19): A case-control study. International Journal of Medical Sciences. 2020;17(9):1281-92.

128. Peng J, Qi D, Yuan G, Deng X, Mei Y, Feng L, et al. Diagnostic value of peripheral hematologic markers for coronavirus disease 2019 (COVID-19): A multicenter, cross-sectional study. Journal of Clinical Laboratory Analysis. 2020.

129. Phipps MM, Barraza LH, LaSota ED, Sobieszczyk ME, Pereira MR, Zheng EX, et al. Acute Liver Injury in COVID-19: Prevalence and Association with Clinical Outcomes in a Large US Cohort. Hepatology (Baltimore, Md). 2020.

130. Piano S, Dalbeni A, Vettore E, Benfaremo D, Mattioli M, Gambino CG, et al. Abnormal liver function tests predict transfer to intensive care unit and death in COVID-19. Liver International. 2020.

131. Popovic B, Varlot J, Metzdorf PA, Jeulin H, Goehringer F, Camenzind E. Changes in characteristics and management among patients with ST-elevation myocardial infarction due to COVID-19 infection. Catheterization and Cardiovascular Interventions. 2020.

132. Ren H, Yang Y, Wang F, Yan Y, Shi X, Dong K, et al. Association of the insulin resistance marker TyG index with the severity and mortality of COVID-19. Cardiovascular diabetology. 2020;19(1):58.

133. Safari R, Gholizadeh P, Marofi P, Zeinalzadeh E, Pagliano P, Ganbarov K, et al. Alteration of liver biomarkers in patients with SARS-CoV-2 (COVID-19). Journal of Inflammation Research. 2020;13:285-92.

134. Salacup G, Lo KB, Gul F, Peterson E, De Joy R, Bhargav R, et al. Characteristics and clinical outcomes of COVID-19 patients in an underserved-inner city population: A single tertiary center cohort. Journal of Medical Virology. 2020.

135. Samrah SM, A.-H WA-M, Ibnian AM, Raffee LA, Momany SM, Al-Ali M, et al. COVID-19 outbreak in Jordan: Epidemiological features, clinical characteristics, and laboratory findings. Annals of Medicine and Surgery. 2020;57:103-8.

136. Shah A, Frost J, Aaron L, Donovan K, ... Systemic hypoferremia and severity of hypoxemic respiratory failure in COVID-19: ccforum.biomedcentral.com; 2020.

137. Shahriarirad R, Shahriarirad R, Khodamoradi Z, Khodamoradi Z, Erfani A, Erfani A, et al. Epidemiological and clinical features of 2019 novel coronavirus diseases (COVID-19) in the South of Iran. BMC Infectious Diseases. 2020;20(1).

138. Shang W, Dong J, Ren Y, Tian M, Li W, Hu J, et al. The value of clinical parameters in predicting the severity of COVID-19. Journal of Medical Virology. 2020.

139. Shi J, Li Y, Zhou X, Zhang Q, Ye X, Wu Z, et al. Lactate dehydrogenase and susceptibility to deterioration of mild COVID-19 patients: A multicenter nested case-control study. BMC Medicine. 2020;18(1).

140. Shi M, Chen L, Yang Y, Zhang J, Xu J, Xu G, et al. Analysis of clinical features and outcomes of 161 patients with severe and critical COVID-19: A multicenter descriptive study. Journal of Clinical Laboratory Analysis. 2020.

141. Shi S, Qin M, Cai Y, Liu T, Shen B, Yang F, et al. Characteristics and clinical significance of myocardial injury in patients with severe coronavirus disease 2019. European heart journal. 2020;41(22):2070-9.

142. Singh S, Khan A. Clinical Characteristics and Outcomes of Coronavirus Disease 2019 Among Patients With Preexisting Liver Disease in the United States: A Multicenter Research Network Study. Gastroenterology. 2020.

143. Smadja DM, Guerin CL, Chocron R, Yatim N, Boussier J, Gendron N, et al. Angiopoietin-2 as a marker of endothelial activation is a good predictor factor for intensive care unit admission of COVID-19 patients. Angiogenesis. 2020.

144. Song JW, Zhang C, Fan X, Meng FP, Xu Z, Xia P, et al. Immunological and inflammatory profiles in mild and severe cases of COVID-19. Nature communications. 2020;11(1):3410.

145. Song S, Wu F, Liu Y, Jiang H, Xiong F, Guo X, et al. Correlation between Chest CT Findings and Clinical Features of 211 COVID-19 Suspected Patients in Wuhan, China. Open Forum Infectious Diseases. 2020;7(6).

146. Suleyman G, Fadel RA, Malette KM, Hammond C, Abdulla H, Entz A, et al. Clinical Characteristics and Morbidity Associated with Coronavirus Disease 2019 in a Series of Patients in Metropolitan Detroit. JAMA Network Open. 2020.

147. Sun H, Ning R, Tao Y, Yu C, Deng X, Zhao C, et al. Risk Factors for Mortality in 244 Older Adults With COVID-19 in Wuhan, China: A Retrospective Study. Journal of the American Geriatrics Society. 2020;68(6):E19-E23.

148. Sungurtekin H, Arslan Ü, Özgen C, Akbudak İ H, Kahramanoglu M, Erbay H, et al. Prognosis of COVID-19 patients requiring intensive care unit care. Signa Vitae. 2020;16(1):147-51.

149. Tao Z, Liu M, Wu J, Xu J, Chen W, Yang Z, et al. Anaemia is Associated with Severe Illness in COVID-19: A Retrospective Cohort Study: researchsquare.com; 2020.

150. Toniati P, Piva S, Cattalini M, Garrafa E, Regola F, ... Tocilizumab for the treatment of severe COVID-19 pneumonia with hyperinflammatory syndrome and acute respiratory failure: A single center study of 100 …: Elsevier; 2020.

151. Usul E, Şan İ, Bekgöz B, Şahin A. The role of hematological parameters in COVID-19 patients in the emergency room. Biomarkers in medicine. 2020.

152. Wang C, Deng R, Gou L, Fu Z, Zhang X, Shao F, et al. Preliminary study to identify severe from moderate cases of COVID-19 using combined hematology parameters. Annals of translational medicine. 2020;8(9):593.

153. Wang F, Qu M, Zhou X, Zhao K, Lai C, Tang Q, et al. The timeline and risk factors of clinical progression of COVID-19 in Shenzhen, China. Journal of Translational Medicine. 2020;18(1).

154. Lee JY, Hong SW, Hyun M, Park JS, Lee JH, Suh YS, et al. Epidemiological and Clinical Characteristics of Coronavirus Disease 2019 in Daegu, South Korea. Int J Infect Dis. 2020.

155. Li L, Yang L, Gui S, Pan F, Ye T, Liang B, et al. Association of clinical and radiographic findings with the outcomes of 93 patients with COVID-19 in Wuhan, China. Theranostics. 2020;10(14):6113-21.

156. Li S, Jiang L, Li X, Lin F, Wang Y, Li B, et al. Clinical and pathological investigation of patients with severe COVID-19. JCI Insight. 2020;5(12).

157. Li T, Lu L, Zhang W, Tao Y, Wang L, Bao J, et al. Clinical characteristics of 312 hospitalized older patients with COVID-19 in Wuhan, China. Arch Gerontol Geriatr. 2020;91:104185.

158. Li X, Pan Z, Xia Z, Li R, Wang X, Zhang R, et al. Clinical and CT characteristics which indicate timely radiological reexamination in patients with COVID-19: A retrospective study in Beijing, China. Radiology of Infectious Diseases. 2020.

159. Li Y, Han X, Alwalid O, Cui Y, Cao Y, Liu J, et al. Baseline characteristics and risk factors for short-term outcomes in 132 COVID-19 patients with diabetes in Wuhan China: A retrospective study. Diabetes research and clinical practice. 2020;166:108299.

160. Li Y, Shi J, Xia J, Duan J, Chen L, Yu X, et al. Asymptomatic and Symptomatic Patients With Non-severe Coronavirus Disease (COVID-19) Have Similar Clinical Features and Virological Courses: A Retrospective Single Center Study. Frontiers in Microbiology. 2020;11.

161. Li Y, Wang H, Wang F, Du H, Liu X, Chen P, et al. Comparison of hospitalized patients with pneumonia caused by COVID-19 and influenza A in children under 5 years. Int J Infect Dis. 2020;98:80-3.

162. Li Y, Wang J, Wang C, Yang Q, Xu Y, Xu J, et al. Characteristics of respiratory virus infection during the outbreak of 2019 novel coronavirus in Beijing. Int J Infect Dis. 2020;96:266-9.

163. Lian J, Jin C, Hao S, Zhang X, Yang M, Jin X, et al. High neutrophil-to-lymphocyte ratio associated with progression to critical illness in older patients with COVID-19: a multicenter retrospective study. Aging. 2020;12.

164. Lian J, Jin X, Hao S, Cai H, Zhang S, Zheng L, et al. Analysis of Epidemiological and Clinical Features in Older Patients With Coronavirus Disease 2019 (COVID-19) Outside Wuhan. Clinical infectious diseases : an official publication of the Infectious Diseases Society of America. 2020;71(15):740-7.

165. Liao D, Zhou F, Luo L, Xu M, Wang H, Xia J, et al. Haematological characteristics and risk factors in the classification and prognosis evaluation of COVID-19: a retrospective cohort study. The Lancet Haematology. 2020.

166. Lin Z, Long F, Yang Y, Chen X, Xu L, ... Serum ferritin as an independent risk factor for severity in COVID-19 patients. Journal of Infection. 2020.

167. Liu J, Zhang S, Wu Z, Shang Y, Dong X, Li G, et al. Clinical outcomes of COVID-19 in Wuhan, China: a large cohort study. Annals of intensive care. 2020;10(1):99.

168. Liu L, Lei X, Xiao X, Yang J, Li J, Ji M, et al. Epidemiological and Clinical Characteristics of Patients With Coronavirus Disease-2019 in Shiyan City, China. Front Cell Infect Microbiol. 2020;10:284.

169. Liu T, Zhang J, Yang Y, Ma H, Li Z, Zhang J, et al. The role of interleukin-6 in monitoring severe case of coronavirus disease 2019. EMBO Mol Med. 2020;12(7):e12421.

170. Liu X, Yue X, Liu F, Wei L, Chu Y, Bao H, et al. Analysis of clinical features and early warning signs in patients with severe COVID-19: A retrospective cohort study. PLoS One. 2020;15(6):e0235459.

171. Lohse A, Klopfenstein T, Balblanc JC, Royer PY, Bossert M, Gendrin V, et al. Predictive factors of mortality in patients treated with tocilizumab for acute respiratory distress syndrome related to coronavirus disease 2019 (COVID-19). Microbes Infect. 2020.

172. Lu R, Qin J, Wu Y, Wang J, Huang S, Tian L, et al. Epidemiological and clinical characteristics of COVID-19 patients in Nantong, China. Journal of infection in developing countries. 2020;14(5):440-6.

173. Luo Y, Yuan X, Xue Y, Mao L, Lin Q, Tang G, et al. Using a diagnostic model based on routine laboratory tests to distinguish patients infected with SARS-CoV-2 from those infected with influenza virus. Int J Infect Dis. 2020;95:436-40.

174. Ma Y, Diao B, Lv X, Zhu J, Chen C, Liu L, et al. Epidemiological, Clinical, and Immunological Features of a Cluster of COVID-19–Contracted Hemodialysis Patients. Kidney International Reports. 2020;5(8):1333-41.

175. Maeda T, Obata R, Rizk DD, Kuno T. The Association of Interleukin-6 value, Interleukin inhibitors and Outcomes of Patients with COVID-19 in New York City. Journal of medical virology. 2020.

176. McElvaney OJ, McEvoy N, McElvaney OF, Carroll TP, Murphy MP, Dunlea DM, et al. Characterization of the Inflammatory Response to Severe COVID-19 Illness. American journal of respiratory and critical care medicine. 2020.

177. Mikami T, Miyashita H, Yamada T, Harrington M, Steinberg D, Dunn A, et al. Risk Factors for Mortality in Patients with COVID-19 in New York City. J Gen Intern Med. 2020.

178. Monfared A, Dashti-Khavidaki S, Jafari R, Jafari A, Ramezanzade E, Lebadi MK, et al. Clinical characteristics and outcome of COVID-19 pneumonia in kidney transplant recipients in Razi hospital, Rasht, Iran. Transpl Infect Dis. 2020:e13420.

179. Morrison AR, Johnson JM, Griebe KM, Jones MC, Stine JJ, Hencken LN, et al. Clinical characteristics and predictors of survival in adults with coronavirus disease 2019 receiving tocilizumab. J Autoimmun. 2020:102512.

180. Nalbant A, Kaya T, Varim C, Yaylaci S, Tamer A, Cinemre H. Can the neutrophil/lymphocyte ratio (NLR) have a role in the diagnosis of coronavirus 2019 disease (COVID-19)? Revista da Associacao Medica Brasileira (1992). 2020;66(6):746-51.

181. O'Reilly GM, Mitchell RD, Wu J, Rajiv P, Bannon-Murphy H, Amos T, et al. Epidemiology and clinical features of emergency department patients with suspected COVID-19: Results from the first month of the COVID-19 Emergency Department Quality Improvement Project (COVED-2). Emerg Med Australas. 2020.

182. Ortiz-Brizuela E, Villanueva-Reza M, Gonzalez-Lara MF, Tamez-Torres KM, Roman-Montes CM, Diaz-Mejia BA, et al. Clinical and Epidemiological Characteristics of Patients Diagnosed with Covid-19 in a Tertiary Care Center in Mexico City: A Prospective Cohort Study. Rev Invest Clin. 2020;72(3):165-77.

183. Liu M, Lyu Y, Zhao W, Yu S, Shi D, Lu W. Case analysis of novel coronavirus pneumonia in the Second Hospital of Wuhan Iron and Steel Company, Qingshan District, Wuhan, China. Ann Palliat Med. 2020.

184. Liu X, Lv J, Gan L, Zhang Y, Sun F, Meng B, et al. Comparative analysis of clinical characteristics, imaging and laboratory findings of different age groups with COVID-19. Indian journal of medical microbiology. 2020;38(1):87-93.

185. Liu Z, Jin C, Wu CC, Liang T, Zhao H, Wang Y, et al. Association between Initial Chest CT or Clinical Features and Clinical Course in Patients with Coronavirus Disease 2019 Pneumonia. Korean J Radiol. 2020;21(6):736-45.

186. Lu Y, Li Y, Deng W, Liu M, He Y, Huang L, et al. Symptomatic Infection is Associated with Prolonged Duration of Viral Shedding in Mild Coronavirus Disease 2019: A Retrospective Study of 110 Children in Wuhan. Pediatr Infect Dis J. 2020;39(7):e95-e9.

187. Ma H, Hu J, Tian J, Zhou X, Li H, Laws MT, et al. A single-center, retrospective study of COVID-19 features in children: a descriptive investigation. BMC Med. 2020;18(1):123.

188. Ni M, Tian FB, Xiang DD, Yu B. Characteristics of inflammatory factors and lymphocyte subsets in patients with severe COVID-19. Journal of medical virology. 2020.

189. Okoh AK, Sossou C, Dangayach NS, Meledathu S, Phillips O, Raczek C, et al. Coronavirus disease 19 in minority populations of Newark, New Jersey. Int J Equity Health. 2020;19(1):93.

**eAppendix 1. Detailed search strategies used in this review**

**August 3^rd^ 2020 (last date searched)**

|  | Before deduplication | After deduplication |
| --- | --- | --- |
| Embase.com | 2354 | 2283 |
| PubMed | 2443 | 970 |
| Medline Ovid | 1687 | 14 |
| Cochrane Trials | 86 | 78 |
| Web-of-Science | 709 | 80 |
| Google Scholar | 200 | 139 |
| WHO Covid-19 Database | 105 | 37 |
| **Total** | **7584** | **3601** |

3983 duplicate records have been removed

Other sources: searching bibliographies of core articles, forward citation tracking
**Limits**: excluding animal studies, excluding conference abstracts, letters, notes, editorials

**Embase.com**

('covid 19'/exp OR 'coronavirus disease 2019'/exp OR 'severe acute respiratory syndrome coronavirus 2'/exp OR 'SARS-related coronavirus'/exp OR 'coronaviridae'/de OR 'coronavirinae'/exp OR 'coronavirus infection'/exp OR (coronavir* OR 'corona virus*' OR covid OR COVID19 OR nCoV OR '2019-novel*' OR 2019nCoV OR 2019‐CoV OR nCoV2019 OR 'pneumonia virus*' OR 'Severe Acute Respiratory Syndrome*' OR SARS OR SARSCoV*):ab,ti,kw) AND ('iron deficiency'/exp OR 'anemia'/exp OR 'transferrin'/de OR 'ferritin'/de OR 'iron binding protein'/exp OR 'erythropoietin'/de OR 'soluble transferrin receptor'/de OR 'hepcidin'/de OR 'haptoglobin'/de OR 'unsaturated iron binding capacity'/de OR 'erythrocyte'/exp OR 'erythrocyte protoporphyrin'/de OR 'erythrocyte parameters'/exp OR 'erythrocyte count'/de OR 'red cell distribution width'/de OR 'erythrocyte volume'/de OR 'mean corpuscular volume'/de OR 'mean corpuscular hemoglobin'/de OR 'mean corpuscular hemoglobin concentration'/de OR 'reticulocyte count'/exp OR 'hematocrit'/de OR 'hemoglobin'/exp OR 'hemoglobin blood level'/de OR 'clinical feature'/de OR 'biological marker'/de OR (anemi* OR anaemi* OR iron OR 'red cell*' OR 'red blood cell*' OR ((hemocyte* OR hemacyte* OR hematocyte*) NEAR/2 (count*)) OR reticulocyte* OR haematocrit OR hematocrit OR transferrin OR transferrins OR serotransferrin* OR siderophilin OR isotransferrin* OR apoferritin OR ferritin* OR isoferritin* OR immunoferritin* OR mycoferritin* OR proteoferrin* OR sanifer OR unifer OR erythropoieti* OR hematopoie* OR hemopoie* OR haematopoie* OR haemopoie* OR hepcidin* OR haptoglobin* OR UIBC OR (('free erythrocyte' OR normocyte OR 'red blood cell' OR 'red cell') NEAR/2 (protoporphyrin*)) OR erythrocyte* OR hemoglobin* OR haemoglobin* OR hemoglobulin* OR haemoglobulin* OR ferrohaemoglobin* OR ferrohemoglobin* OR ((clinical) NEAR/3 (characteristic* OR feature* OR predictor*)) OR biomarker* OR 'biological marker*'):ab,ti,kw) NOT ([animals]/lim NOT [humans]/lim) NOT ([Conference Abstract]/lim OR [Letter]/lim OR [Note]/lim OR [Editorial]/lim) AND [2019-3000]/py

**PubMed**

("Coronavirus"[Mesh] OR "Coronavirus Infections"[Mesh] OR "COVID-19" [Supplementary Concept] OR coronavir*[tiab] OR "corona virus*" [tiab] OR covid[tiab] OR nCoV[tiab] OR 2019-novel*[tiab] OR 2019nCoV[tiab] OR 2019‐CoV[tiab] OR nCoV2019[tiab] OR pneumonia virus*[tiab] OR "Severe Acute Respiratory Syndrome"[tiab] OR SARS[tiab] OR SARSCoV*[tiab]) AND ("Anemia"[Mesh] OR "Transferrin"[Mesh] OR "Ferritins"[Mesh] OR "Erythropoietin"[Mesh] OR "Receptors, Transferrin"[Mesh] OR "Hepcidins"[Mesh] OR "Haptoglobins"[Mesh] OR "Erythrocytes"[Mesh] OR "Erythrocyte Count"[Mesh] OR "Reticulocyte Count"[Mesh] OR "Erythrocyte Indices"[Mesh] OR "Erythrocyte Volume"[Mesh] OR "Iron-Binding Proteins"[Mesh] OR "Hematocrit"[Mesh] OR "Hemoglobins"[Mesh] OR "Biomarkers/blood"[Mesh] OR anemi*[tiab] OR anaemi*[tiab] OR iron[tiab] OR red cell*[tiab] OR red blood cell*[tiab] OR ((hemocyte*[tiab] OR hemacyte*[tiab] OR hematocyte*[tiab]) AND (count[tiab] OR counts[tiab])) OR reticulocyte*[tiab] OR haematocrit[tiab] OR hematocrit[tiab] OR transferrin[tiab] OR transferrins[tiab] OR serotransferrin*[tiab] OR siderophilin*[tiab] OR isotransferrin*[tiab] OR apoferritin*[tiab] OR ferritin*[tiab] OR isoferritin*[tiab] OR immunoferritin* OR mycoferritin* OR proteoferrin* OR sanifer OR unifer OR erythropoie*[tiab] OR hematopoie*[tiab] OR hemopoie*[tiab] OR haematopoie*[tiab] OR haemopoie*[tiab] OR hepcidin*[tiab] OR haptoglobin*[tiab] OR UIBC[tiab] OR (("free erythrocyte"[tiab] OR normocyte[tiab] OR "red blood cell" [tiab] OR "red cell" [tiab]) AND (protoporphyrin*[tiab])) OR erythrocyte*[tiab] OR hemoglobin*[tiab] OR haemoglobin*[tiab] OR hemoglobulin*[tiab] OR haemoglobulin*[tiab] OR ferrohaemoglobin*[tiab] OR ferrohemoglobin*[tiab] OR OR ((clinical[tiab]) AND (characteristic*[tiab] OR feature*[tiab] OR predictor*[tiab])) OR biomarker*[tiab] OR biological marker*[tiab]) NOT (animals[mh] NOT humans[mh]) NOT (letter[pt] OR news[pt] OR comment[pt] OR editorial[pt] OR congress[pt] OR abstracts[pt]) AND 2019 : 3000 [dp]

**[dp] = Publication date**

**Medline Ovid**

(**Covid-19 Expert Search limit for Ovid MEDLINE*) AND (exp anemia/ OR transferrin/ OR exp ferritins/ OR exp erythropoietin/ OR exp Receptors, Transferrin/ OR hepcidins/ OR haptoglobins/ OR unsaturated iron binding capacity/ OR exp erythrocytes/ OR exp erythrocyte count/ or reticulocyte count/ OR Erythrocyte Indices/ OR Erythrocyte Volume/ OR exp Iron-Binding Proteins/ OR Hematocrit/ OR exp hemoglobins/ OR exp Biomarkers/bl OR (anemi* OR anaemi* OR iron OR red cell* OR red blood cell* OR ((hemocyte* OR hemacyte* OR hematocyte*) ADJ2 (count*)) OR reticulocyte* OR haematocrit OR haematocrit OR transferrin OR transferrins OR serotransferrin* OR siderophilin OR isotransferrin* OR apoferritin OR ferritin* OR isoferritin* OR immunoferritin* OR mycoferritin* OR proteoferrin* OR sanifer OR unifer OR erythropoie* OR hematopoie* OR hemopoie* OR haematopoie* OR haemopoie* OR hepcidin* OR haptoglobin* OR UIBC OR ((free erythrocyte OR normocyte OR red blood cell OR red cell) ADJ2 (protoporphyrin*)) OR erythrocyte* OR hemoglobin* OR haemoglobin* OR hemoglobulin* OR haemoglobulin* OR ferrohaemoglobin* OR ferrohemoglobin* OR ((clinical) ADJ3 (characteristic* OR feature* OR predictor*)) OR biomarker* OR biological marker*).ab,ti,kw) NOT (exp animals/ NOT humans/) NOT (letter OR news OR comment* OR editorial* OR congres* OR abstract*).pt.

**http://tools.ovid.com/coronavirus : Wolters Kluwer, Expert Search Strategies COVID-19,
Ovid MEDLINE(R) All 1946 to present*

**Cochrane Central Register of Controlled Trials** (Issue 8 of 12, August 2020)

((coronavir* OR corona NEXT virus* OR covid OR COVID19 OR nCoV OR "2019-novel Cov" OR 2019nCoV OR 2019‐CoV OR nCoV2019 OR "pneumonia virus" OR "Severe Acute Respiratory Syndrome" OR SARS ):ab,ti,kw) AND ((anemi* OR anaemi* OR iron OR red NEXT cell* OR red-blood-cell* OR ((hemocyte* OR hemacyte* OR hematocyte* OR haemocyte*) NEAR/2 (count*)) OR reticulocyte* OR haematocrit OR hematocrit OR transferrin OR transferrins OR serotransferrin* OR siderophilin OR isotransferrin* OR apoferritin OR ferritin* OR isoferritin* OR immunoferritin* OR mycoferritin* OR proteoferrin* OR sanifer OR unifer OR erythropoieti* OR hematopoie* OR hemopoie* OR haematopoie* OR haemopoie* OR hepcidin* OR haptoglobin* OR UIBC OR (("free erythrocyte" OR normocyte OR "red blood cell" OR "red cell") NEAR/2 (protoporphyrin*)) OR erythrocyte* OR hemoglobin* OR haemoglobin* OR hemoglobulin* OR haemoglobulin* OR ferrohaemoglobin* OR ferrohemoglobin* OR ((clinical) NEAR/3 (characteristic* OR feature* OR predictor*)) OR biomarker* OR biological NEXT marker*):ab,ti,kw)
**Date range: 2019-2020**

**Web of Science Core Collection**

TS=(((coronavir* OR "corona virus*" OR covid OR COVID19 OR nCoV OR "2019-novel Cov" OR 2019nCoV OR 2019‐CoV OR nCoV2019 OR "pneumonia virus*" OR "Severe Acute Respiratory Syndrome" OR SARS )) AND ((anemi* OR anaemi* OR iron OR "red cell*" OR "red blood cell*" OR ((hemocyte* OR hemacyte* OR hematocyte* OR haemocyte*) NEAR/2 (count*)) OR reticulocyte* OR haematocrit OR hematocrit OR transferrin OR transferrins OR serotransferrin* OR siderophilin OR isotransferrin* OR apoferritin OR ferritin* OR isoferritin* OR immunoferritin* OR mycoferritin* OR proteoferrin* OR sanifer OR unifer OR erythropoieti* OR hematopoie* OR hemopoie* OR haematopoie* OR haemopoie* OR hepcidin* OR haptoglobin* OR UIBC OR (("free erythrocyte" OR normocyte OR "red blood cell" OR "red cell") NEAR/2 (protoporphyrin*)) OR erythrocyte* OR hemoglobin* OR haemoglobin* OR hemoglobulin* OR haemoglobulin* OR ferrohaemoglobin* OR ferrohemoglobin* OR ((clinical) NEAR/3 (characteristic* OR feature* OR predictor*)) OR biomarker* OR "biological marker*"))) AND DT=(article)

*PUBLICATION YEARS: ( 2020 OR 2019 )*

**Google scholar first 200 results** (out of 23’600 results: date limit: **Since 2019**)

covid|coronavirus|sars|cov anemia|anaemia|transferrin|ferritin|iron|erythropoietin|hemoglobin|haemoglobin|erythrocyte|erythrocytes|"red blood cell"|"red blood cells"|"RBC count"|RDW|"red cell volume"|MCV|MCH|MCHC|hepcidin|haptoglobin|reticulocyte

**WHO COVID-19** **Database**: Global literature on coronavirus disease
The WHO Database of publications on coronavirus disease (COVID-19). Available on

<https://www.who.int/emergencies/diseases/novel-coronavirus-2019/global-research-on-novel-coronavirus-2019-ncov>

tw:((anemia OR anaemia OR iron OR transferrin OR ferritin OR erythropoietin OR hemoglobin OR haemoglobin OR erythrocyte OR erythrocytes OR "red blood cells" OR "red blood cell" OR hepcidin OR haptoglobin OR "RBC count" OR rdw OR "red cell volume" OR mcv OR mch OR mchc OR "unsaturated iron binding" OR uibc)) AND db:("COVIDWHO" OR "ELSEVIER" OR "CNKI_Lanzhou" OR "WPRIM" OR "LILACS") AND (year_cluster:[2019 TO 2020])
